# Supplementary material for: Better together against genetic heterogeneity: A sex-combined joint main and interaction analysis of 290 quantitative traits in the UK Biobank
Source: PLoS Genet. 2024 Apr 24;20(4):e1011221. doi: 10.1371/journal.pgen.1011221 (PMC11073786; doi:10.1371/journal.pgen.1011221)
Supplement: S1 Appendix — (PDF) [file pgen.1011221.s001.pdf]

S1 Empirical equivalence between mega- and meta-analysis

We used the UK Biobank data (Application number: 64875) to conduct genetic mega- and meta-association studies of testosterone (UK Biobank Data Field: 30850), aiming to confirm their analytical equivalence [1,2]. We leveraged individual data from the UK Biobank to demonstrate that $T_{1,mega}$ is practically equivalent to $T_{1,metaL}$, and $T_{2,mega}$ is equivalent to $T_{2,metaQ}$. Following the sample quality control (QC) procedure employed in the Neale Lab's sex-stratified analysis, we conducted sex-stratified analysis of testosterone to get T_{Female} and T_{Male}, and validated our implementation by comparing them with T_{Female} and T_{Male} from the Neal's Lab. Subsequently, we computed $T_{1,metaL}$ and $T_{2,metaQ}$ based on T_{Female} and T_{Male}. Meanwhile, we also conducted mega-analysis to get $T_{1,mega}$. It should be noted that this is similar to the Neale Lab's "bothsex" traditional sex-combined mega-analysis, except for the assumption of sex-specific residual variance. We chose to focus on $T_{1,mega}$ due to its robustness under heteroskedasticity over $T_{bothsex}$. As our primary interest lies in verifying equivalence, we have streamlined the process for ease of computation and visual presentation, electing to use the 22,574 genotyped markers on Chromosome 17 which harbors SNPs with the strongest association for testosterone (in males) [4], instead of whole-genome imputed SNPs. Here we found that $T_{1,metaL}$ and $T_{1,mega}$ were empirically equivalent, as depicted in Fig i. This finding aligns with the theoretical results discussed by [2]. Similarly, we observed empirical equivalence between $T_{2,metaQ}$ and $T_{2,mega}$, a result that is consistent with the work of [1] and subsequent study in [3].

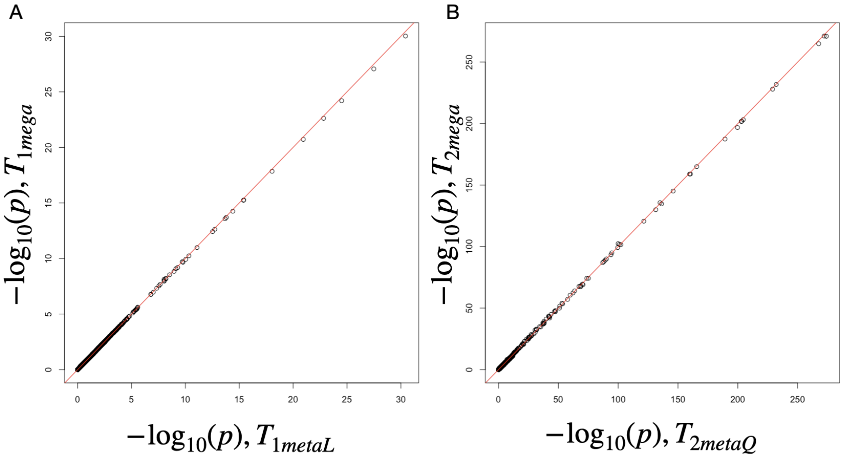

Fig i. Scatter plots to verify the practical equivalence between meta- and mega-analysis of testosterone. A: $-\log_{10}(p)$-value comparison between $T_{1,mega}$ (x-axis) and $T_{1,metaL}$ (y-axis); B: $-\log_{10}(p)$-value comparison between $T_{2,mega}$ (x-axis) and $T_{2,metaQ}$ (y-axis). We used the UK Biobank data to conduct T_{Female} (Female-only analysis), T_{Male} (Male only analysis) and $T_{1,mega}$ (sex-combined SNP main effect test) and $T_{2,mega}$ (sex-combined SNP main and SNP×sex interaction joint analysis). Subsequently, we computed $T_{1,metaL}$ (Traditional meta-analysis) and $T_{2,metaQ}$ (Omnibus meta-analysis) based on T_{Female} and T_{Male}. For ease of computation and visual presentation, we used 22,574 genotyped markers on Chromosome 17 as it harbors SNPs with the strongest association for testosterone in male [4], instead of whole-genome imputed SNPs. Red line indicates the reference main diagonal line.

References

1. Aschard H, Hancock DB, London SJ, Kraft P. Genome-wide meta-analysis of joint tests for genetic and gene-environment interaction effects. Human Heredity. 2010;70(4):292–300. doi:10.1159/000323318.

2. Lin DY, Zeng D. On the relative efficiency of using summary statistics versus individual-level data in meta-analysis. Biometrika. 2010;97:321–332. doi:10.1093/biomet/asq006.

3. Aschard H. A perspective on interaction effects in genetic association studies. Genetic Epidemiology. 2016;40(8):678–688.

4. Sinnott-Armstrong N, Naqvi S, Rivas M, Pritchard JK. GWAS of three molecular traits highlights core genes and pathways alongside a highly polygenic background. eLife. 2021;10:1–35. doi:10.7554/eLife.58615.
