# Supplementary material for: Better together against genetic heterogeneity: A sex-combined joint main and interaction analysis of 290 quantitative traits in the UK Biobank
Source: PLoS Genet. 2024 Apr 24;20(4):e1011221. doi: 10.1371/journal.pgen.1011221 (PMC11073786; doi:10.1371/journal.pgen.1011221)
Supplement: S2 Appendix — (PDF) [file pgen.1011221.s002.pdf]

S2 Simulation study design

We conducted extensive simulations to evaluate the performance of T_{Female}, T_{Min}, $T_{2,metaL}$, $T_{2,metaQ}$, $T_{1,mega}$, and $T_{2,mega}$, where T_{Min} is the minimal p-value approach, $T_{Min} = min(p_{Female}, p_{Male})$. Here p_M and p_F are independent of each other, and each is Unif(0, 1) distributed under the null of no association. Thus, the cumulative distribution function of T_{Min} is $F(x) = 1 - (1-x)^2$, which is used to obtain p-value for T_{Min}-based association analysis. Note that:

1. Without loss of generality, genetic effect in male was assumed to be no larger than that in female, T_{Male} was not explicitly examined but used to construct the summary statistics-based sex-combined analyses.

2. We excluded T_{Min} from the main text. In nearly all simulation scenarios, it was outperformed by both $T_{2,metaQ}$ and $T_{2,mega}$ (detailed results will be presented later).

3. We omitted T_{Diff} from our simulation studies, as our primary objective is to identify genetic associations that affect either sex, rather than specifically focusing on detecting SNP$times$ Sex interactions."

We considered the bi-allelic SNP of interest G is coded additively as in convention, and $\mathbb{P}(G=2) = maf^2$, $\mathbb{P}(G=1) = 2 \times maf \times (1-maf)$, and $\mathbb{P}(G=0) = (1-maf)^2$, where maf is the population minor allele frequency and takes values of $maf = 0.05,\ 0.1$ or 0.25. We also considered varying male-to-female sample size ratio, with $k = n_M/n_F$ taking values from $\{0.5, 1, 1.5, 2\}$, and $n_F = 5,000$. As power depends on both sample size and effect size, we did not empirically consider different n_F, as it does not affect the *relative* performance in power between the different methods. The sex-combined phenotype generating regression model can be reformulated, equivalently, as two separate simulation models for male and female, respectively, as

$$\text{male: } \mathbf{Y} = b_g \mathbf{G} + N(0,1), \text{ and}$$

$$\text{female: } \mathbf{Y} = b_s + (b_g + b_{gs})\mathbf{G} + N(0,1).$$

We fixed $b_s = 1$ so that sex explained about 20% of the total phenotypic variation. By varying b_g and b_{gs}, we were able to simulate phenotype data under different association scenarios including;

The null scenario: No genetic effects in both female and male.

To evaluate the empirical type I error rates, we fixed $b_g = b_{gs} = 0$ to generate data under the null hypothesis of no genetic effects of G on Y, in both female and male.

Alternative scenario A1: Homogeneous genetic effect between female and male.

In scenario A1, we fixed $b_{gs} = 0$ so that the true genetic model has no interaction effect, which is statistically equivalent with homogeneous genetic effect between female and male. This is the best-case scenario for $T_{1,mega}$ and the worst for $T_{2,mega}$, but we will show that power of $T_{2,mega}$ is competitive under A1, and $T_{2,metaQ}$ has the same performance as $T_{2,mega}$.

The range of b_g was $[0.06, 0.3]$, corresponding to the phenotypic variation explained by the genetic variable $(\beta_g^2 Var(G)/Var(y))$ in the range of $[0.14\%, 3.24\%]$. For completeness, we also let $b_g = 0$ so the power study also includes the null scenario, where we expect the empirical power to be at the nominal type I error level. Similar inclusion of the null was made when studying alternative scenarios A2 and A3 below.

Alternative scenario A2: Female-only genetic effect.

In scenario A2, we fixed $b_g = 0$ (no effect in male), and b_{gs} in the range of $[0.06, 0.3]$ (the effect in female). This setting is the best-case scenario for T_{Female}. But again, we will show that $T_{2,mega}$ and $T_{2,metaQ}$ are competitive, as compared with T_{Female}, and under A2 $T_{2,mega}$ and $T_{2,metaQ}$ also have the same empirical performance.

Alternative scenario A3: Heterogeneous genetic effect between female and male.

Here we fixed genetic effect in female at $b_g + b_{gs} = 0.15$, and b_g in the range of $[-0.6, 0.3]$ for the genetic effect in male. When $b_g < 0$, the effect directions differ between female and male. When $b_g > 0$, although the effect directions are the same, the effect magnitudes differ, unless $b_g = 0.15$.

Additionally, we conducted a sensitivity analysis under the following two settings;

Sensitive Study 1: Non-normal residual distributions.

Instead of the standard normal residual, we generated ϵ from student-t_4 and χ_4^2 to compare the performance of the testing methods when there are, respectively, excess kurtosis and skewness in the residual distributions.

Sensitive Study 2: Dominant genetic model.

Instead of the standard additive genetic model, we generated phenotypes from dominant models, which statistically also covers recessive models by switching the choice of the baseline allele. The working models remained additive, following the common GWAS practice.

Finally, although we do not expect the relative method performance to change when analyzing a binary trait, for completeness, we also generated binary phenotype data from $Bernoulli(\boldsymbol{p})$, where $\boldsymbol{p} = g^{-1}(b_g\mathbf{G} + b_s\mathbf{S} + b_{gs}\mathbf{G} \times \mathbf{S})$ and g is the logit link function.
