# Supplementary material for: Better together against genetic heterogeneity: A sex-combined joint main and interaction analysis of 290 quantitative traits in the UK Biobank
Source: PLoS Genet. 2024 Apr 24;20(4):e1011221. doi: 10.1371/journal.pgen.1011221 (PMC11073786; doi:10.1371/journal.pgen.1011221)
Supplement: S3 Appendix — (PDF) [file pgen.1011221.s003.pdf]

S3 Simulation results

Results here focus on power across different alternative scenarios, as all six tests are standard statistical tests derived directly or indirectly from regression and expected to be accurate, particularly when the trait is normally distributed. But for completeness, we report the empirical type I error rates under the null of no association and including the sensitivity analyses.

Empirical type I error rates

We used $R = 5 \times 10^6$ replications to evaluate the empirical type I error rates at the nominal level of $\alpha = 10^{-5}$. Results in Fig i show that, as expected, all six tests are accurate, and have the nominal level $\alpha = 10^{-5}$ covered by 95% binomial proportion confidence interval $(\hat{\alpha} \pm z\sqrt{\hat{\alpha}(1-\hat{\alpha})/R})$, where $\hat{\alpha}$ is empirical type I error. We note that $\alpha = 5 \times 10^{-8}$ is ideal but requires at least $R = 10^{10}$ replicates for accurate type I error evaluation, for each sample size considered, which is computational expensive, but $\alpha = 5 \times 10^{-8}$ was used for power study.

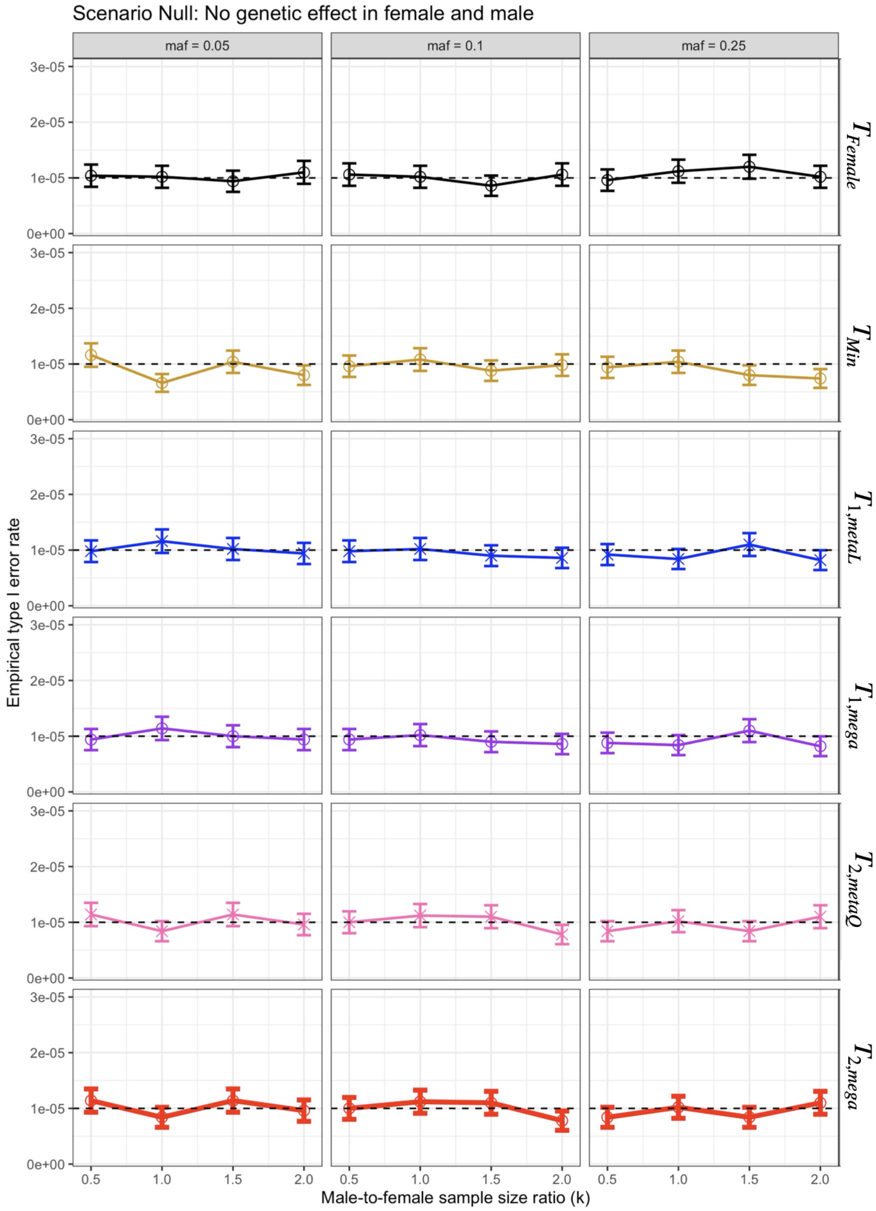

Fig i. The empirical type I error rates at the nominal $\alpha = 10^{-5}$ based on $R = 5 \times 10^6$ replications and their 95% confidence intervals under the null scenario, with $n_f = 5,000$, across different $k = n_m/n_f$ and MAF. Six association testing methods were evaluated: (1) The female-only SNP main effect test (T_{Female}); (2) The minimum p-value of female and male sex-stratified analysis (T_{Min}); (3) The traditional meta-analysis ($T_{1,metaL}$); (4) SNP main effect test ($T_{1,mega}$); (5) The omnibus meta-analysis ($T_{2,metaQ}$), the recommended test when only sex-stratified summary statistics are available; (6) SNP main and SNP×sex interaction joint analysis ($T_{2,mega}$). The dashed line indicates the nominal type I error rate of 1e-5. The 95% Binomial proportion confidence interval is constructed by formula: $\left(\hat{\alpha} - 1.96\sqrt{(1-\hat{\alpha})\hat{\alpha}/R}, \hat{\alpha} + 1.96\sqrt{(1-\hat{\alpha})\hat{\alpha}/R}\right)$, where $\hat{\alpha}$ is the empirical type I error rate.

Power comparison

Fig ii shows the empirical powers of the six testing methods, respectively, under the three alternative scenarios, A1, A2 and A3, where the empirical powers, at the genome-wide significance level of 5×10^{-8}, were obtained from 10^5 replicates. Result presentation here focuses on sample sizes of $n_F = n_M = 5,000$. Results for unbalanced sample size ratio with $k \in \{0.5, 1.5, 2\}$ are characteristically similar in that the relative method performance stays the same (Fig iii-Fig v).

In Scenario A1 where genetic effects are the same between female and male, it is easy to see that this is the best case-scenario for $T_{1,mega}$, which jointly analyzes all samples available through a (correctly-specified) main-effect-only regression model. Similarly, T_{Female} should have the lowest power, as the female-only analysis ignores the $n_M = k \cdot n_F$ of the total available n samples. Additionally, when there is no effect heterogeneity, meta-analysis (linearly combining Z_{Female} and Z_{Male}) is as efficient as mega-analysis [1]. Thus, $T_{1,metaL}$ has the same power as $T_{1,mega}$ as expected; the two power curves overlap in Fig ii Scenario A1.

As there is no interaction effect in Scenario A1, the 2 df $T_{2,mega}$ test, jointly testing both the main and interaction effects, is expected to be less powerful than $T_{1,mega}$. However, results in Fig ii Scenario A1 show that the loss of power is marginal, suggesting the robustness of $T_{2,mega}$ even under the scenario of no interaction effect. Additionally, $T_{2,mega}$ is noticeably more powerful than the minimum-p value approach of T_{Min}. Finally, the power curve of $T_{2,mega}$, interestingly, overlaps with that of $T_{2,metaQ}$ (quadratically combining Z_F^2 and Z_M^2.)

In Scenario A2, where the genetic effect exists only in female, the female-only sex-stratified test T_{Female} is most powerful as expected, and $T_{1,mega}$ is the least powerful with significant loss of power; power of $T_{1,metaL}$ is practically identical to that of $T_{1,mega}$ as expected. Compared with Scenario A1, the performances of T_{Female} and $T_{1,mega}$ (and $T_{1,metaL}$) here in Scenario A2 are reversed, suggesting that neither method is robust against different alternatives, which are unknown in practice. Importantly, $T_{2,metaQ}$ is also competitive in this case, with power only slightly smaller than that of the most powerful method of T_{Female}. Interestingly, same as in Scenario A1, the power curve of $T_{2,mega}$ overlaps with that of $T_{2,metaQ}$ in Scenario A2 as well.

In Scenario A3 where genetic effects exist in both female and male but differ in magnitude and/or direction, it reasonable to predict that no method dominates the others as confirmed by results in Fig ii. Specifically, (I) When the genetic effect in male is close to zero, the relative performance of the different methods is, as expected, similar to that observed scenario A2: The female-only sex-stratified test T_{Female} has the highest power, but $T_{2,metaQ}$ and $T_{2,mega}$ are competitive. (II) When the genetic effect in male is close to 0.15 (i.e. without effect heterogeneity between male and female), the relative performance is similar to that in scenario A1: $T_{1,mega}$ and $T_{1,metaL}$ have the highest power, but $T_{2,metaQ}$ and $T_{2,mega}$ are competitive. (III) When the genetic effect in male is close to -0.15 (i.e. with severe effect heterogeneity to the extend of opposite effect directions), $T_{2,metaQ}$ and $T_{2,mega}$ are clearly more powerful than the other methods.

Finally, across the range of parameter values under Scenario A3, the empirical powers of $T_{2,mega}$ and $T_{2,metaQ}$ are the same as under A1 and A2. Thus, although theoretical justification is needed, our simulation results suggest that the 2 df interaction mega-analysis can be obtained from sex-stratified summary statistics through $T_{2,metaQ}$ (quadratically combining Z^2_{Female} and Z^2_{Male}).

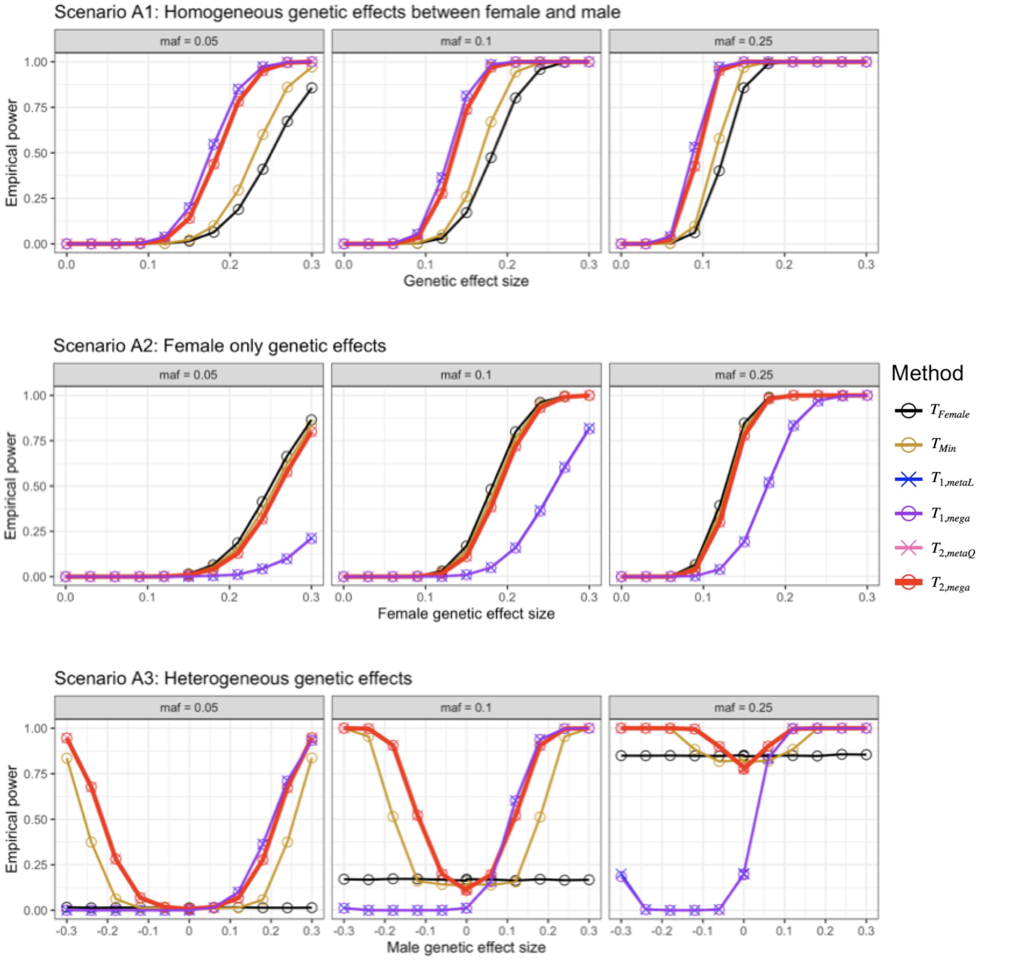

Fig ii. Power comparison at $\alpha = 5 \times 10^{-8}$ with female sample size $n_F = 5,000$ and $k = 1$ ($n_M = 5,000$) under the three alternative scenarios, A1, A2 and A3, stratified by the MAF. A1: Homogeneous genetic effect between female and male, and the genetic effect sizes ranged from 0 to 0.3; A2: Female-only genetic effect, and the genetic effect sizes ranged from 0 to 0.3; A3: Heterogeneous genetic effect between female and male, the genetic effect in female was kept at 0.15, while the effect in male ranged from -0.3 to 0.3. Six association testing methods were evaluated: (1) The female-only SNP main effect test (T_{Female}); (2) The minimum p-value of sex-stratified analysis (T_{Min}); (3) The traditional meta-analysis ($T_{1,metaL}$); (4) SNP main effect test ($T_{1,mega}$); (5) The omnibus meta-analysis ($T_{2,metaQ}$), the recommended test when only sex-stratified summary statistics are available; (6) SNP main and SNP×sex interaction joint analysis ($T_{2,mega}$). Results for other $k = n_M/n_F$ male-to-female ratios are shown in Fig iii-Fig v.

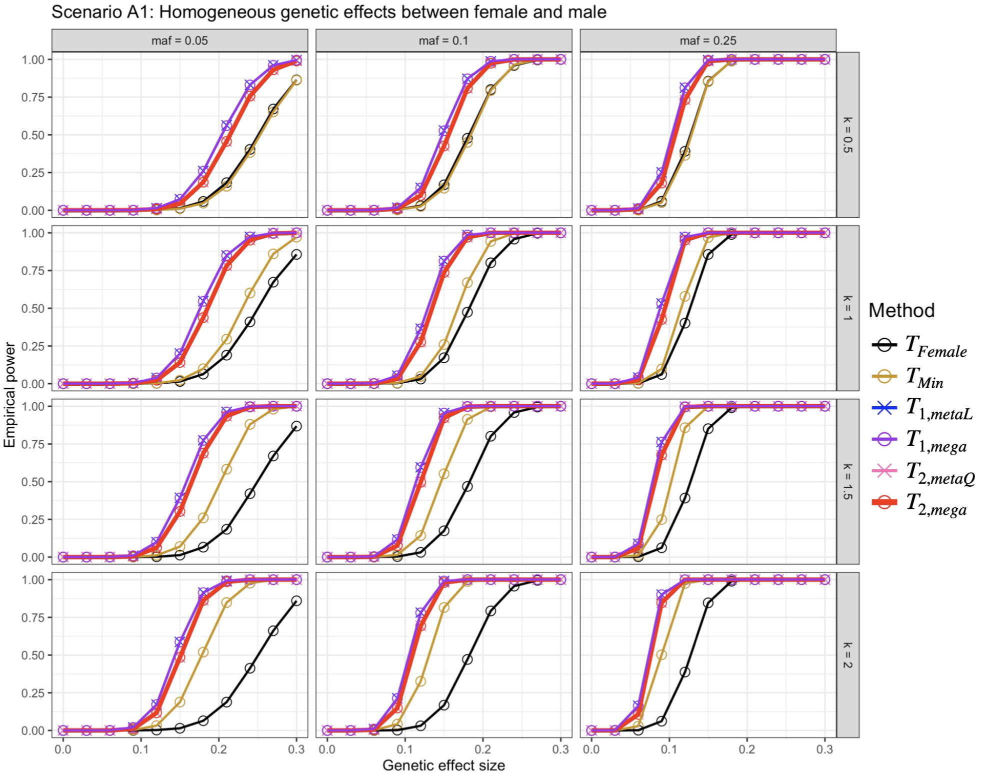

Fig iii. Power comparison at $\alpha = 5\text{e-}8$ with female sample sizes $n_f = 5,000$ under the alternative scenario A1 (homogeneous genetic effects between female and male), stratified by MAF and male-to-female sample size. The genetic effect sizes ranged from 0 to 0.3. Six association testing methods were evaluated: (1) The female-only SNP main effect test (T_{Female}); (2) The minimum p-value of sex-stratified analysis (T_{Min}); (3) The traditional meta-analysis ($T_{1,metaL}$); (4) SNP main effect test ($T_{1,mega}$); (5) The omnibus meta-analysis ($T_{2,metaQ}$), the recommended test when only sex-stratified summary statistics are available; (6) SNP main and SNP×sex interaction joint analysis ($T_{2,mega}$).

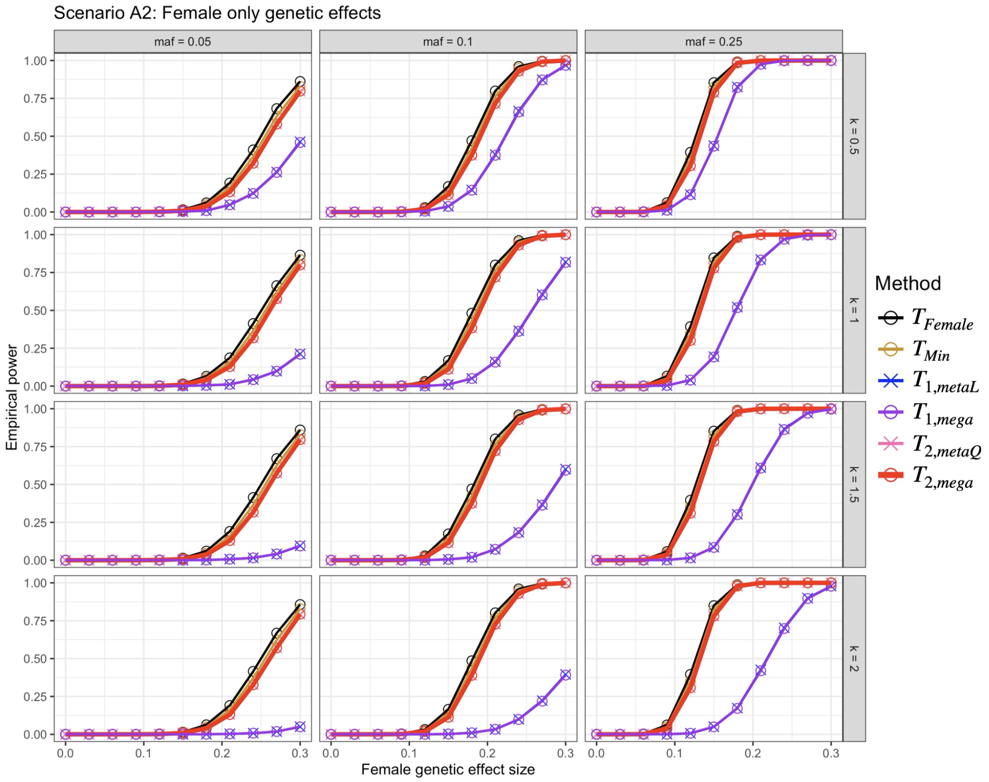

Fig iv. Power comparison at $\alpha = $ 5e-8 with female sample sizes $n_f = 5,000$ under the alternative scenario A2 (female only genetic effect), stratified by MAF and male-to-female sample size. The genetic effect sizes ranged from 0 to 0.3. Six association testing methods were evaluated: (1) The female-only SNP main effect test (T_{Female}); (2) The minimum p-value of sex-stratified analysis (T_{Min}); (3) The traditional meta-analysis ($T_{1,metaL}$); (4) SNP main effect test ($T_{1,mega}$); (5) The omnibus meta-analysis ($T_{2,metaQ}$), the recommended test when only sex-stratified summary statistics are available; (6) SNP main and SNP×sex interaction joint analysis ($T_{2,mega}$).

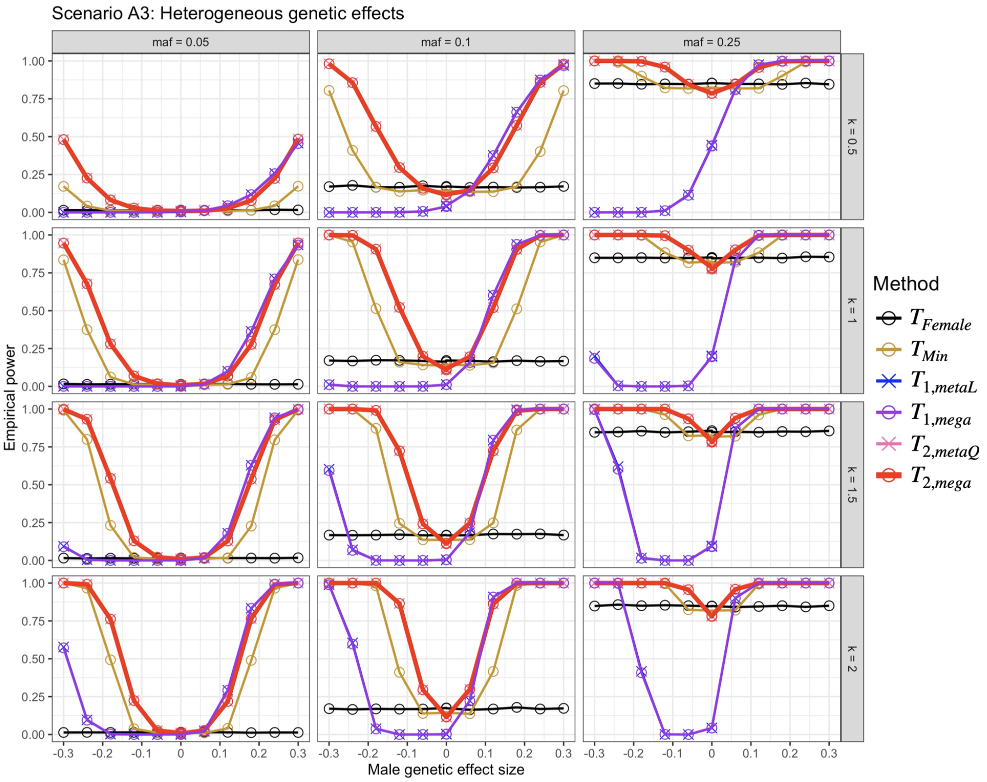

Fig v. Power comparison at $\alpha =$ 5e-8 with female sample sizes $n_f = 5,000$ under the alternative scenario A3 (heterogeneous effects), stratified by MAF and male-to-female sample size. The genetic effect in female was kept at 0.15, while the effect in male ranged from -0.3 to 0.3. Six association testing methods were evaluated: (1) The female-only SNP main effect test (T_{Female}); (2) The minimum p-value of sex-stratified analysis (T_{Min}); (3) The traditional meta-analysis ($T_{1,metaL}$); (4) SNP main effect test ($T_{1,mega}$); (5) The omnibus meta-analysis ($T_{2,metaQ}$), the recommended test when only sex-stratified summary statistics are available; (6) SNP main and SNP×sex interaction joint analysis ($T_{2,mega}$).

Sensitivity studies with model mis-specification and binary outcome

When the error distribution is t_4 or χ^2_4, given our simulation sample size ($n_F = 5,000$ with male-to-female sample size ratio of 0.5, 1, 1.5, or 2), the empirical type I error rates can be slightly inflated when MAF is low at 0.05, for all six tests examined (Fig vi). This is, however, not surprising as the convergence of the least square estimator's sampling distribution is slower when the residual distribution is non-normal, particularly asymmetrical (e.g. χ^2_4) [2]. Subsequently, we did not consider power evaluation with non-normal residuals.

When the phenotype values are simulated from a dominant genetic model while the working model is additive, or the trait is binary, the type I error control results are similar to above (Fig vii and Fig viii).

Finally, across the three alternatives considered, the relative performances of the different tests for dominant generating models (Fig ix, the right panel) and for binary traits (Fig x, left panel for additive model and right panel for dominant model) remain consistent with those observed from normally distributed traits and additive genetic models, as shown Fig ii and Fig iii-Fig v.

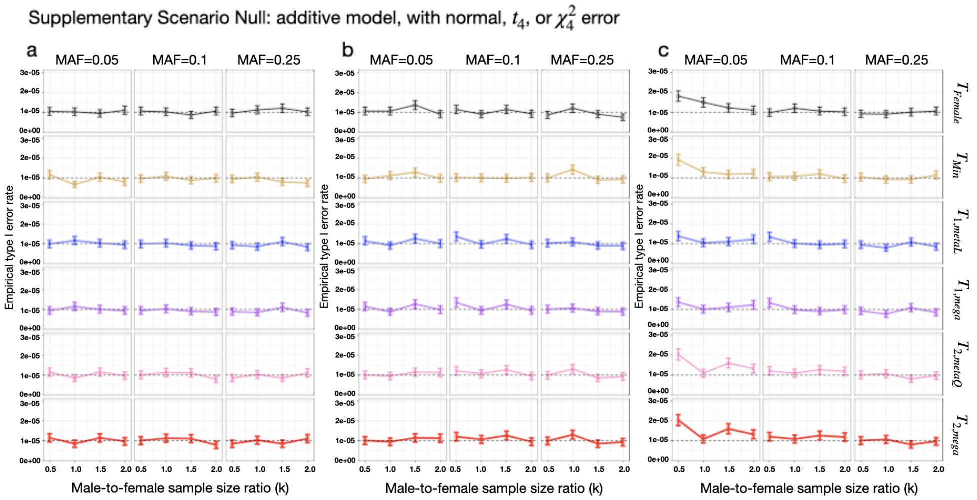

Fig vi. Results of sensitivity study: non-normal residuals. The empirical type I error rates at the nominal $\alpha = $ 1e-5 based on $R = 5 \times 10^6$ replications and their 95% confidence intervals under the null scenario, with $n_f = n_m = 5,000$, stratified by residual distributions (a) standard normal, (b) t_4 and (c) χ^2_4. The genotypes simulated under additive model. Six association testing methods were evaluated: (1) The female-only SNP main effect test (T_{Female}); (2) The minimum p-value of sex-stratified analysis (T_{Min}); (3) The traditional meta-analysis ($T_{1,metaL}$); (4) SNP main effect test ($T_{1,mega}$); (5) The omnibus meta-analysis ($T_{2,metaQ}$), the recommended test when only sex-stratified summary statistics are available; (6) SNP main and SNP×sex interaction joint analysis ($T_{2,mega}$). The dashed line indicates the nominal type I error rate of 1e-5. The 95% Binomial proportion confidence interval is constructed by formula: $\left(\hat{\alpha} - 1.96\sqrt{\frac{(1-\hat{\alpha})\hat{\alpha}}{R}}, \hat{\alpha} + 1.96\sqrt{\frac{(1-\hat{\alpha})\hat{\alpha}}{R}}\right)$, where $\hat{\alpha}$ is the empirical type I error rate.

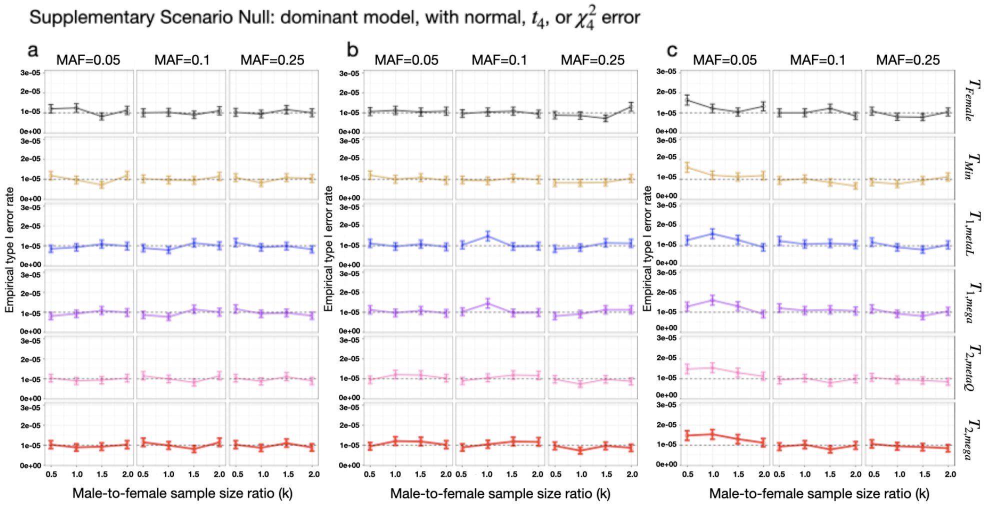

Fig vii. Results of sensitivity study: non-additive generating model. The empirical type I error rates at the nominal $\alpha = $ 1e-5 based on $R = 5 \times 10^6$ replications and their 95% confidence intervals under the null scenario, with $n_f = n_m = 5,000$, stratified by residual distributions (a) standard normal, (b) t_4 and (c) χ^2_4. The genotypes simulated under dominant model. Six association testing methods were evaluated: (1) The female-only SNP main effect test (T_{Female}); (2) The minimum p-value of sex-stratified analysis (T_{Min}); (3) The traditional meta-analysis ($T_{1,metaL}$); (4) SNP main effect test ($T_{1,mega}$); (5) The omnibus meta-analysis ($T_{2,metaQ}$), the recommended test when only sex-stratified summary statistics are available; (6) SNP main and SNP×sex interaction joint analysis ($T_{2,mega}$). The dashed line indicates the nominal type I error rate at 10^{-5}. The 95% Binomial proportion confidence interval is constructed by formula: $\left(\hat{\alpha} - 1.96\sqrt{\frac{(1-\hat{\alpha})\hat{\alpha}}{R}}, \hat{\alpha} + 1.96\sqrt{\frac{(1-\hat{\alpha})\hat{\alpha}}{R}}\right)$, where $\hat{\alpha}$ is the empirical type I error rate.

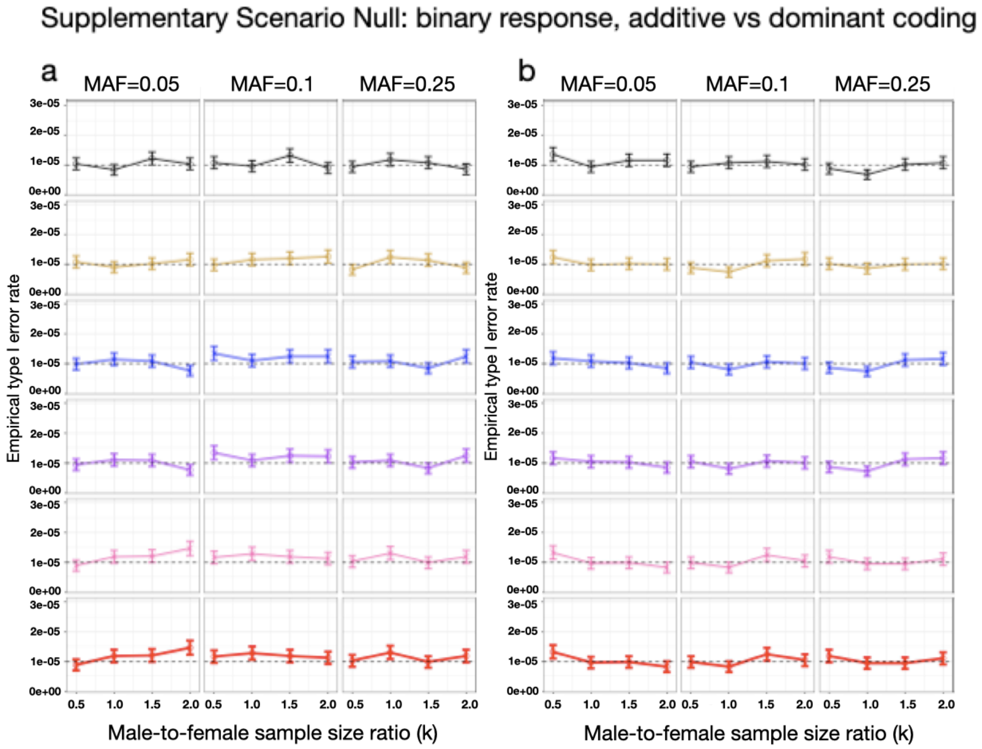

Fig viii. Results of sensitivity study: binary trait and non-additive generating model. The empirical type I error rates with binary response at the nominal $\alpha = 1\text{e-}5$ based on $R = 5 \times 10^6$ replications and their 95% confidence intervals under the null scenario, with $n_f = n_m = 5,000$. We simulated data from (a) additive and (b) dominant genetic models respectively, although we assume additive model for association testing. Six association testing methods were evaluated: (1) The female-only SNP main effect test (T_{Female}); (2) The minimum p-value of sex-stratified analysis (T_{Min}); (3) The traditional meta-analysis ($T_{1,metaL}$); (4) SNP main effect test ($T_{1,mega}$); (5) The omnibus meta-analysis ($T_{2,metaQ}$), the recommended test when only sex-stratified summary statistics are available; (6) SNP main and SNP×sex interaction joint analysis ($T_{2,mega}$). The dashed line indicates the nominal type I error rate at 10^{-5}. The 95% Binomial proportion confidence interval is constructed by formula: $\left(\hat{\alpha} - 1.96\sqrt{\frac{(1-\hat{\alpha})\hat{\alpha}}{R}}, \hat{\alpha} + 1.96\sqrt{\frac{(1-\hat{\alpha})\hat{\alpha}}{R}}\right)$, where $\hat{\alpha}$ is the empirical type I error rate.

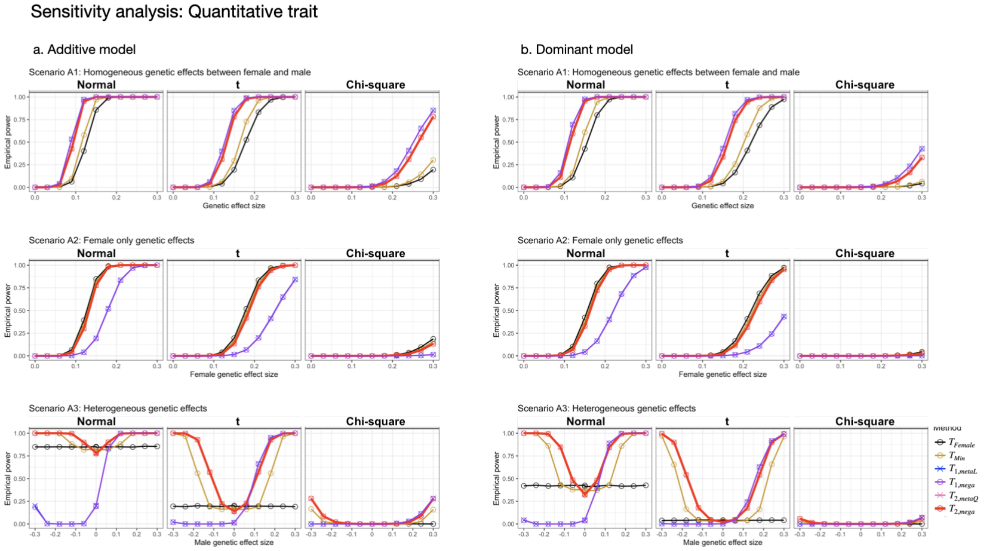

Fig ix. Quantitative trait power at α = 5e-8 under the three alternative scenarios, stratified by error distributions and genetic models. Sample sizes $n_f = n_m = 5,000$, and $MAF = 0.25$. The columns correspond to the additive and dominant genetic models respectively, although we assume additive model for association testing. Six association testing methods were evaluated: (1) The female-only SNP main effect test (T_{Female}); (2) The minimum p-value of sex-stratified analysis (T_{Min}); (3) The traditional meta-analysis ($T_{1,metaL}$); (4) SNP main effect test ($T_{1,mega}$); (5) The omnibus meta-analysis ($T_{2,metaQ}$), the recommended test when only sex-stratified summary statistics are available; (6) SNP main and SNP×sex interaction joint analysis ($T_{2,mega}$).

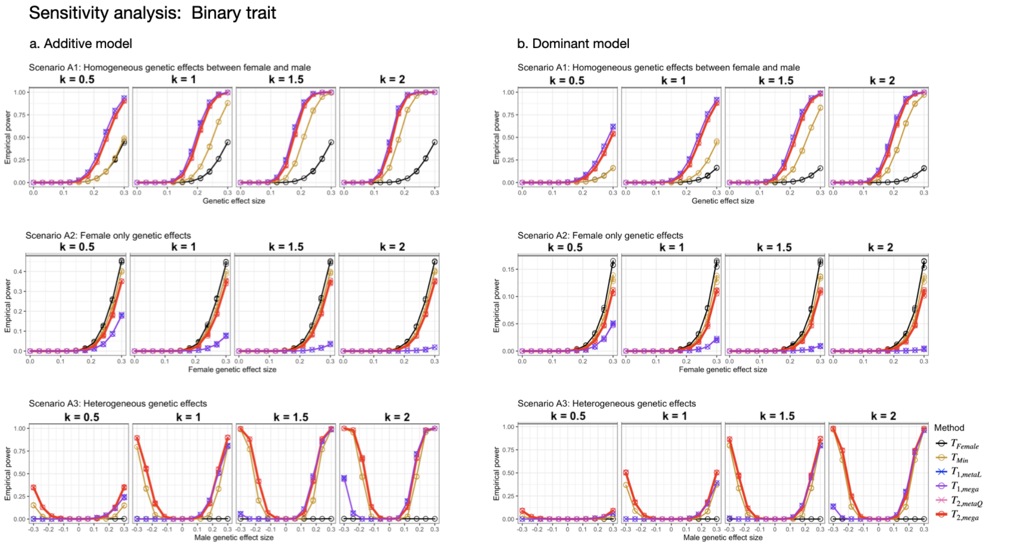

Fig x. Binary trait power at $\alpha = $ 5e-8 under the three alternative scenarios, stratified by genetic models and female-to-male sample size ratio (k). Sample sizes $n_f = 5,000$, $n_m = n_f \times k$, and $MAF = 0.25$. The columns correspond to the additive and dominant genetic models respectively, although we assume additive model for association testing. Six association testing methods were evaluated: (1) The female-only SNP main effect test (T_{Female}); (2) The minimum p-value of sex-stratified analysis (T_{Min}); (3) The traditional meta-analysis ($T_{1,metaL}$); (4) SNP main effect test ($T_{1,mega}$); (5) The omnibus meta-analysis ($T_{2,metaQ}$), the recommended test when only sex-stratified summary statistics are available; (6) SNP main and SNP×sex interaction joint analysis ($T_{2,mega}$).

References

1. Lin DY, Zeng D. On the relative efficiency of using summary statistics versus individual-level data in meta-analysis. Biometrika. 2010;97:321–332. doi:10.1093/biomet/asq006.

2. Lehmann EL. Elements of large-sample theory. Springer Texts in Statistics. New York, NY: Springer; 1999.
