# Supplementary material for: Better together against genetic heterogeneity: A sex-combined joint main and interaction analysis of 290 quantitative traits in the UK Biobank
Source: PLoS Genet. 2024 Apr 24;20(4):e1011221. doi: 10.1371/journal.pgen.1011221 (PMC11073786; doi:10.1371/journal.pgen.1011221)
Supplement: S4 Appendix — (PDF) [file pgen.1011221.s004.pdf]

S4 Comparison of number of independent SNPs

We conducted LD clumping to remove redundant signals and compare the numbers of independent SNPs identified by $T_{2,metaQ}$ and $T_{1,metaL}$ for each trait. In contrast to the patterns in comparison of number of (unpruned) SNPs (Figure 3A), multiple phenotypes have excessive independent SNPs identified from $T_{2,metaQ}$ over $T_{1,metaL}$ (Figure 3B), and such excessive findings are robust to the parameter settings in LD clumping (Fig i).

To understand the sources and genuineness of these excessive findings, we conducted a comprehensive analysis check on the five phenotypes with more independent genome-wide significant SNPs identified from $T_{2,metaQ}$ than those from $T_{1,metaL}$ and at least 50 independent genome-wide significant SNPs in both methods, namely Testosterone (nmol/L) (UKB Data Field: 30850), Pulse wave Arterial Stiffness index (UKB Data Field: 21021), High light scatter reticulocyte percentage (UKB Data Field: 30290), Lymphocyte count (UKB Data Field: 30120) and Sex hormone binding globulin (SHGB; UKB Data Field: 30830).

Fig ii shows that the excessive findings in $T_{2,metaQ}$ for testosterone compared to $T_{1,metaL}$ are predominantly SNPs with larger effects in males than in females. While for Pulse Wave Arterial Stiffness Index, High Light Scatter Reticulocyte Percentage, and Lymphocyte Count, the excessive findings in $T_{2,metaQ}$ compared to $T_{1,metaL}$ consist of SNPs with differences in point estimates of sex-specific effects. Finally, for SHBG the excessive findings includes SNPs with larger effect point estimates in females than in males.

Since all five phenotypes exhibit skewed distributions (Fig iii), and four of these phenotypes (excluding SHBG) feature extreme values (falling outside the range of mean $\pm$ 10 standard deviations in the combined samples; Fig iv), we compared the results between analysis using the raw phenotypes and the inverse normal transformed (IRNT) phenotypes, aiming to determine whether the excessive findings were driven by the non-normality of phenotypic residuals or by the presence of outlier samples with extreme phenotype values.

When comparing the numbers of independent SNPs identified using raw and IRNT phenotypes (S1 Table), it is worth noting that, in contrast to the analytical advantages associated with increased statistical power for inverse normal transformed phenotypes [1], very few genome-wide significant loci remained for the Pulse Wave Arterial Stiffness index after the IRNT transformation. This suggests that majority of the signals detected using raw Pulse Wave Arterial Stiffness index phenotype may be susceptible to type I errors. Indeed, by stratifying the GWAS results with MAF, we observed a deflated genomic inflation factor (λ_{GC}) for raw phenotype-associated SNP with relatively rare MAF in (0.01, 0.05] in either sex. The artifact was driven by the abundance of GWAS hits with small MAF (Fig vi) and was removed after IRNT (S1 Table). We therefore hypothesize that the excessive gain from $T_{2,metaQ}$ over $T_{1,metaL}$ with raw Pulse Wave Arterial Stiffness index phenotype may be driven by outlier samples carrying extreme phenotype values and rare variants.

For testosterone, we observed that the numbers of independent genome-wide significant SNPs increased for IRNT, and $T_{2,metaQ}$ remains advantageous over $T_{1,metaL}$ (S1 Table) with no enrichment in rare variants (Fig v), implying that the excessive gain from $T_{2,metaQ}$ over $T_{1,metaL}$ in GWAS with raw testosterone are SNPs with sex-specific effects.

For the rest three phenotypes, High light scatter reticulocyte percentage, Lymphocyte count and SHBG (nmol/L), analysis with IRNT phenotypes yields more independent SNPs from $T_{1,metaL}$ than $T_{2,metaQ}$ (S1 Table) with balanced distributions of MAF for identified SNPs (Fig vii-Fig ix), suggesting that the excessive gains from $T_{2,metaQ}$ over $T_{1,metaL}$ with raw phenotypes may be due to the non-normality of phenotypic residuals.

We also noticed that recent analysis [2] reported high light scatter reticulocyte percentage as an extreme example where analysis of the genetic dominant effect on raw phenotype had artifacts including poorly calibrated QQ-plot, an abundance of spurious signals and reduced genomic control inflation factor (GC = 0.862). In our analysis focusing on the genetic main and interaction effects, all five methods maintained acceptable GC values (1.05 $\sim$ 1.10; except for the aforementioned deflation for Pulse wave Arterial Stiffness index due to rare variants).

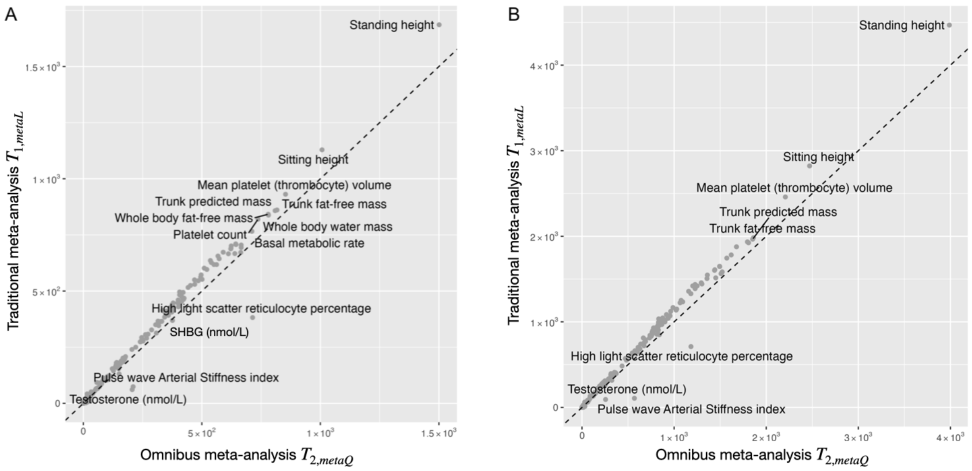

Fig i. Comparison of the numbers of genome-wide significant independent loci identified by the traditional meta-analysis $T_{1,metaL}$ (y-axis) and the omnibus meta-analysis $T_{2,metaQ}$ (x-axis) for each of the 290 traits analyzed. The definition for independent loci is available in the Materials and methods. Here LD is defined with a physical distance of 10MB in A and 100KB in B. Plot A and S12 Fig are plotted with the same data. The dashed line indicates the reference main diagonal line.

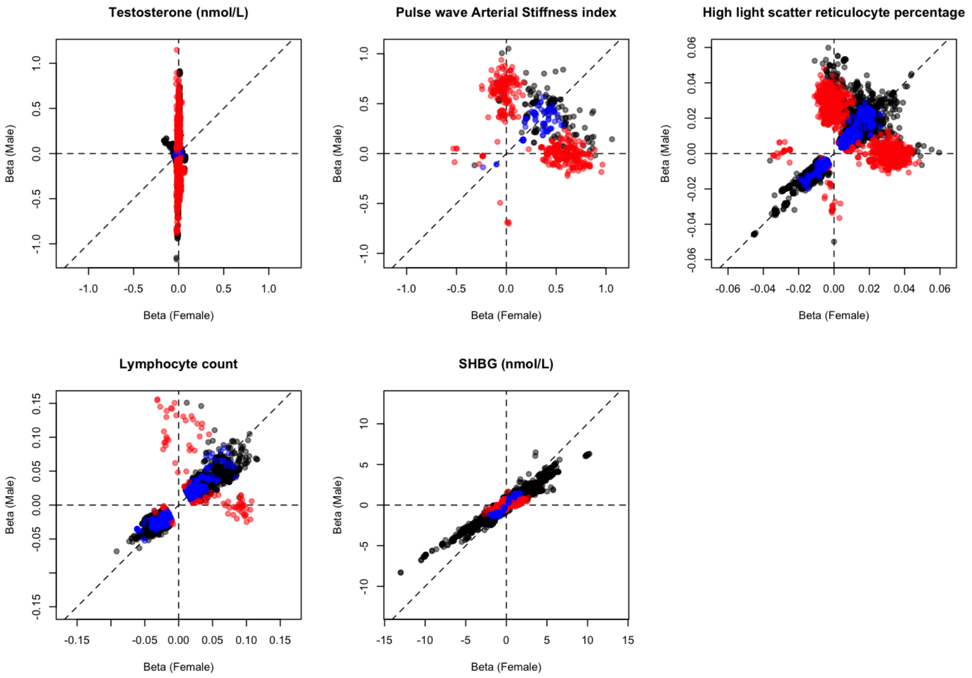

Fig ii. Sex-stratified genetic effect comparison for five phenotypes with more independent genome-wide significant SNPs identified from $T_{2,metaQ}$ than those from $T_{1,metaL}$, and present at least 50 independent genome-wide significant SNPs in both methods. $T_{1,metaL}$: Traditional meta-analysis; $T_{2,metaQ}$: Omnibus meta-analysis. The plotted points correspond to the set of independent SNPs identified by either $T_{2,metaQ}$ or $T_{1,metaL}$. Red points denote SNPs uniquely identified by $T_{2,metaQ}$, while blue points indicate SNPs that were uniquely identified by $T_{1,metaL}$. Black points represent SNPs identified by both $T_{1,metaL}$ and $T_{2,metaQ}$. The dashed lines indicate the reference lines at $x = 0$, $y = 0$ and $x = y$.

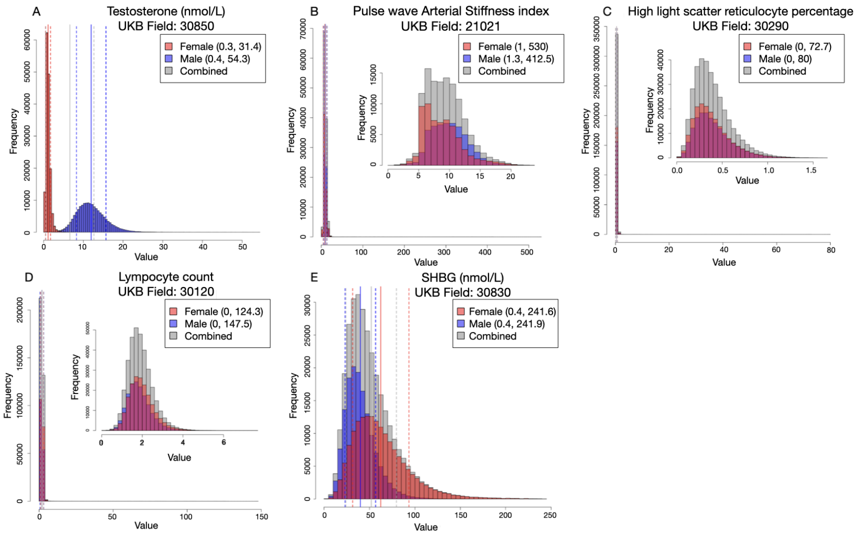

Fig iii. Sex-stratified phenotype distributions for five phenotypes with more independent genome-wide significant SNPs identified from $T_{2,metaQ}$ than those from $T_{1,metaL}$, and present at least 50 independent genome-wide significant SNPs in both methods. $T_{1,metaL}$: Traditional meta-analysis; $T_{2,metaQ}$: Omnibus meta-analysis. The sex-stratified phenotype distribution is based on a cohort of up to 361,194 participants (194,174 females and 167,020 males) included in the Neale lab's UK Biobank GWAS round 2. The sub-histograms in Figures B, C, and D show the histograms in the range of 0 to 99% quantiles of the phenotypes. The vertical solid and dashed lines indicate sample mean ± 1SD, respectively.

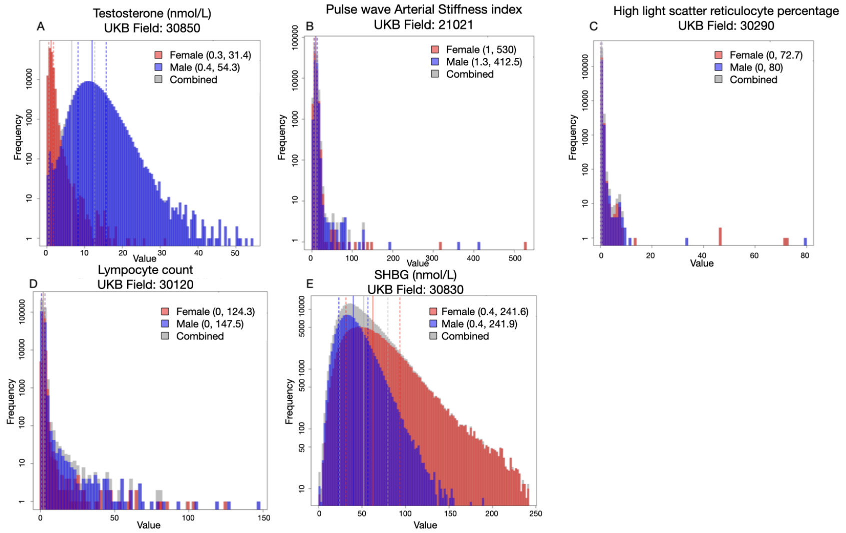

Fig iv. Sex-stratified phenotype distributions for five phenotypes with more independent genome-wide significant SNPs identified from $T_{2,metaQ}$ than those from $T_{1,metaL}$, and present at least 50 independent genome-wide significant SNPs in both methods. $T_{1,metaL}$: Traditional meta-analysis; $T_{2,metaQ}$: Omnibus meta-analysis. The y-axis is set to log-scale to provide a zoomed-in visualization of extreme values. The sex-stratified phenotype distribution is based on a cohort of up to 361,194 participants (194,174 females and 167,020 males) included in the Neale lab's UK Biobank GWAS round 2. The vertical solid and dashed lines indicate sample mean and sample mean± 1SD, respectively.

Tab i. Comparison of independent SNPs identified from raw and IRNT phenotypes

Phenotype	Raw					IRNT				
	T_{Female}	T_{Male}	T_{Diff}	$T_{1,metaL}$	$T_{2,metaQ}$	T_{Female}	T_{Male}	T_{Diff}	$T_{1,metaL}$	$T_{2,metaQ}$
Testosterone (nmol/L)	55	173	168	61	206	102	184	123	151	256
Pulse wave Arterial Stiffness index	165	115	19	74	210	0	1	0	4	2
High light scatter reticulocyte percentage	381	558	73	382	714	269	246	1	539	472
Lymphocyte count	106	60	10	131	151	280	208	0	582	507
SHBG (nmol/L)	173	211	22	369	376	198	249	14	464	439

Comparison of the numbers of independent genome-wide significant ($p < 5 \times 10^{-8}$) SNPs associated with the raw and inverse normal transformed phenotypes of the five phenotypes with excessive discoveries from $T_{2,metaQ}$. Here LD are defined within physical distance of 100kb.

Tab ii. Genomic inflation factor (GC lambda) stratified by minor allele frequency (MAF) for Pulse wave Arterial Stiffness index

**MAF stratification***	**GC Lambda**			
Raw phenotype	T_{Female}	T_{Male}	$T_{1,metaL}$	$T_{2,metaQ}$
All	0.961	0.979	1.021	0.961
Common	1.038	1.045	1.069	1.057
Moderate	0.968	0.988	1.024	0.969
Rare	0.823	0.852	0.927	0.781
IRNT phenotype				
All	1.037	1.045	1.084	1.069
Common	1.052	1.059	1.118	1.096
Moderate	1.016	1.025	1.066	1.048
Rare	1.016	1.025	1.026	1.026

"Common": Sex-specific MAF > 0.1 in both sexes; "Moderate": Sex-specific MAF > 0.05 in both sexes and ≤ 0.1 in either sex; "Rare": Sex-specific MAF > 0.01 in both sexes and ≤ 0.05 in either sex

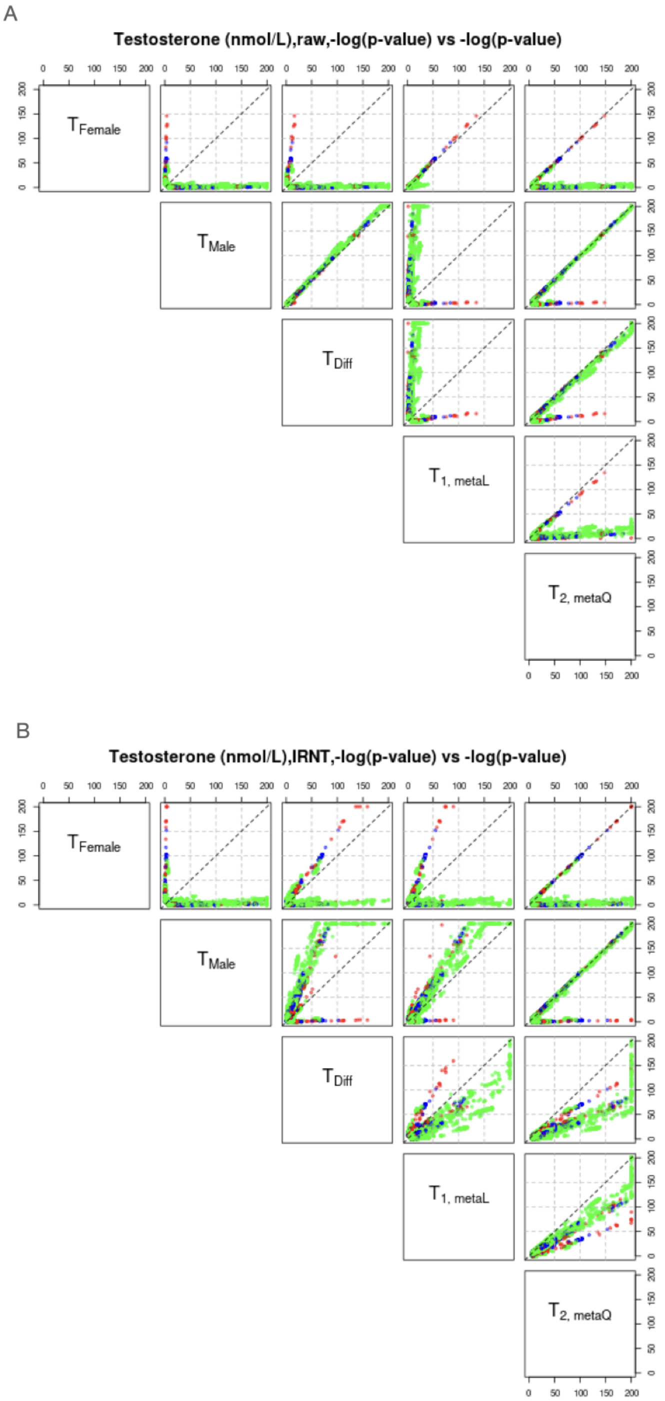

Fig v. Pairwise scatter plots of the genome-wide significant $(p < 5 \times 10^{-8})$ **SNPs for testosterone GWAS with raw (A) and inverse normal transformed (B) phenotypes detected by any of the five testing methods considered.**

The five association methods include T_{Female} (Female-only analysis), T_{Male} (Male-only analysis), T_{Diff} (SNP-sex interaction-only test), $T_{1,metaL}$ (the traditional sex-combined meta-analysis), and $T_{2,metaQ}$ (the omnibus meta-analysis); see Table 2 for method details. The sex-stratified GWAS summary statistics come from the Neale lab's UK Biobank GWAS round 2, which included a cohort of up to 361,194 participants (312,102 in testosterone GWAS, 154,364 females and 157,738 males). Axes depict $-\log_{10} p$-values for each pair of tests. SNPs are color-coded as follows: SNPs with sex-specific MAF > 0.1 in both sexes are in green; SNPs with sex-specific MAF between 0.01 and 0.05 in either sex are in red; and the remaining SNPs are in blue. The dashed line indicates the reference main diagonal line.

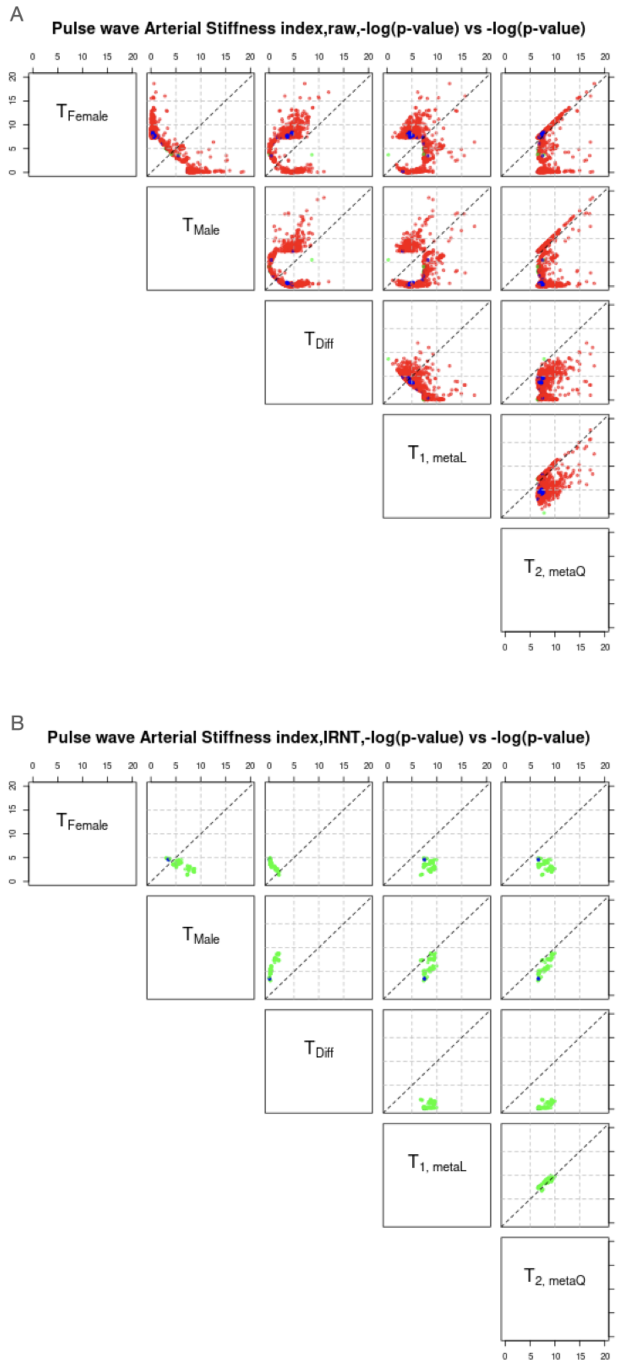

Fig vi. Pairwise scatter plots of the genome-wide significant $(p < 5 \times 10^{-8})$ **SNPs for Pulse wave Arterial Stiffness index GWAS with raw (A) and inverse normal transformed (B) phenotypes detected by any of the five testing methods considered.**

The five association methods include T_{Female} (Female-only analysis), T_{Male} (Male-only analysis), T_{Diff} (SNP-sex interaction-only test), $T_{1,metaL}$ (the traditional sex-combined meta-analysis), and $T_{2,metaQ}$ (the omnibus meta-analysis); see Table 2 for method details. The sex-stratified GWAS summary statistics come from the Neale lab's UK Biobank GWAS round 2, which included a cohort of up to 361,194 participants (118,469 in Pulse wave Arterial Stiffness index GWAS, 63,145 females and 55,324 males). Axes depict $-\log_{10} p$-values for each pair of tests. SNPs are color-coded as follows: SNPs with sex-specific MAF > 0.1 in both sexes are in green; SNPs with sex-specific MAF between 0.01 and 0.05 in either sex are in red; and the remaining SNPs are in blue. The dashed line indicates the reference main diagonal line.

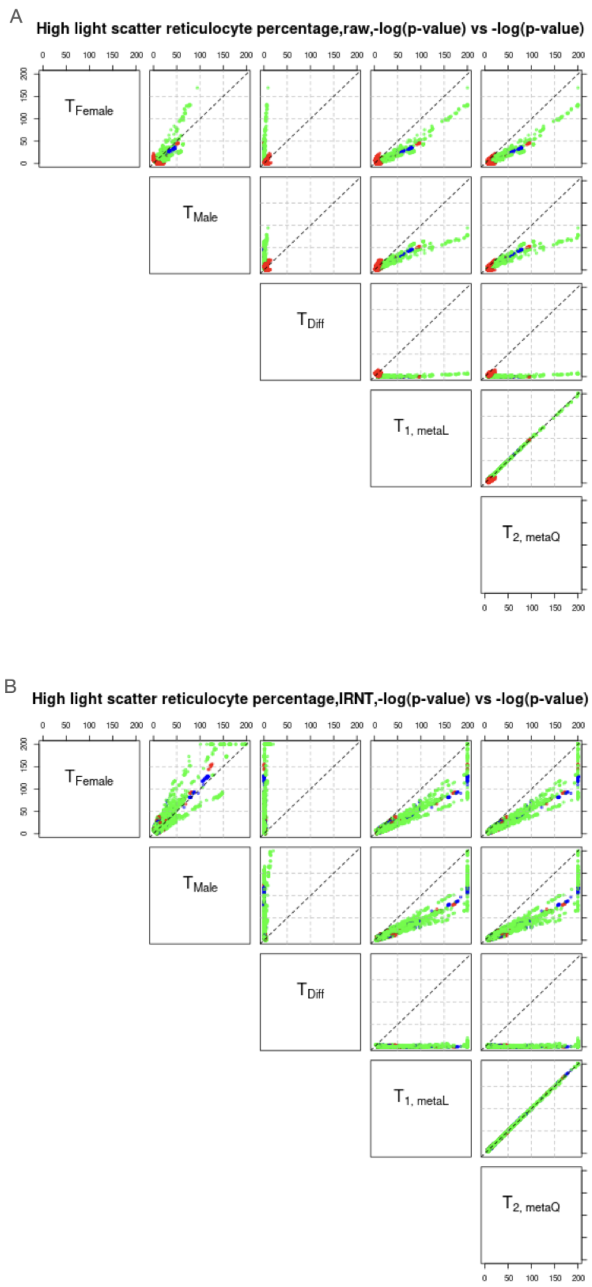

Fig vii. Pairwise scatter plots of the genome-wide significant $(p < 5 \times 10^{-8})$ **SNPs for High light scatter reticulocyte GWAS with raw (A) and inverse normal transformed (B) phenotypes detected by any of the five testing methods considered.**

The five association methods include T_{Female} (Female-only analysis), T_{Male} (Male-only analysis), T_{Diff} (SNP-sex interaction-only test), $T_{1,metaL}$ (the traditional sex-combined meta-analysis), and $T_{2,metaQ}$ (the omnibus meta-analysis); see Table 2 for method details. The sex-stratified GWAS summary statistics come from the Neale lab's UK Biobank GWAS round 2, which included a cohort of up to 361,194 participants (344,729 in High light scatter reticulocyte GWAS, 184,923 females and 159,806 males). Axes depict $-\log_{10} p$-values for each pair of tests. SNPs are color-coded as follows: SNPs with sex-specific MAF > 0.1 in both sexes are in green; SNPs with sex-specific MAF between 0.01 and 0.05 in either sex are in red; and the remaining SNPs are in blue. The dashed line indicates the reference main diagonal line.

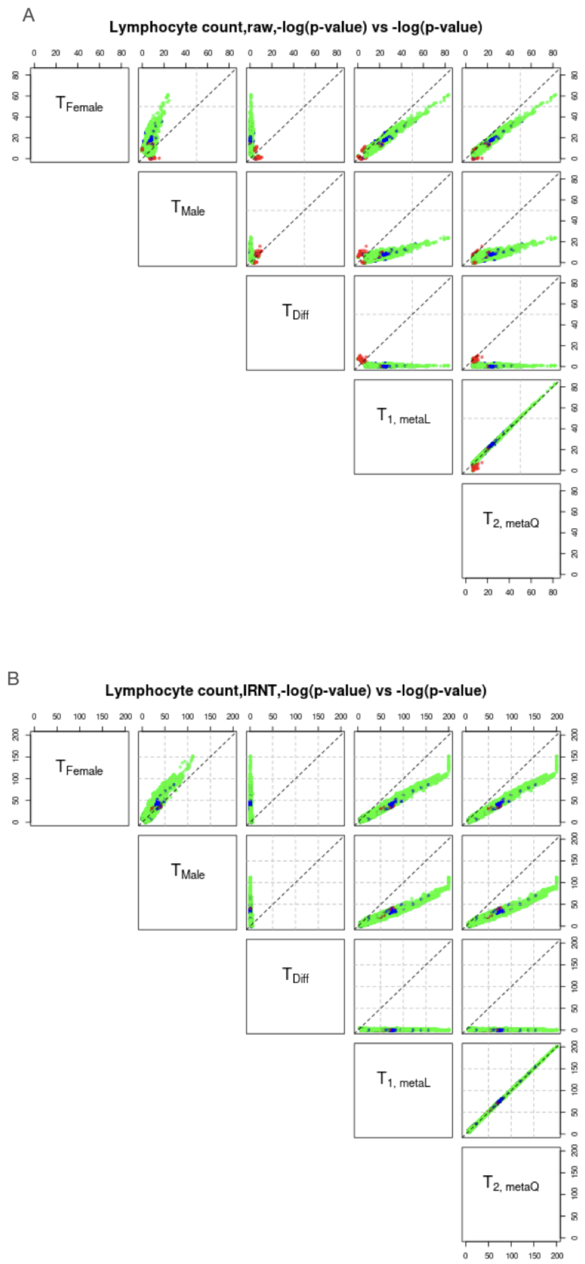

Fig viii. Pairwise scatter plots of the genome-wide significant ($p < 5 \times 10^{-8}$) SNPs for Lymphocyte count GWAS with raw (A) and inverse normal transformed (B) phenotypes detected by any of the five testing methods considered.

The five association methods include T_{Female} (Female-only analysis), T_{Male} (Male-only analysis), T_{Diff} (SNP-sex interaction-only test), $T_{1,metaL}$ (the traditional sex-combined meta-analysis), and $T_{2,metaQ}$ (the omnibus meta-analysis); see Table 2 for method details. The sex-stratified GWAS summary statistics come from the Neale lab's UK Biobank GWAS round 2, which included a cohort of up to 361,194 participants (349,856 in Lymphocyte count GWAS, 187,758 females and 162,098 males). Axes depict $-\log_{10} p$-values for each pair of tests. SNPs are color-coded as follows: SNPs with sex-specific MAF > 0.1 in both sexes are in green; SNPs with sex-specific MAF between 0.01 and 0.05 in either sex are in red; and the remaining SNPs are in blue. The dashed line indicates the reference main diagonal line.

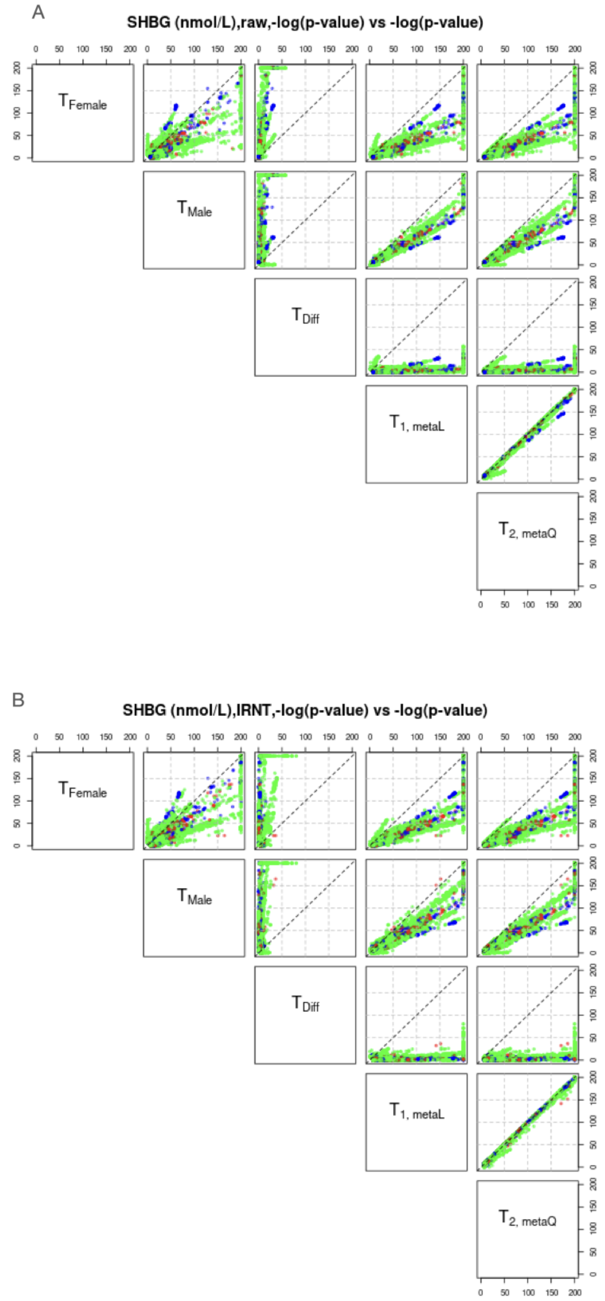

Fig ix. Pairwise scatter plots of the genome-wide significant ($p < 5 \times 10^{-8}$) SNPs for SHBG GWAS with raw (A) and inverse normal transformed (B) phenotypes detected by any of the five testing methods considered.

The five association methods include T_{Female} (Female-only analysis), T_{Male} (Male-only analysis), T_{Diff} (SNP-sex interaction-only test), $T_{1,metaL}$ (the traditional sex-combined meta-analysis), and $T_{2,metaQ}$ (the omnibus meta-analysis); see Table 2 for method details. The sex-stratified GWAS summary statistics come from the Neale lab's UK Biobank GWAS round 2, which included a cohort of up to 361,194 participants (312,215 in SHBG GWAS, 166,235 females and 145,980 males). Axes depict $-\log_{10} p$-values for each pair of tests. SNPs are color-coded as follows: SNPs with sex-specific MAF > 0.1 in both sexes are in green; SNPs with sex-specific MAF between 0.01 and 0.05 in either sex are in red; and the remaining SNPs are in blue. The dashed line indicates the reference main diagonal line.

References

1. McCaw ZR, Lane JM, Saxena R, Redline S, Lin X. Operating characteristics of the rank-based inverse normal transformation for quantitative trait analysis in genome-wide association studies. Biometrics. 2020;76(4):1262–1272. doi:10.1111/biom.13214.

2. Palmer DS, Zhou W, Abbott L, Wigdor EM, Baya N, Churchhouse C, et al. Analysis of genetic dominance in the UK Biobank. Science. 2023;379(6639):1341–1348. doi:10.1126/science.abn8455.
