# Supplementary material for: Better together against genetic heterogeneity: A sex-combined joint main and interaction analysis of 290 quantitative traits in the UK Biobank
Source: PLoS Genet. 2024 Apr 24;20(4):e1011221. doi: 10.1371/journal.pgen.1011221 (PMC11073786; doi:10.1371/journal.pgen.1011221)
Supplement: S5 Appendix — (PDF) [file pgen.1011221.s005.pdf]

S5 Analysis check on MAF

We observed a noticeable enrichment of significant SNPs with MAF at approximately 0.225 (top-left panel in Fig i; appeared consistently among all the five tests). Chromosome-stratified plots revealed that this enrichment was primarily associated with the 900-kb common inversion found within the expansive region of conserved linkage disequilibrium (LD) on chromosome 17q21.3 [1].

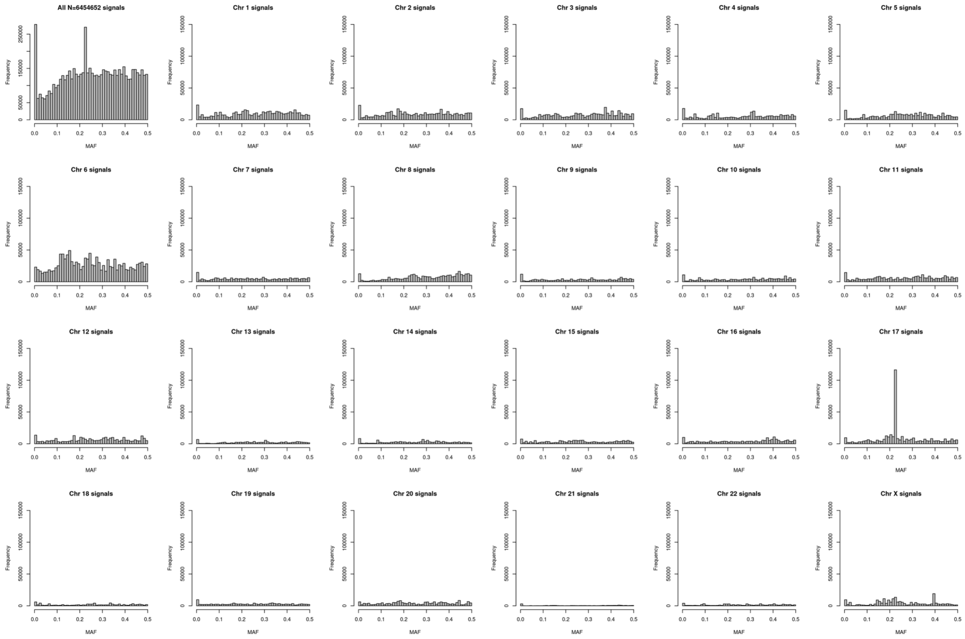

Fig i. Sanity check by examining the MAF distributions of the 6,454,652 SNP-phenotype associations that were genome-wide significant in any of the five tests we considered across 290 traits.

References

1. Stefansson H, Helgason A, Thorleifsson G, Steinthorsdottir V, Masson G, Barnard J, et al. A common inversion under selection in Europeans. Nature Genetics. 2005;37(2):129–137. doi:10.1038/ng1508.
