# Supplementary material for: Better together against genetic heterogeneity: A sex-combined joint main and interaction analysis of 290 quantitative traits in the UK Biobank
Source: PLoS Genet. 2024 Apr 24;20(4):e1011221. doi: 10.1371/journal.pgen.1011221 (PMC11073786; doi:10.1371/journal.pgen.1011221)
Supplement: S6 Appendix — (PDF) [file pgen.1011221.s006.pdf]

S6 SNPs with opposite significant effects between sexes for urate on chr4

Noticeably, in the comparison between T_{Male} and T_{Diff} in Figure 1 (both panel A and B), there is a cluster of SNPs that are genome-wide significant in both T_{Male} and T_{Diff}, but the $-\log_{10} p$-values are slightly higher in T_{Male}, ranging from 75 to 125, compared to T_{Diff} where they range from 55 to 80. These SNPs are associated with urate and also exhibit $-log_{10}p$-values exceeding 200 in T_{Female}, clustering near the top border of sub-figures comparing T_{Female} and T_{Diff} in Figure 1. These SNPs are located on chr4:8000000 $\sim$ 10500000 (Fig iA). Interestingly, this region contains 20 SNPs with genome-wide significant effect with urate in both females and males but with opposing effect directions, which represents the only instance in our analysis across all 290 phenotypes we analyzed (Fig iB; Fig ii-Fig vi).

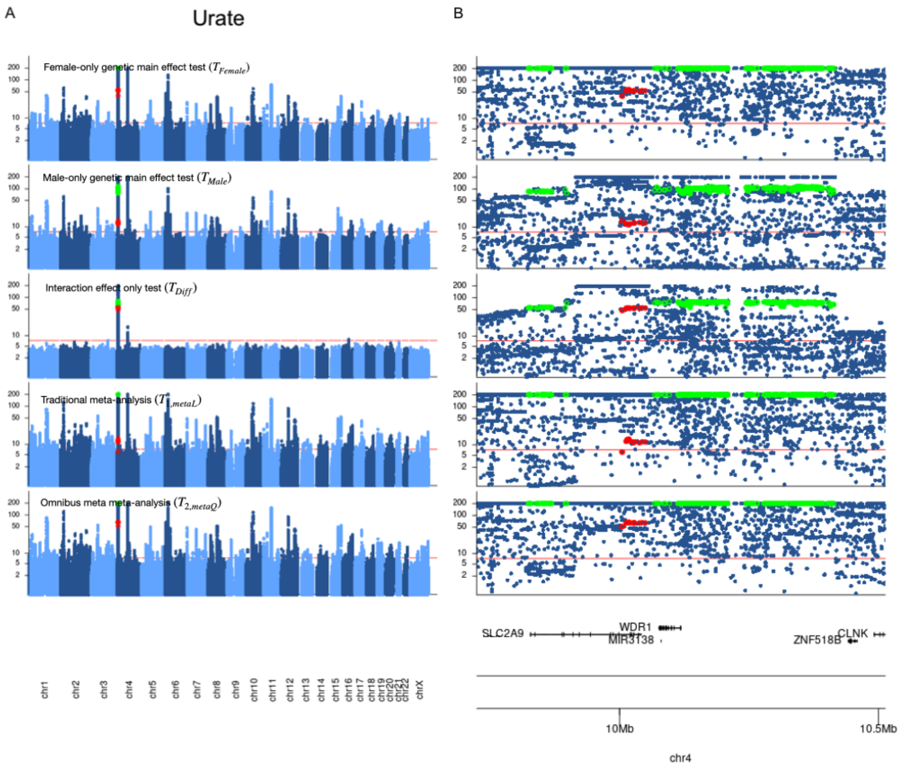

Fig i. Stacked Manhattan plots of genetic association for urate (A) and zoomed region on chr4:8000000 $\sim$ 10500000 (B). The $-\log_{10} p$-values are shown for the five association methods, including T_{Female} (Female-only analysis), T_{Male} (Male-only analysis), T_{Diff} (SNP-sex interaction-only test), $T_{1,metaL}$ (the traditional sex-combined meta-analysis), and $T_{2,metaQ}$ (the omnibus meta-analysis); see Table 1 for method details. The sex-stratified GWAS summary statistics come from the Neale lab's UK Biobank GWAS round 2, which included a cohort of up to 361,194 participants (343,836 in urate GWAS, 184,755 females and 159,081 males). SNPs with $-\log_{10} p$-values of T_{Diff} and T_{Male} ranging from 55 to 80 and 75 to 125 respectively are highlighted in green (S6 Appendix). SNPs with genome-wide significant effect in both females and males but with opposing effect directions are highlighted in red. The red horizontal lines indicate the genome-wide significant threshold of 5×10^{-8} on the $-log_{10}$ scale.

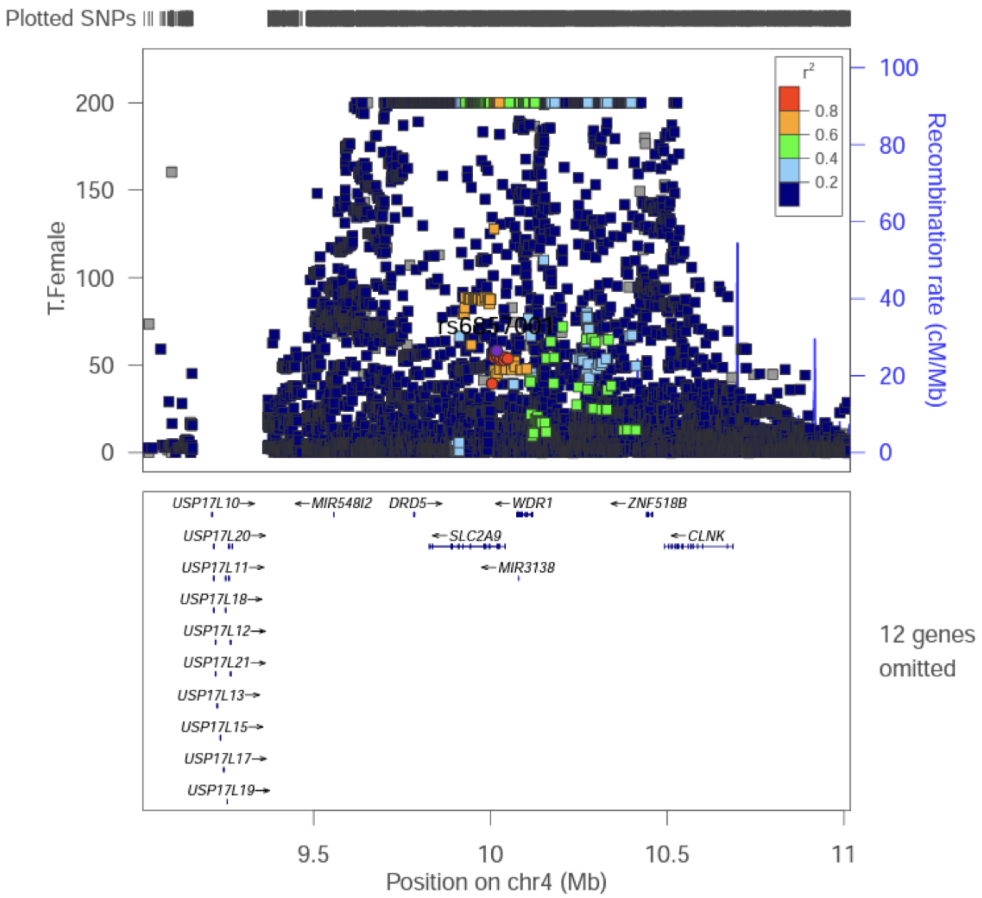

Fig ii. Locuszoom plot of T_{Female} for the Chr4: 9018080 ∼ 11018080 region near SLC2A9 containing 19 SNPs with genome-wide significant effects on urate in both female and male analysis with opposite directions. The SNP, rs6857001, which has the lowest $T_{2,metaQ}$ p-value, is highlighted in purple. Other colors represent the linkage disequilibrium (LD) between annotated SNPs and rs6857001. Nineteen SNPs, demonstrating genome-significant effects in both females and males, are depicted with circles, with rectangles being used otherwise.

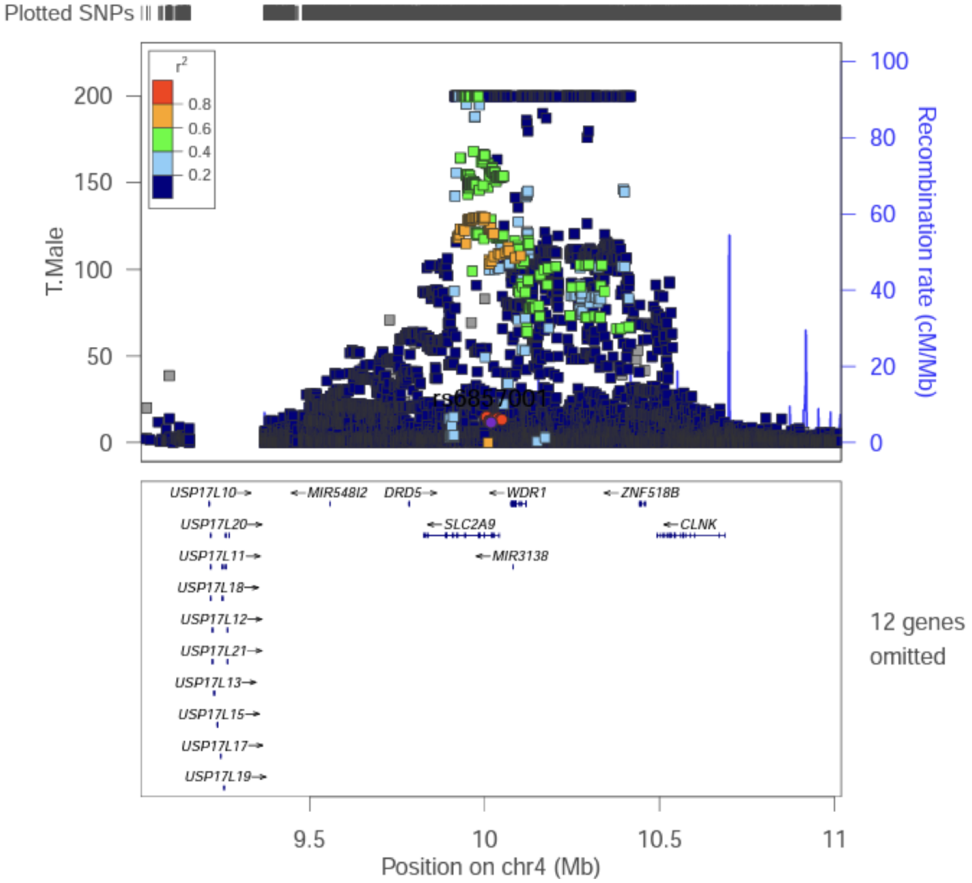

Fig iii. Locuszoom plot of T_{Male} for the Chr4: 9018080 ∼ 11018080 region near SLC2A9 containing 19 SNPs with genome-wide significant effects on urate in both female and male analysis with opposite directions. The SNP, rs6857001, which has the lowest $T_{2,metaQ}$ p-value, is highlighted in purple. Other colors represent the linkage disequilibrium (LD) between annotated SNPs and rs6857001. Nineteen SNPs, demonstrating genome-significant effects in both females and males, are depicted with circles, with rectangles being used otherwise.

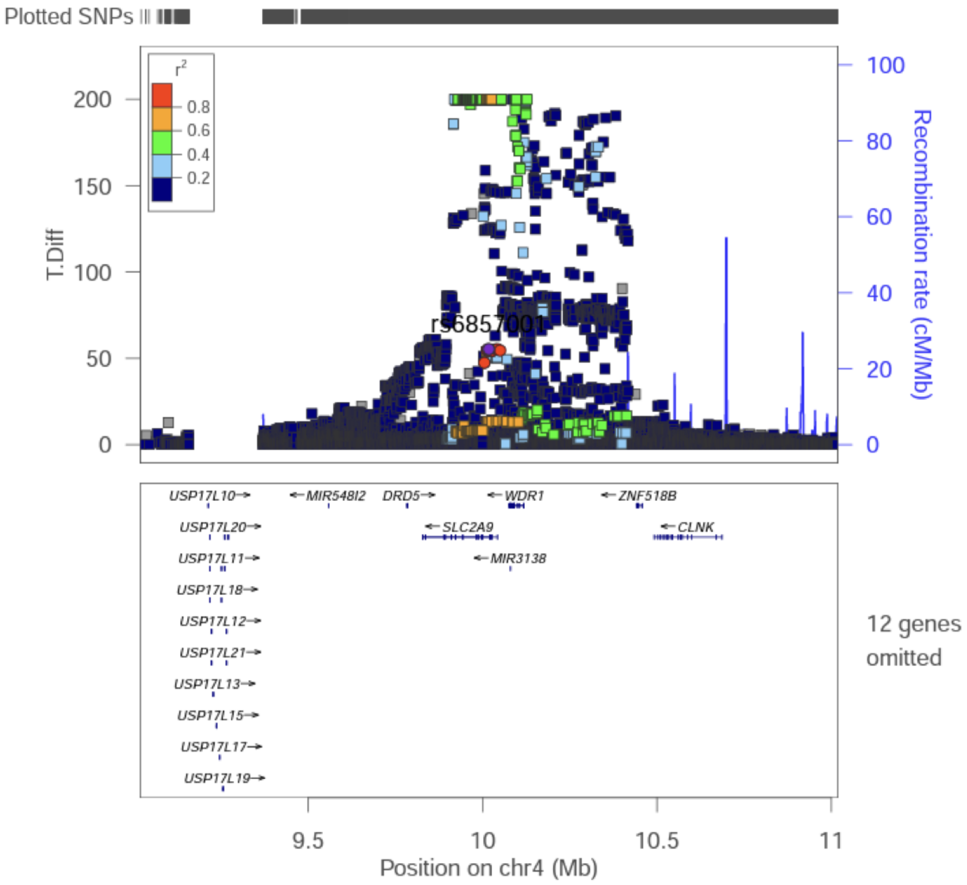

Fig iv. Locuszoom plot of T_{Diff} for the Chr4: 9018080 $\sim$ 11018080 region near SLC2A9 containing 19 SNPs with genome-wide significant effects on urate in both female and male analysis with opposite directions. The SNP, rs6857001, which has the lowest $T_{2,metaQ}$ p-value, is highlighted in purple. Other colors represent the linkage disequilibrium (LD) between annotated SNPs and rs6857001. Nineteen SNPs, demonstrating genome-significant effects in both females and males, are depicted with circles, with rectangles being used otherwise.

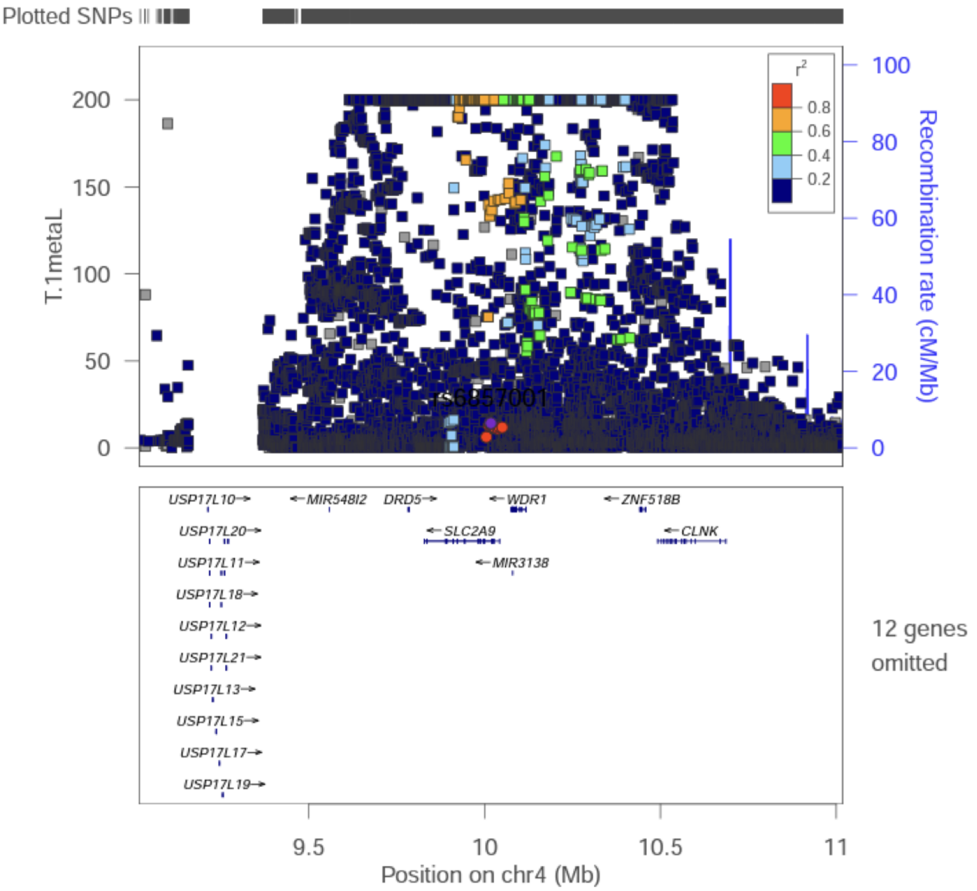

Fig v. Locuszoom plot of $T_{1,metaL}$ for the Chr4: 9018080 $\sim$ 11018080 region near SLC2A9 containing 19 SNPs with genome-wide significant effects on urate in both female and male analysis with opposite directions. The SNP, rs6857001, which has the lowest $T_{2,metaQ}$ p-value, is highlighted in purple. Other colors represent the linkage disequilibrium (LD) between annotated SNPs and rs6857001. Nineteen SNPs, demonstrating genome-significant effects in both females and males, are depicted with circles, with rectangles being used otherwise.

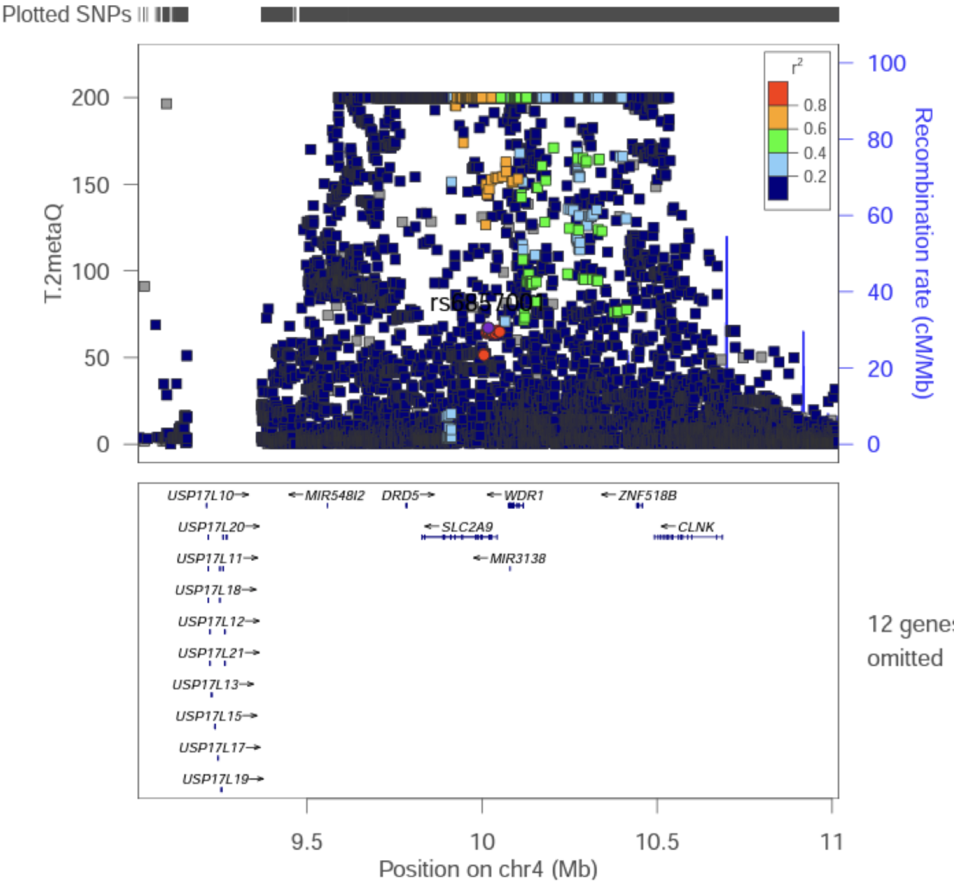

Fig vi. Locuszoom plot of $T_{2,metaQ}$ for the Chr4: 9018080 $\sim$ 11018080 region near SLC2A9 containing 19 SNPs with genome-wide significant effects on urate in both female and male analysis with opposite directions. The SNP, rs6857001, which has the lowest $T_{2,metaQ}$ p-value, is highlighted in purple. Other colors represent the linkage disequilibrium (LD) between annotated SNPs and rs6857001. Nineteen SNPs, demonstrating genome-significant effects in both females and males, are depicted with circles, with rectangles being used otherwise.
