# Supplementary material for: Better together against genetic heterogeneity: A sex-combined joint main and interaction analysis of 290 quantitative traits in the UK Biobank
Source: PLoS Genet. 2024 Apr 24;20(4):e1011221. doi: 10.1371/journal.pgen.1011221 (PMC11073786; doi:10.1371/journal.pgen.1011221)
Supplement: S7 Appendix — (PDF) [file pgen.1011221.s007.pdf]

S7 Analytical ranges for p-values of $\boldsymbol{T_{1,metaL}}$ and $\boldsymbol{T_{2,metaQ}}$ in Figure 2

Here we show the following statements regarding the bounds for p-value observed in Figure 2:

a. For SNPs identified by $T_{2,metaQ}$ but missed by $T_{1,metaL}$ (Fig 2A), $-\log_{10} p_{1,metaL} \in (0, 7.3]$, $-\log_{10} p_{2,metaQ} \in (7.3, \infty)$;

b. For SNPs identified by $T_{1,metaL}$ but missed by $T_{2,metaQ}$ (Fig 2B), $-\log_{10} p_{1,metaL} \in (7.30, 8.17]$, $-\log_{10} p_{2,metaQ} \in [6.45, 7.30)$.

Proof: Given T_F, T_M, we have

$$\begin{aligned} T_{1,metaL}^2 &= \left(\sqrt{w_f} T_F + \sqrt{1-w_f} T_M\right)^2 \overset{H_0}{\sim} \chi_1^2, \\ T_{2,metaQ} &= T_F^2 + T_M^2 \overset{H_0}{\sim} \chi_2^2, \end{aligned}$$

where $w_F = \frac{1/\hat{v}_F^2}{1/\hat{v}_F^2 + 1/\hat{v}_M^2}$. By Cauchy–Schwarz inequality,

$$0 \leq T_{1,metaL}^2 \leq [w_F + (1 - w_F)](T_F^2 + T_M^2) = T_{2,metaQ}, \tag{3}$$

with equality holds only when $\hat{\beta}_F = \hat{\beta}_M$.

For SNPs in a., we have

$$\begin{aligned} P(\chi_2^2 > t_{2,metaQ}) < 5 \times 10^{-8} &\Rightarrow t_{2,metaQ} > 33.62 \\ P(\chi_1^2 > t_{1,metaL}^2) > 5 \times 10^{-8} &\Rightarrow t_{1,metaQ}^2 < 29.72, \end{aligned}$$

all satisfies 3 so no upper limits for $-\log_{10} T_{2,metaQ}$ and $-\log_{10} T_{1,metaL}$ could go as low 0.

For SNPs in b., we have

$$\begin{aligned} P(\chi_2^2 > t_{2,metaQ}) > 5 \times 10^{-8} &\Rightarrow t_{2,metaQ} < 33.62 \\ P(\chi_1^2 > t_{1,metaL}^2) < 5 \times 10^{-8} &\Rightarrow t_{1,metaQ}^2 > 29.72 \end{aligned}$$

combine with 3,

$$\begin{aligned} 29.72 \leq t_{2,metaQ} < 33.62 &\Rightarrow log_{10} p_{2,metaQ} \in [6.45, 7.30) \\ 29.72 < t_{1,metaL}^2 \leq 33.62 &\Rightarrow log_{10} p_{1,metaL} \in (7.30, 8.17], \end{aligned}$$

where the closed bounds were reached when genetic effects are the same between male and female. Therefore this set reflects the small cost of $T_{2,metaQ}$ in the absence effect difference due to additional 1 degree of freedom.
