# Supplementary figures and images for: Better together against genetic heterogeneity: A sex-combined joint main and interaction analysis of 290 quantitative traits in the UK Biobank
Source: PLoS Genet. 2024 Apr 24;20(4):e1011221. doi: 10.1371/journal.pgen.1011221 (PMC11073786; doi:10.1371/journal.pgen.1011221)

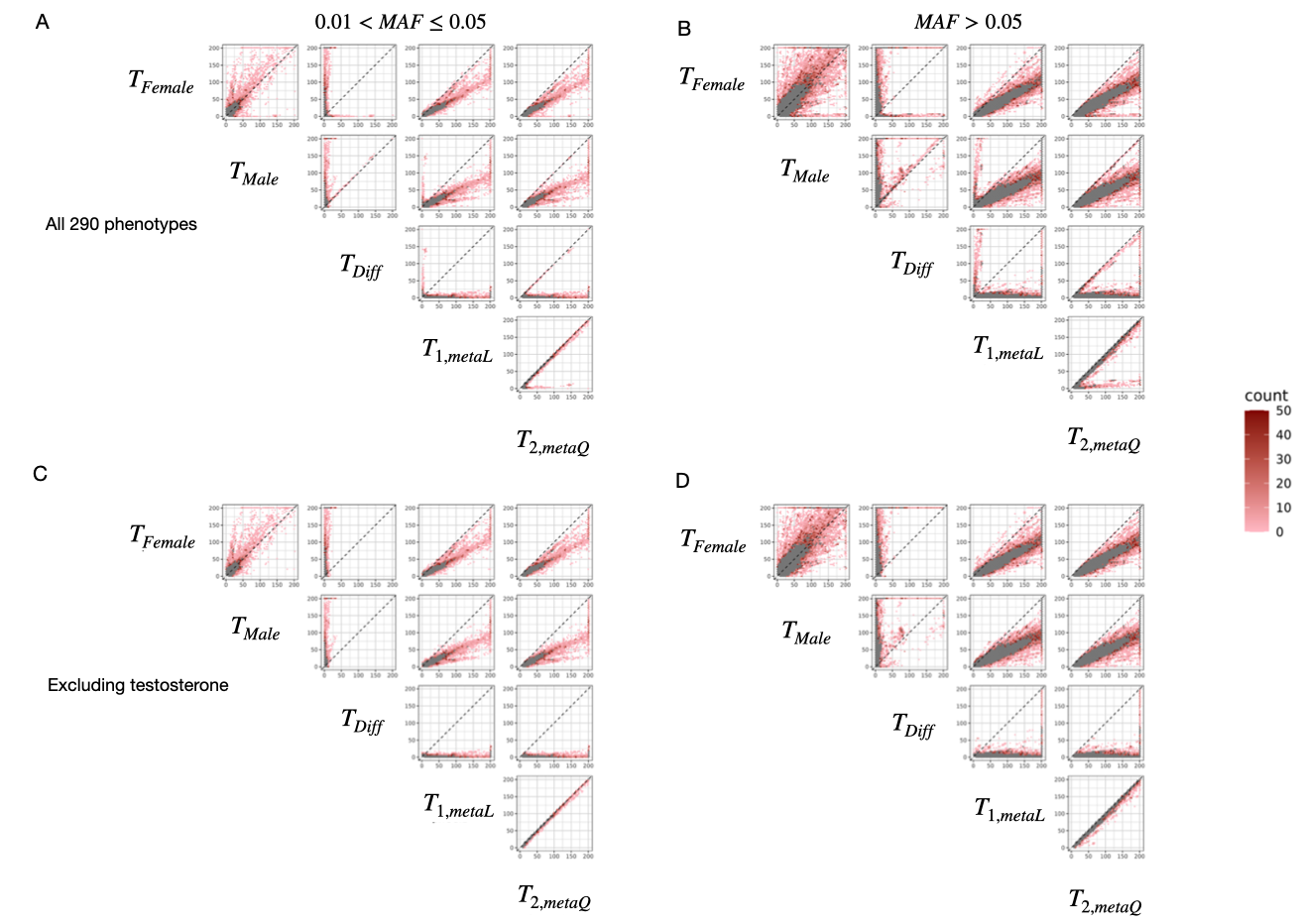

Supplement: S1 Fig — (A) includes SNPs with either sex-stratified MAF ≤ 0.05, and (B) includes SNPs with MAF > 0.05 in both sex groups, across all 290 traits analyzed and after excluding the testosterone (C and D). The five association methods include TFemale (Female-only analysis), TMale (Male-only analysis), TDiff (SNP-sex interaction-only test), T1,metaL (the traditional sex-combined meta-analysis), and T2,metaQ (the omnibus meta-analysis); see Table 1 for method details. The sex-stratified GWAS summary statistics come from the Neale lab’s UK Biobank GWAS round 2, which included a cohort of up to 361,194 participants (312,102 in testosterone GWAS, 154,364 females and 157,738 males). Axes depict −log10 p-values for each pair of tests, and each hexagon’s color corresponds to the count of associations falling within the −log10 p-value range defined by that region. The −log10 p maximum was truncated at 200 to improve visualization. The dashed line indicates the reference main diagonal reference line. (TIF) [file pgen.1011221.s009.tif]

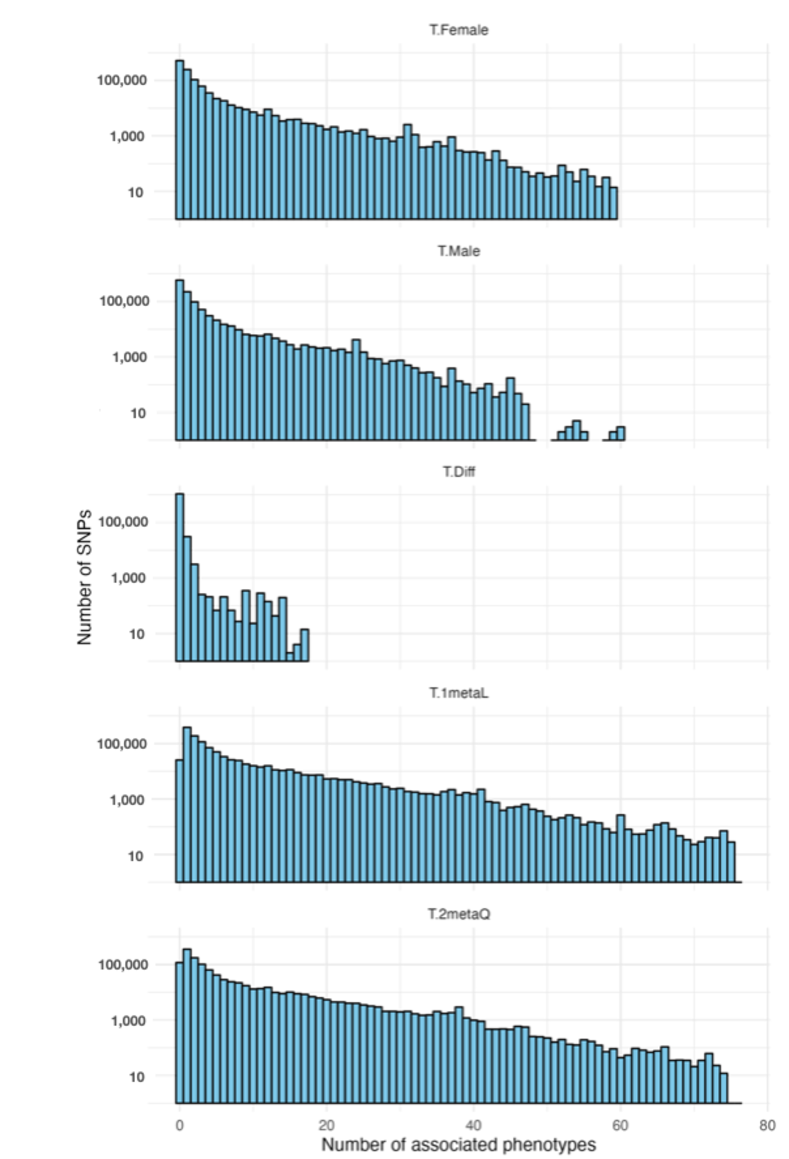

Supplement: S2 Fig — 1,113,865 SNPs are associated with one or more traits identified by any of the five association testing methods: TFemale (Female-only analysis), TMale (Male-only analysis), TDiff (SNP-sex interaction-only test), T1,metaL (the traditional sex-combined meta-analysis), and T2,metaQ (the omnibus meta-analysis). The histograms are based on Nj,m=∑t=1290I(pj,t,m<5×10^-8^), where I(⋅) is an indicator function, pj,t, m is the association p-value between SNP j and trait t by method m. The y-axis ticks are on the log10 scale for ease of visualization. (TIF) [file pgen.1011221.s010.tif]

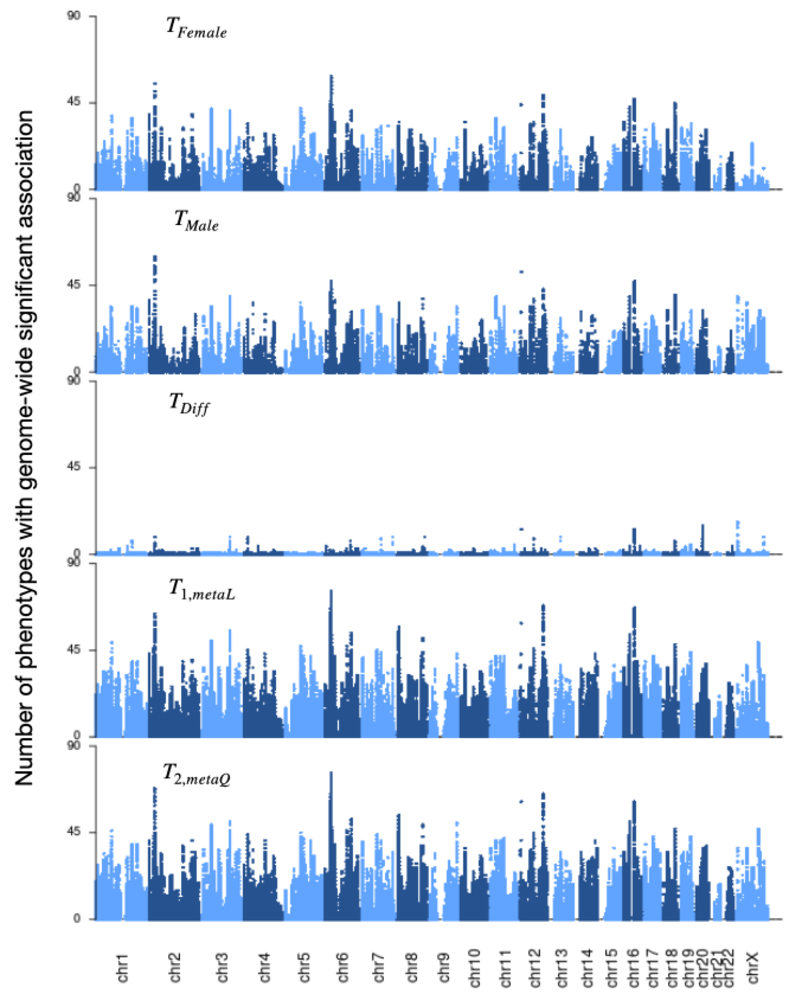

Supplement: S3 Fig — The 1,113,865 SNPs are associated with one or more traits identified by any of the five association testing methods: TFemale (Female-only analysis), TMale (Male-only analysis), TDiff (SNP-sex interaction-only test), T1,metaL (the traditional sex-combined meta-analysis), and T2,metaQ (the omnibus meta-analysis). The plots are based on Nj,m=∑t=1290I(pj,t,m<5×10^-8^), where I(⋅) is an indicator function, pj,t, m is the association p-value between SNP j and trait t by method m. (TIF) [file pgen.1011221.s011.tif]

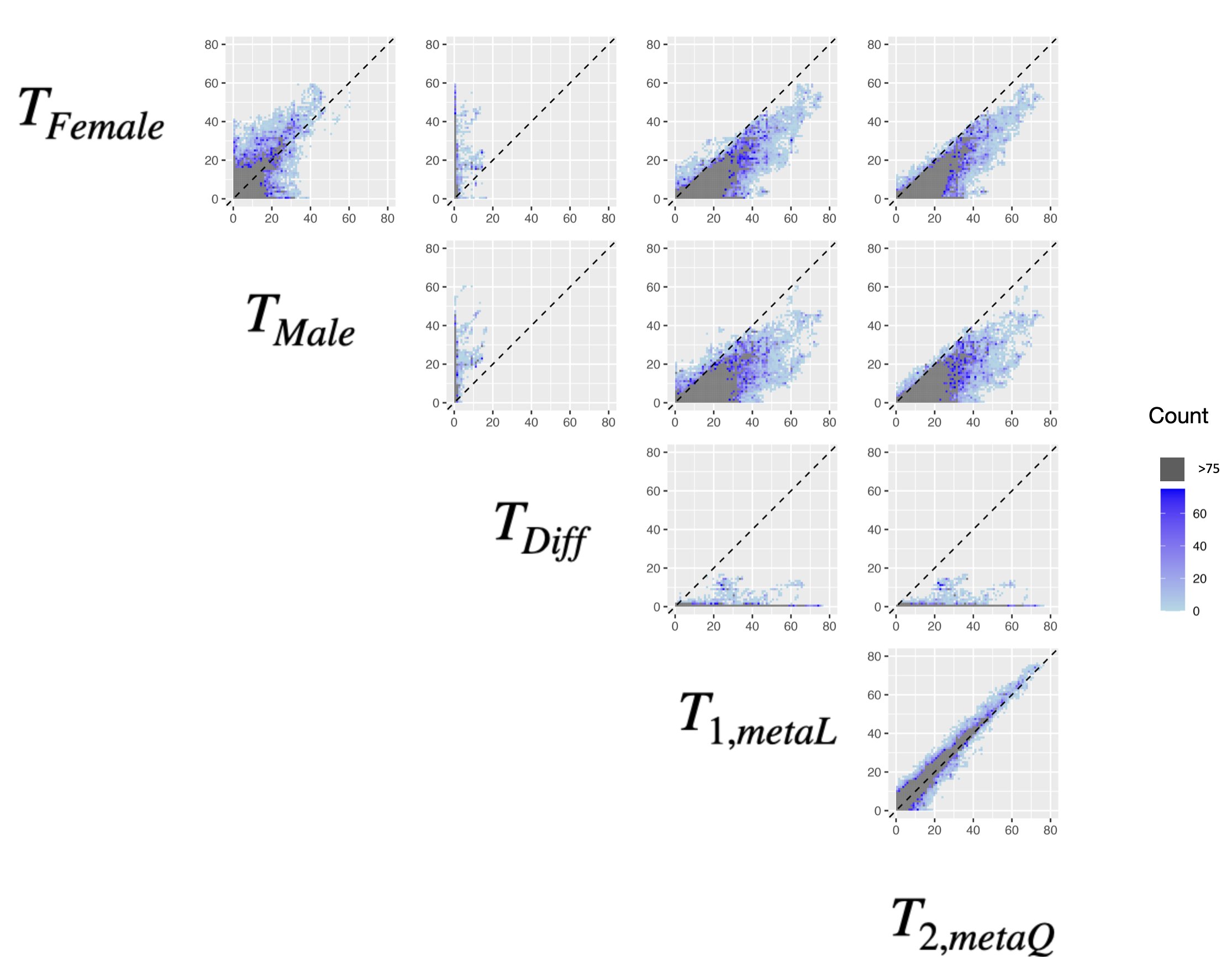

Supplement: S4 Fig — The 1,113,865 SNPs are the SNPs associated with one or more traits identified by any of the five association testing methods: TFemale (Female-only analysis), TMale (Male-only analysis), TDiff (SNP-sex interaction-only test), T1,metaL (the traditional sex-combined meta-analysis), and T2,metaQ (the omnibus meta-analysis). The plots are based on Nj,m=∑t=1290I(pj,t,m<5×10^-8^), where I(⋅) is an indicator function, pj,t, m is the association p-value between SNP j and trait t by method m. (TIF) [file pgen.1011221.s012.tif]

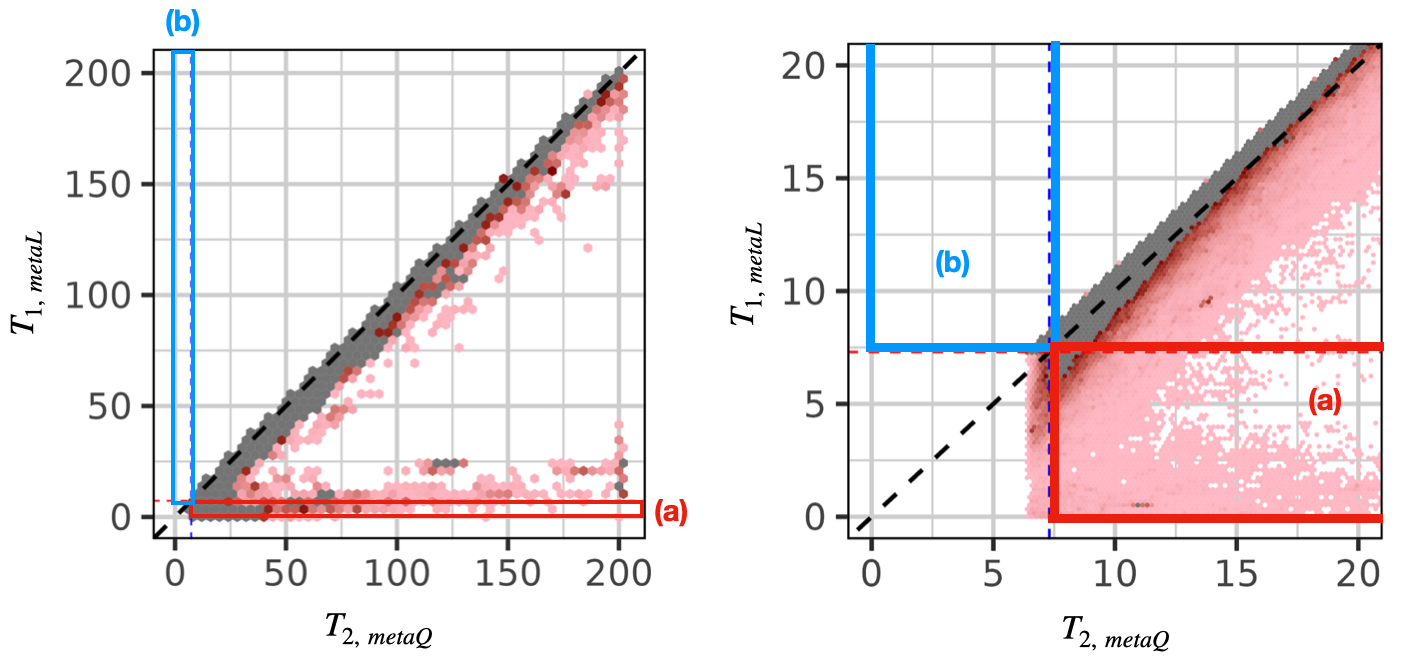

Supplement: S5 Fig — In the red-highlighted area (a), there are 179, 718 SNP-trait associations which were identified by T2,metaQ but missed by T1,metaL, and in the blue-highlighted area (b), there are 756, 316 SNP-trait associations which were identified by T1,metaL but missed by T2,metaQ. (TIF) [file pgen.1011221.s013.tif]

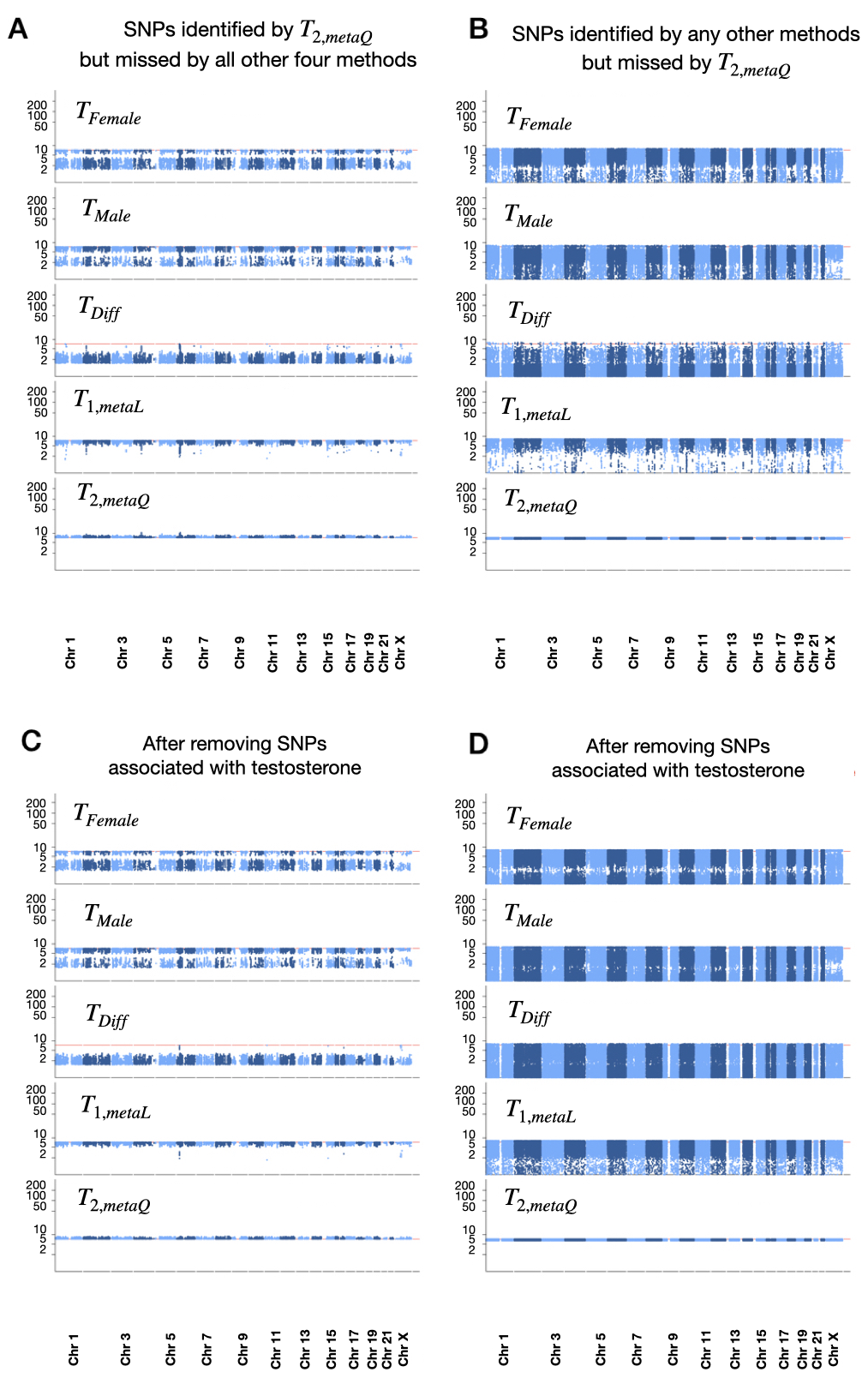

Supplement: S6 Fig — (A) 64,934 SNP-phenotype associations identified by T2,metaQ but missed by all other four methods across all the 290 traits, (B) 800,183 SNP-phenotype associations missed by T2,metaQ but identified by any other four methods across all the 290 traits, (C) 63,647 SNP-phenotype associations identified by T2,metaQ but missed by all other four methods after removing the testosterone, and (D) 797,585 SNP-phenotype associations missed by T2,metaQ but identified by any other four methods after removing the testosterone. The −log10 p-values (with further log10 transformation on y-axis to aid presentation) are shown for the five association methods, including TFemale (Female-only analysis), TMale (Male-only analysis), TDiff (SNP-sex interaction-only test), T1,metaL (the traditional sex-combined meta-analysis), and T2,metaQ (the omnibus meta-analysis); see Table 1 for method details. The sex-stratified GWAS summary statistics come from the Neale lab’s UK Biobank GWAS round 2, which included a cohort of up to 361,194 participants (194,174 females and 167,020 males). The red horizontal lines indicate the genome-wide significant threshold of 5 × 10^−8^ on the −log10 scale. (TIF) [file pgen.1011221.s014.tif]

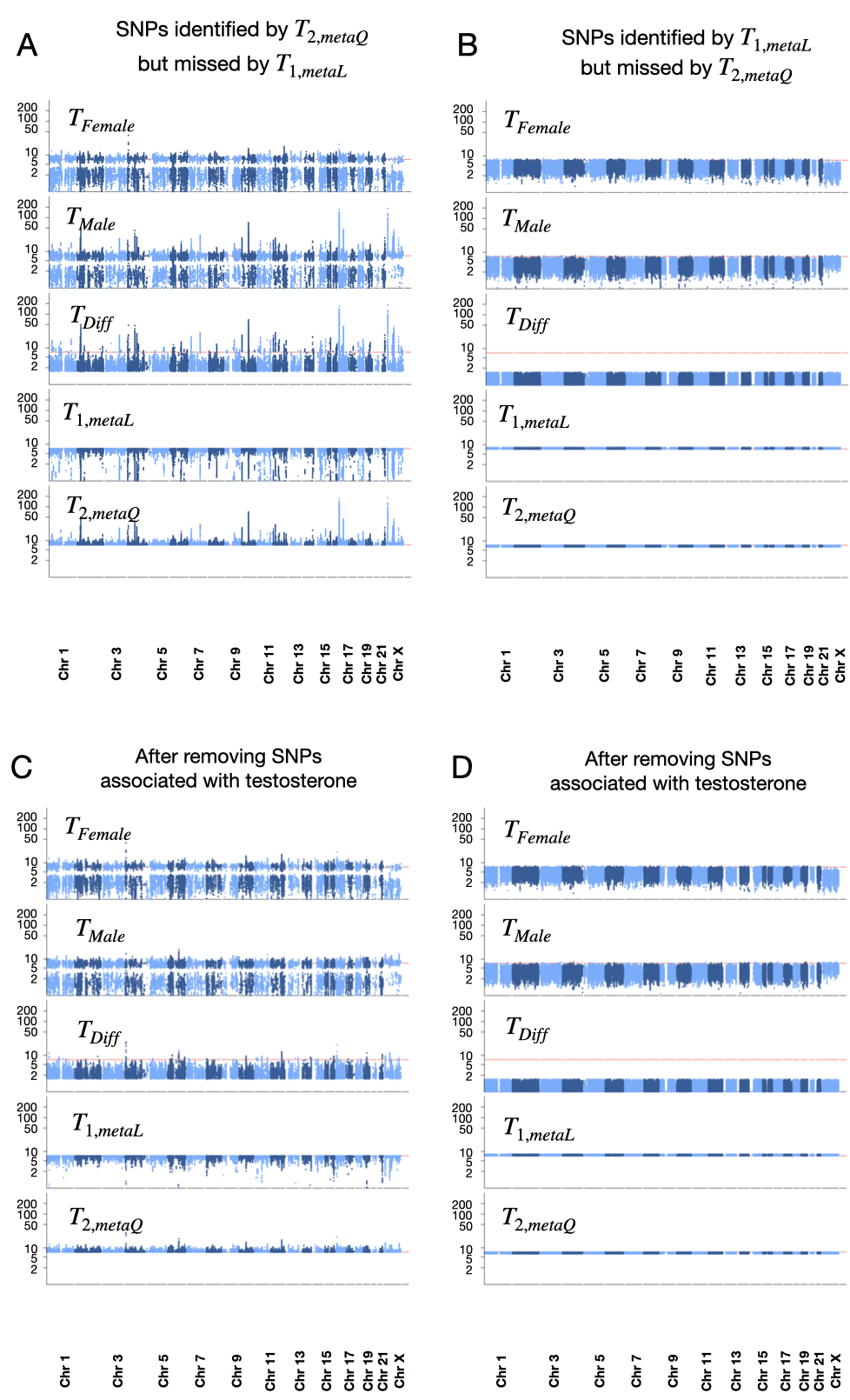

Supplement: S7 Fig — (A) SNPs identified by T2,metaQ but missed by T1,metaL across all the 290 traits, (B) SNPs missed by T2,metaQ but identified by T1,metaL across all the 290 traits, (C) SNPs identified by T2,metaQ but missed by T1,metaL after removing the testosterone, and (D) SNPs missed by T2,metaQ but identified by T1,metaL after removing the testosterone. The −log10 p-values (with further log10 transformation on y-axis to aid presentation) are shown for the five association methods, including TFemale (Female-only analysis), TMale (Male-only analysis), TDiff (SNP-sex interaction-only test), T1,metaL (the traditional sex-combined meta-analysis), and T2,metaQ (the omnibus meta-analysis); see Table 1 for method details. The sex-stratified GWAS summary statistics come from the Neale lab’s UK Biobank GWAS round 2, which included a cohort of up to 361,194 participants (194,174 females and 167,020 males). The red horizontal lines indicate the genome-wide significant threshold of 5 × 10^−8^ on the −log10 scale. (TIF) [file pgen.1011221.s015.tif]

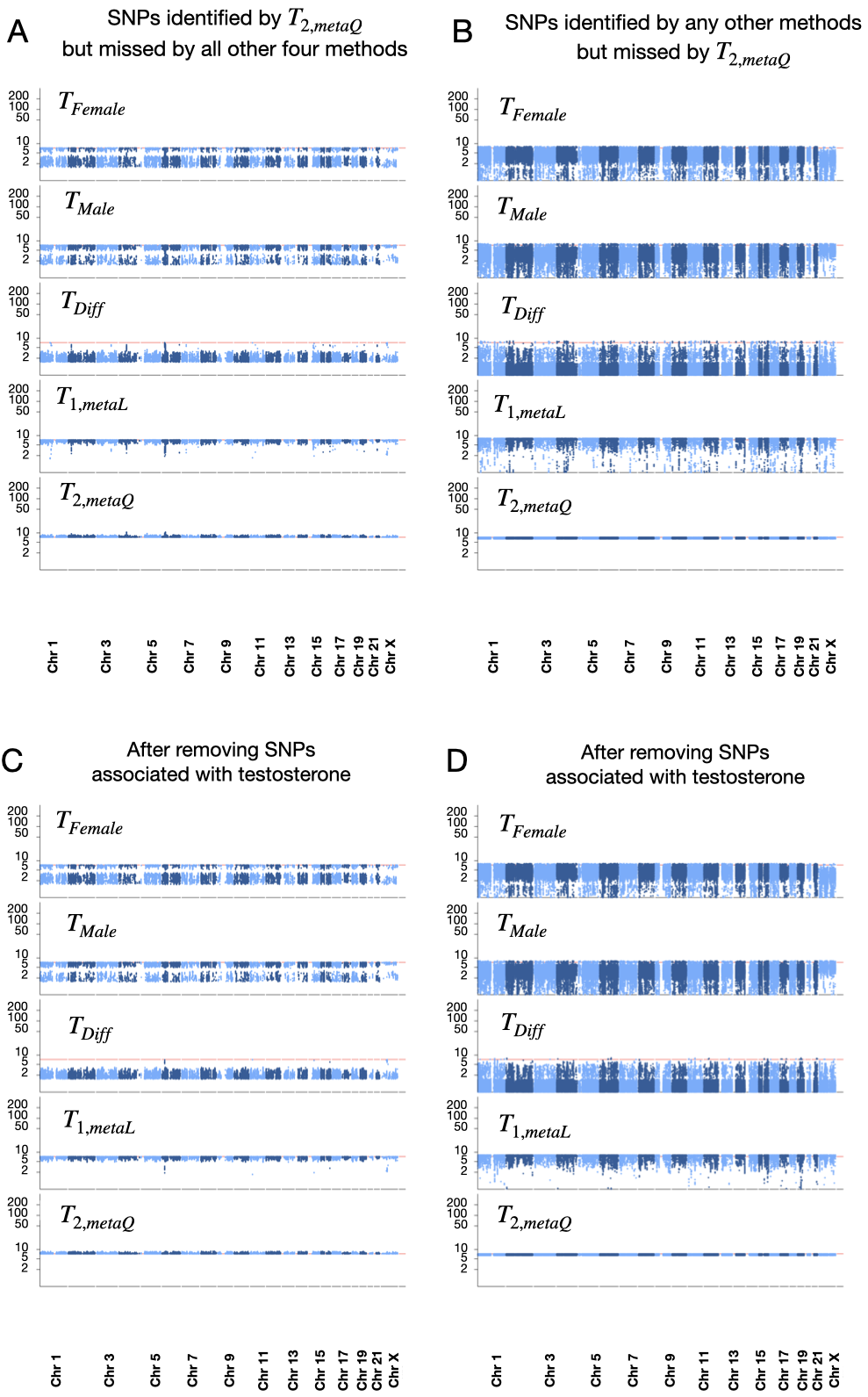

Supplement: S8 Fig — (A) SNPs identified by T2,metaQ but missed by all other four methods across all the 290 traits, (B) SNPs missed by T2,metaQ but identified by any other four methods across all the 290 traits, (C) SNPs identified by T2,metaQ but missed by all other four methods after removing the testosterone, and (D) SNPs missed by T2,metaQ but identified by any other four methods after removing the testosterone. The −log10 p-values (with further log10 transformation on y-axis to aid presentation) are shown for the five association methods, including TFemale (Female-only analysis), TMale (Male-only analysis), TDiff (SNP-sex interaction-only test), T1,metaL (the traditional sex-combined meta-analysis), and T2,metaQ (the omnibus meta-analysis); see Table 1 for method details. The sex-stratified GWAS summary statistics come from the Neale lab’s UK Biobank GWAS round 2, which included a cohort of up to 361,194 participants (194,174 females and 167,020 males). The red horizontal lines indicate the genome-wide significant threshold of 5 × 10^−8^ on the −log10 scale. (TIF) [file pgen.1011221.s016.tif]

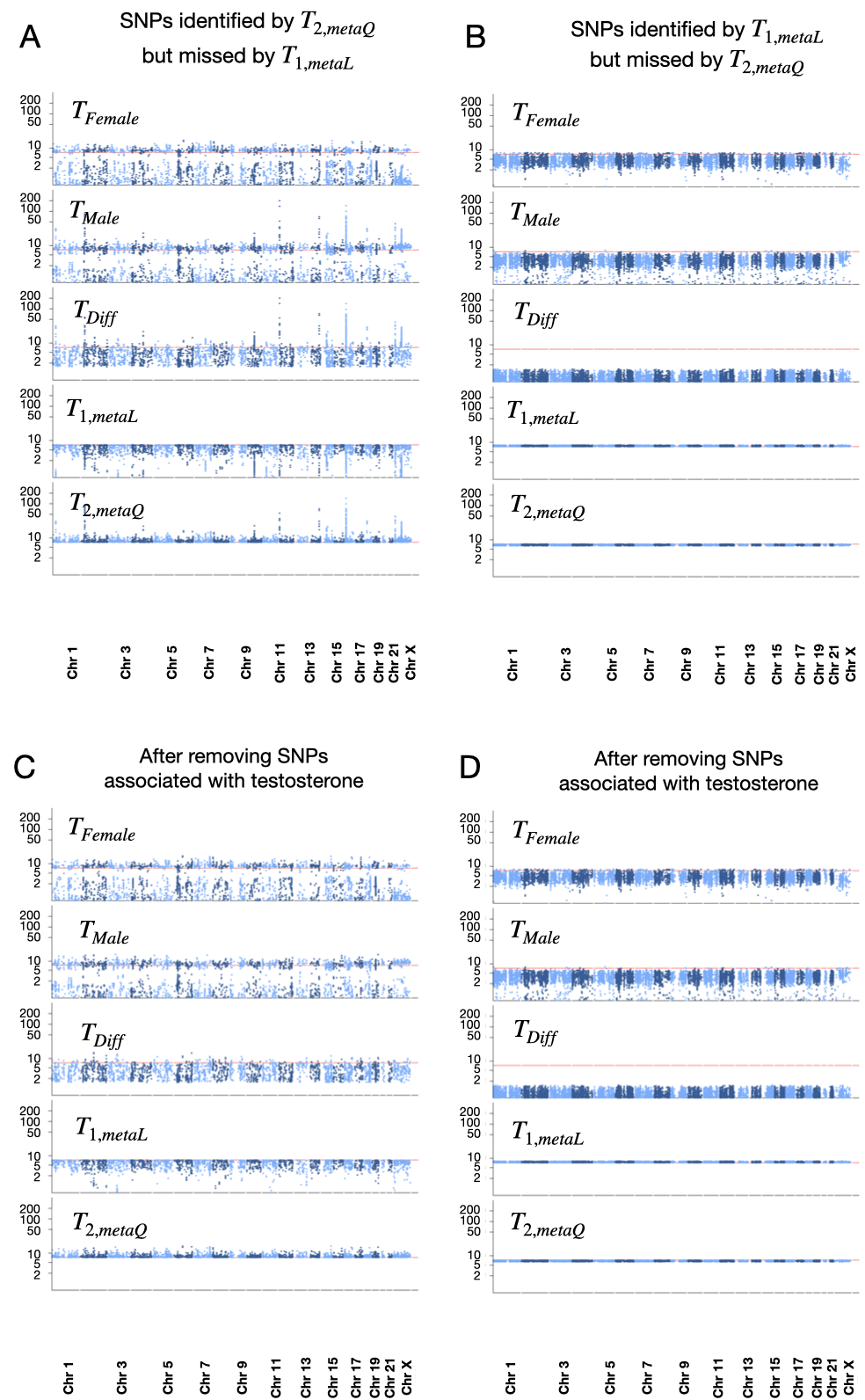

Supplement: S9 Fig — (A) SNPs identified by T2,metaQ but missed by T1,metaL across all the 290 traits, (B) SNPs missed by T2,metaQ but identified by T1,metaL across all the 290 traits, (C) SNPs identified by T2,metaQ but missed by T1,metaL after removing the testosterone, and (D) SNPs missed by T2,metaQ but identified by T1,metaL after removing the testosterone. The −log10 p-values (with further log10 transformation on y-axis to aid presentation) are shown for the five association methods, including TFemale (Female-only analysis), TMale (Male-only analysis), TDiff (SNP-sex interaction-only test), T1,metaL (the traditional sex-combined meta-analysis), and T2,metaQ (the omnibus meta-analysis); see Table 1 for method details. The sex-stratified GWAS summary statistics come from the Neale lab’s UK Biobank GWAS round 2, which included a cohort of up to 361,194 participants (194,174 females and 167,020 males). The red horizontal lines indicate the genome-wide significant threshold of 5 × 10^−8^ on the −log10 scale. (TIF) [file pgen.1011221.s017.tif]

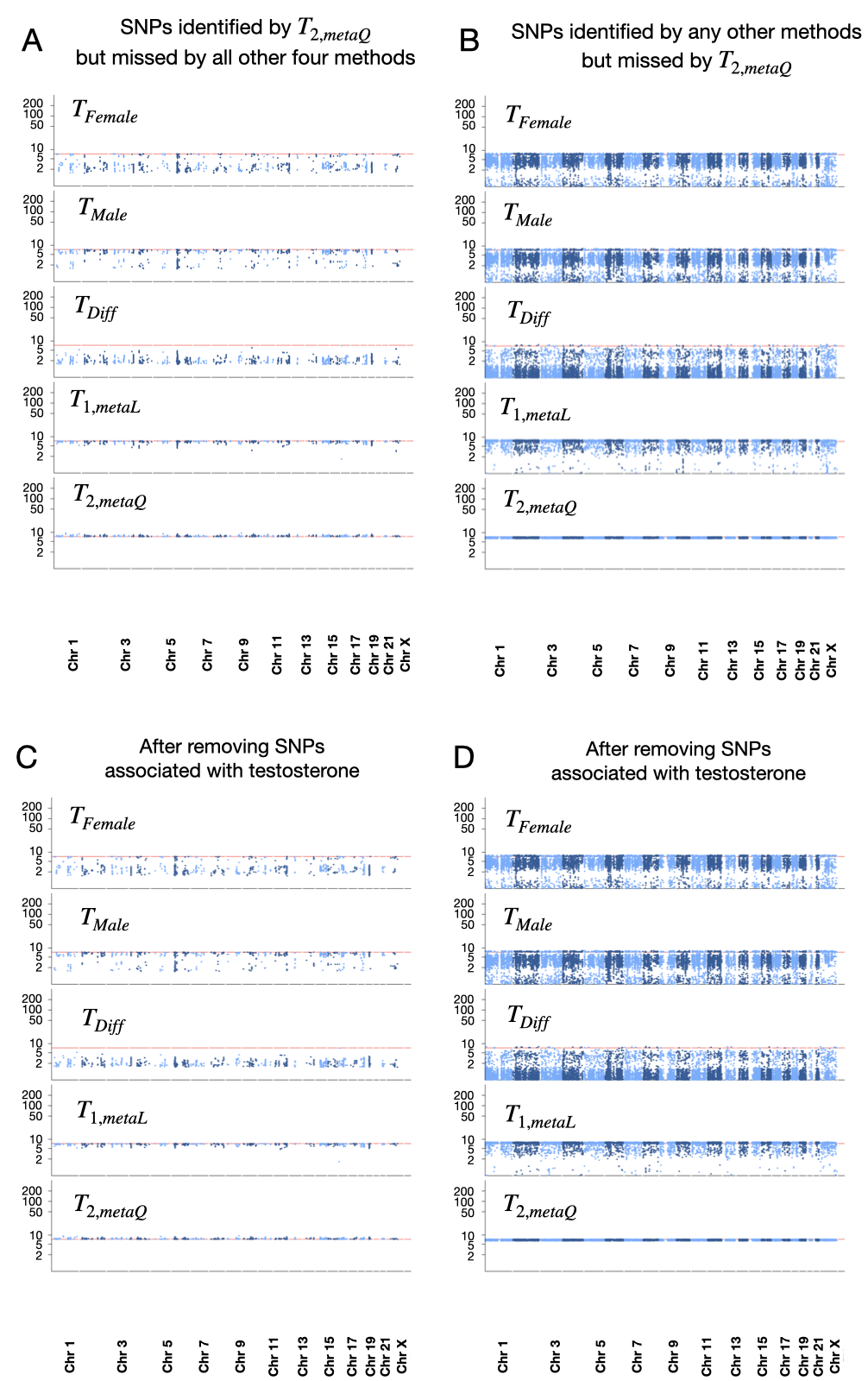

Supplement: S10 Fig — (A) SNPs identified by T2,metaQ but missed by all other four methods across all the 290 traits, (B) SNPs missed by T2,metaQ but identified by any other four methods across all the 290 traits, (C) SNPs identified by T2,metaQ but missed by all other four methods after removing the testosterone, and (D) SNPs missed by T2,metaQ but identified by any other four methods after removing the testosterone. The −log10 p-values (with further log10 transformation on y-axis to aid presentation) are shown for the five association methods, including TFemale (Female-only analysis), TMale (Male-only analysis), TDiff (SNP-sex interaction-only test), T1,metaL (the traditional sex-combined meta-analysis), and T2,metaQ (the omnibus meta-analysis); see Table 1 for method details. The sex-stratified GWAS summary statistics come from the Neale lab’s UK Biobank GWAS round 2, which included a cohort of up to 361,194 participants (194,174 females and 167,020 males). The red horizontal lines indicate the genome-wide significant threshold of 5 × 10^−8^ on the −log10 scale. (TIF) [file pgen.1011221.s018.tif]

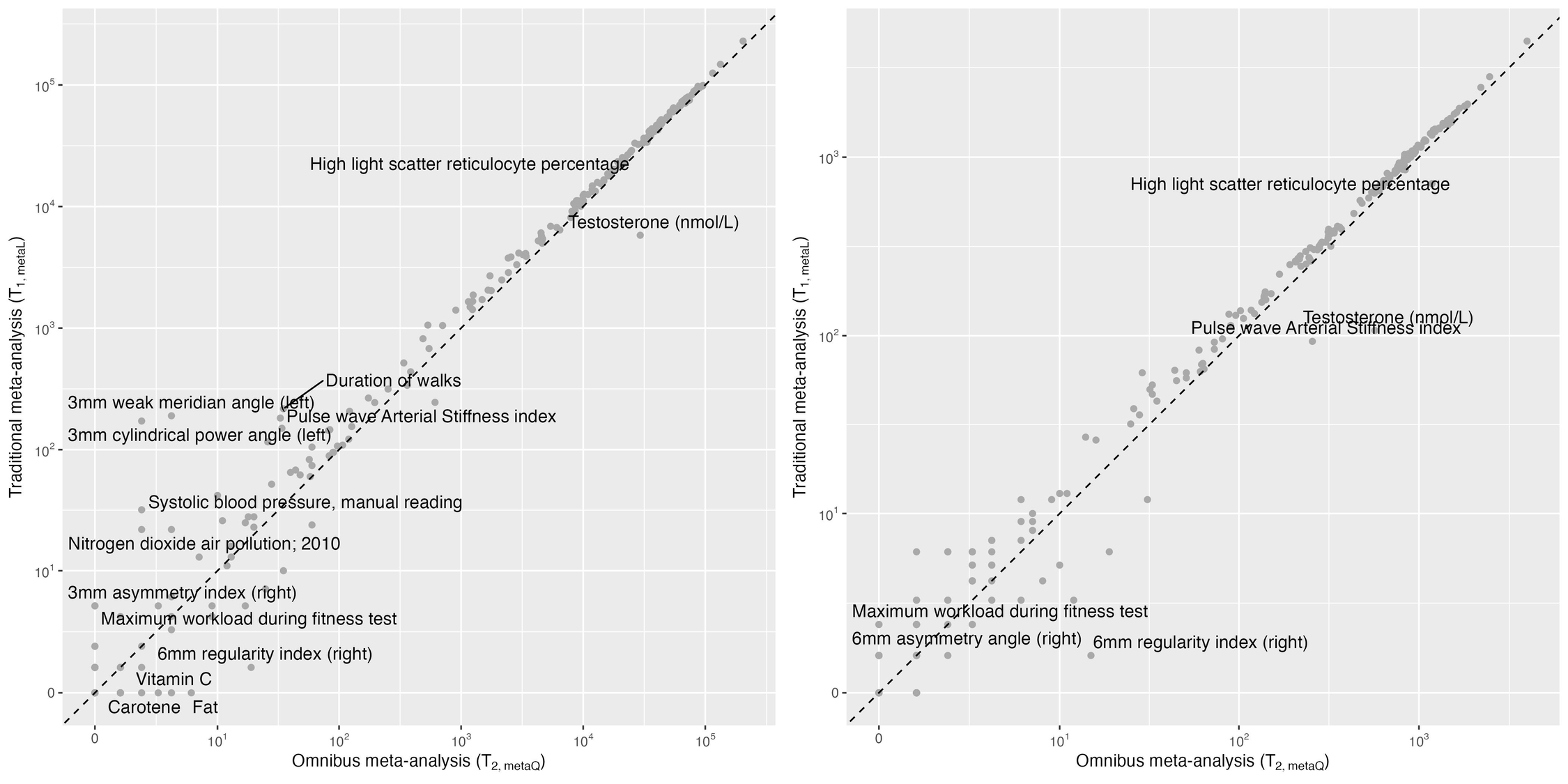

Supplement: S11 Fig — Both x-axis and y-axis are in log10 scale, which provides a zoomed-in look at the traits with smaller numbers of associated SNPs. In (b) linkage disequilibrium is defined with a physical distance of 100kb (Methods and material). The dashed line indicates the reference main diagonal line. The plot with the original scale is provided in Fig 3. (TIF) [file pgen.1011221.s019.tif]

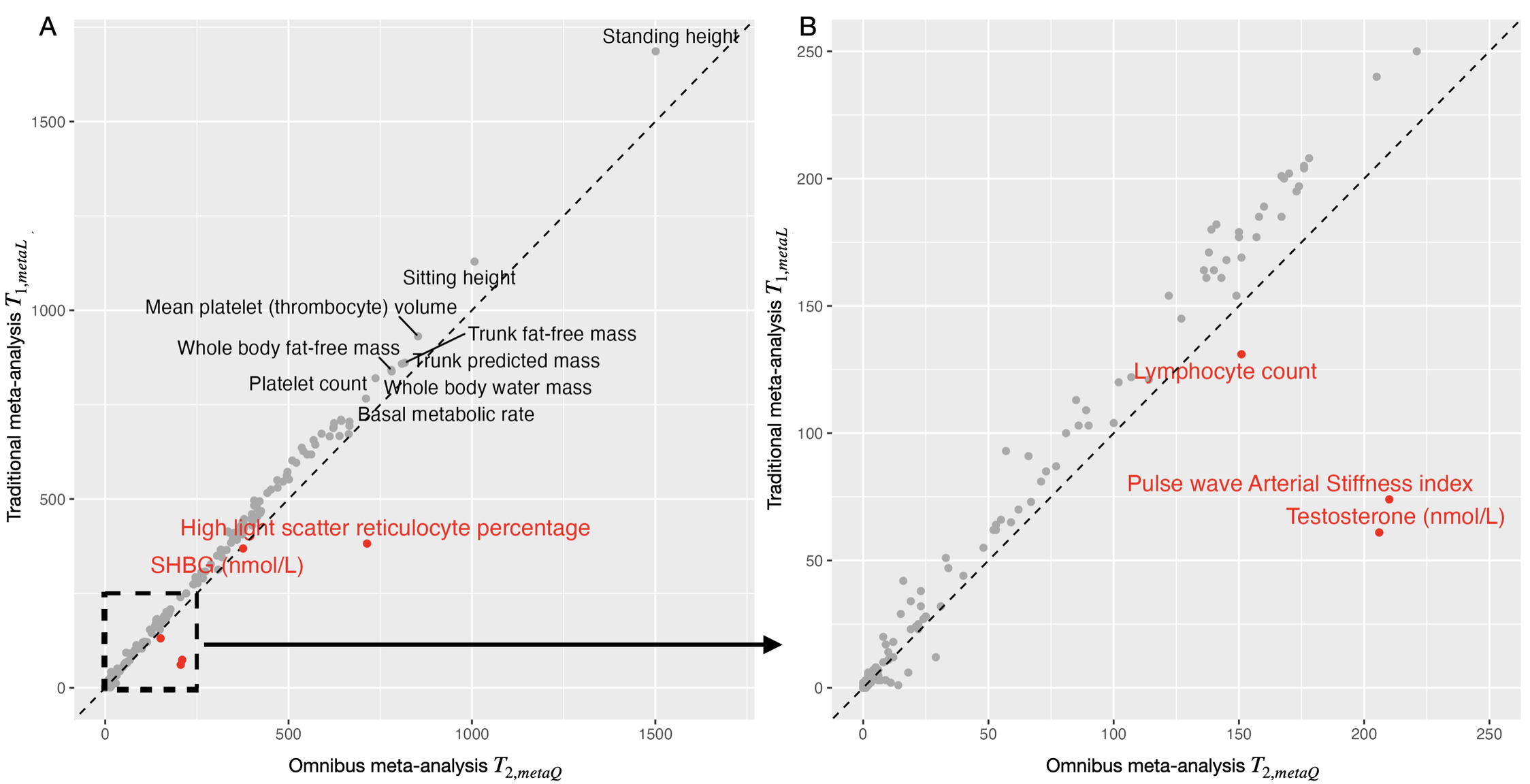

Supplement: S12 Fig — In plots A and B, the x-axis and y-axis represent the numbers of independent SNPs identified by T2,metaQ and T1,metaL, respectively. The linkage disequilibrium is defined with a physical distance of 10MB and r2 > 0.01. The definition for independent SNPs is available in the Materials and methods. Plot B provides a zoom-in view of plot A within the range of (0, 250). Traits that yield more signals in T2,metaQ than in T1,metaL and present at least 50 independent genome-wide significant SNPs in both methods are highlighted in red. Traits with the largest number of associations are annotated. The dashed line indicates the reference main diagonal line. (TIF) [file pgen.1011221.s020.tif]

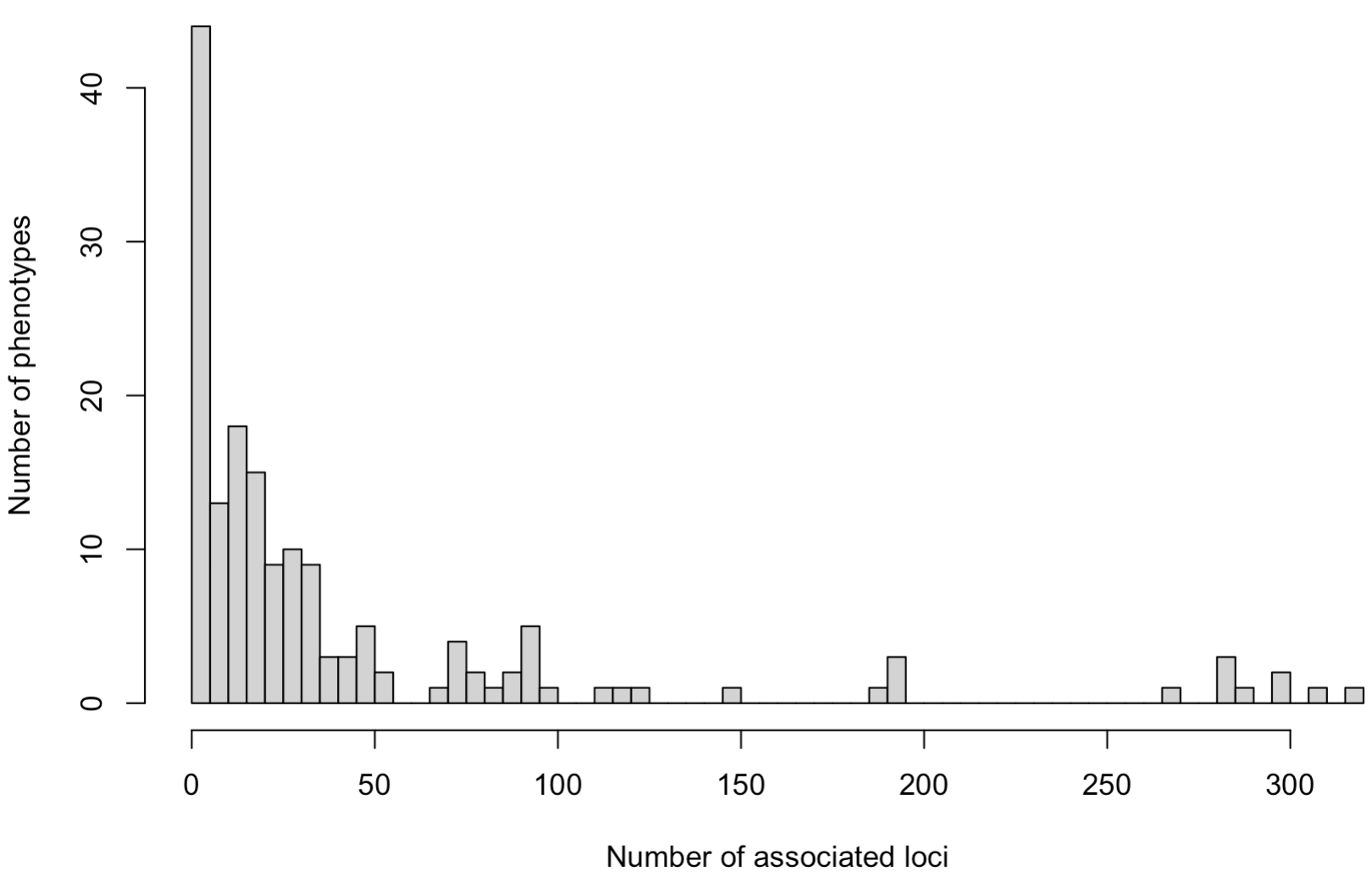

Supplement: S13 Fig — In total, 164 out of the 290 tested phenotypes have one or more associated loci identified by T2,metaQ (the omnibus meta-analysis), but missed by all four of TFemale (Female-only analysis), TMale (Male-only analysis), TDiff (SNP-sex interaction-only test) and T1,metaL (the traditional sex-combined meta-analysis). (TIF) [file pgen.1011221.s021.tif]

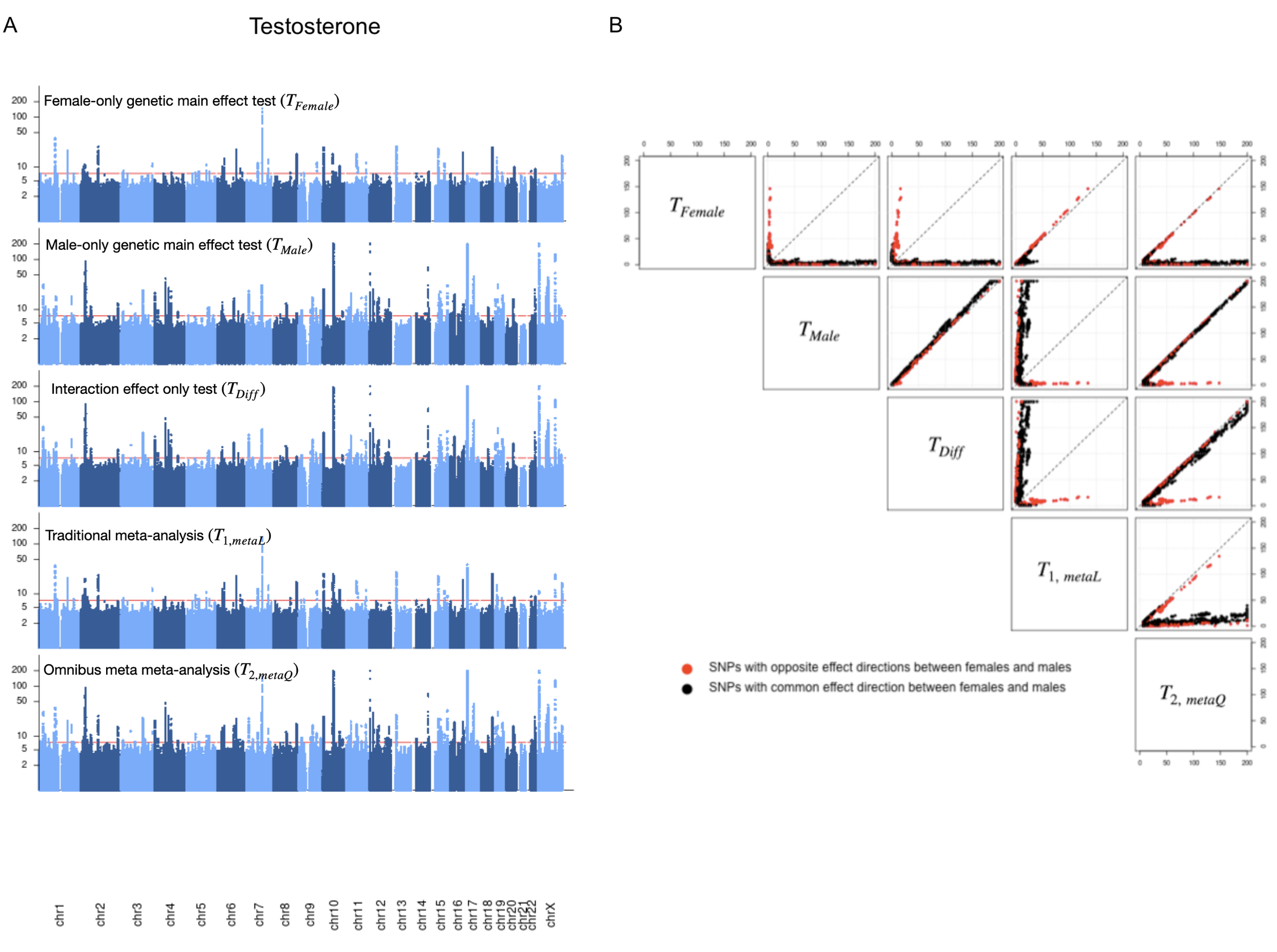

Supplement: S14 Fig — In (A) the −log10 p-values (with further log10 transformation on y-axis to aid presentation) are shown for the five association methods, including TFemale (Female-only analysis), TMale (Male-only analysis), TDiff (SNP-sex interaction-only test), T1,metaL (the traditional sex-combined meta-analysis), and T2,metaQ (the omnibus meta-analysis); see Table 1 for method details. The sex-stratified GWAS summary statistics come from the Neale lab’s UK Biobank GWAS round 2, which included a cohort of up to 361,194 participants (312,102 in testosterone GWAS, 154,364 females and 157,738 males). The red horizontal lines indicate the genome-wide significant threshold of 5 × 10^−8^ on the −log10 scale. In (B), axes depict −log10 p-values for each pair of tests. For simplicity in computation and better visualization, we only included points that achieved genome-wide significance in at least one of the five tests. The −log10 p maximum was truncated at 200 to improve visualization. The dashed line indicates the reference main diagonal line. (TIF) [file pgen.1011221.s022.tif]

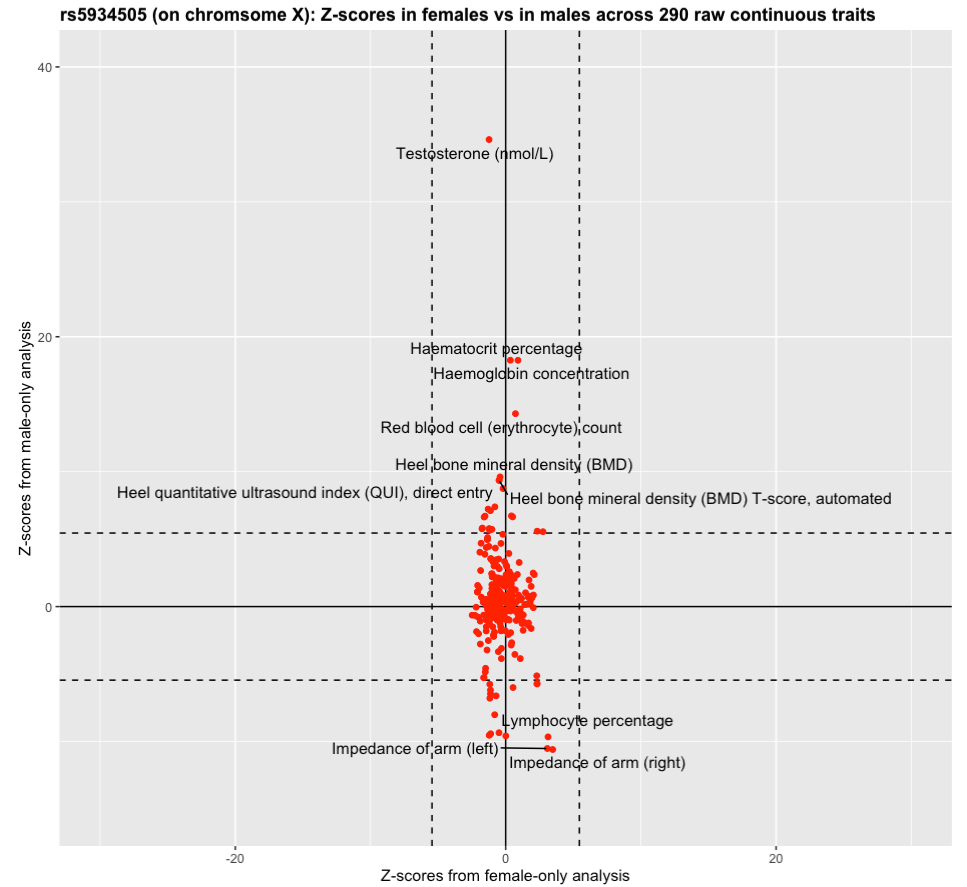

Supplement: S15 Fig — The sex-stratified GWAS summary statistics come from the Neale lab’s UK Biobank GWAS round 2, which included a cohort of up to 361,194 participants (194,174 females and 167,020 males). The dashed lines indicate critical values corresponding to the two-tailed test at genome-wide significant level (5E-8). (TIF) [file pgen.1011221.s023.tif]

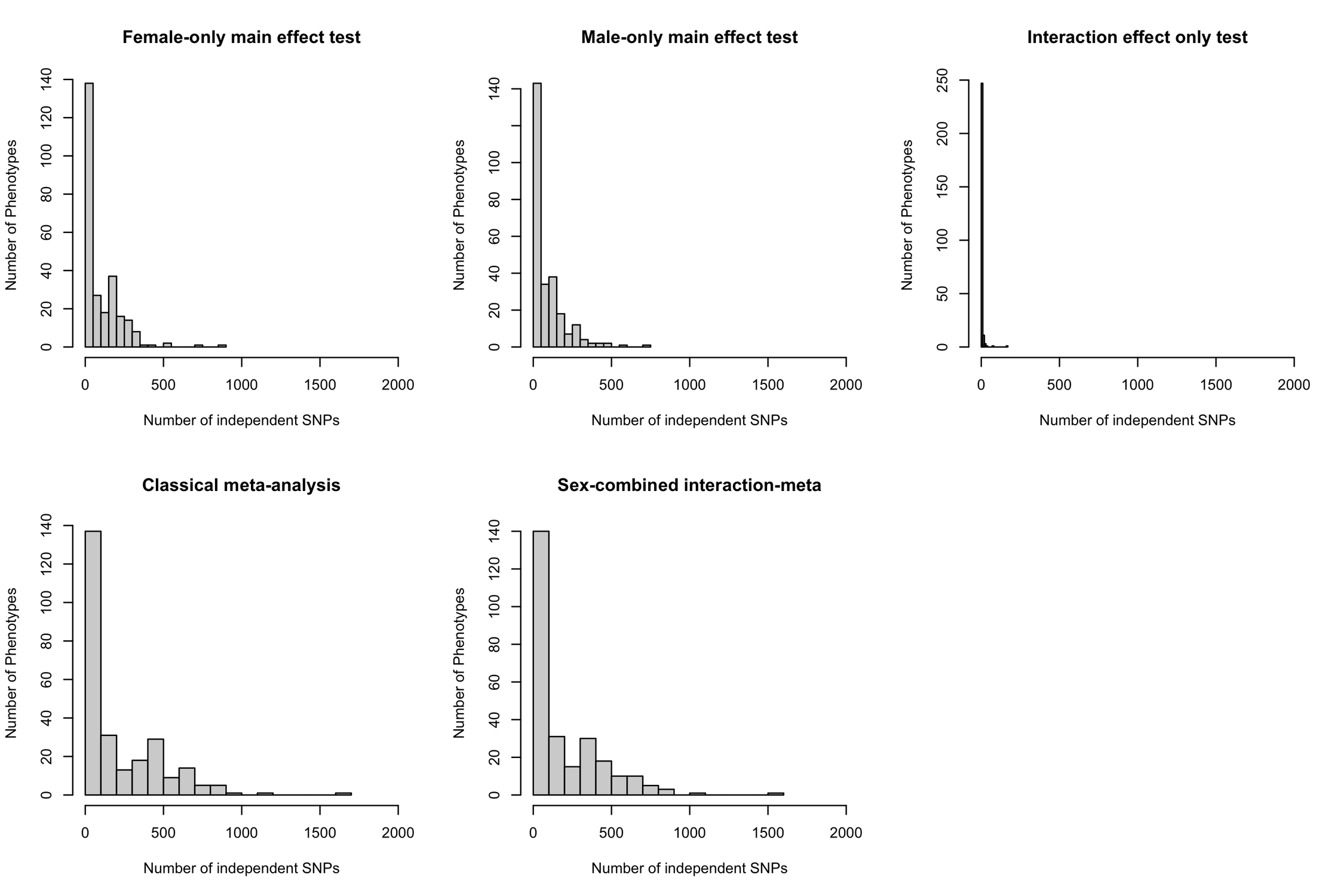

Supplement: S16 Fig — We compare the signals identified by TFemale: Female-only analysis, TMale: Male-only analysis, TDiff: SNP-sex interaction-only test, T1,metaL: Traditional sex-combined meta-analysis, and T2,metaQ: Omnibus meta-analysis. The sex-stratified GWAS summary statistics come from the Neale lab’s UK Biobank GWAS round 2, which included a cohort of 361,194 participants (194,174 females and 167,020 males). We excluded phenotypes with no signals in any of the five methods. (TIF) [file pgen.1011221.s024.tif]
