# Supplementary material for: Better together against genetic heterogeneity: A sex-combined joint main and interaction analysis of 290 quantitative traits in the UK Biobank
Source: PLoS Genet. 2024 Apr 24;20(4):e1011221. doi: 10.1371/journal.pgen.1011221 (PMC11073786; doi:10.1371/journal.pgen.1011221)
Supplement: S1 Table — The T2,metaQ method uniquely identified 1,287 genome-wide significant SNPs, representing 36 independent loci after Linkage Disequilibrium (LD) clumping (Materials and methods). This table presents 16 of these 36 loci with leading SNPs or their dependent SNPs that have been previously reported to be associated either with testosterone phenotypes (A) or with any other phenotypes (B), as reported in the NHGRI-EBI GWAS catalog (Version: e0r2022-11-29, [38]). The βFemale and βMale columns show the sex-specific effect size estimates from the stratified analysis, indicating the estimated effect of each copy of the minor allele. We compared the p-values for the following methods: TFemale: Female-only analysis, TMale: Male-only analysis, TDiff: SNP-sex interaction-only test, T1,metaL: Traditional sex-combined meta-analysis, and T2,metaQ: Omnibus sex-combined interaction meta-analysis. (PDF) [file pgen.1011221.s025.pdf]

S1 Table. 16 testosterone-associated loci uniquely identified by the recommended $T_{2,metaQ}$ but missed by any other methods in the UK Biobank data with previously reported associations in NHGRI-EBI GWAS catalog

A. Previously reported association with testosterone

Lead SNP	CHR	BP (hg19)	Major / Minor Allele	MAF (All/Female/Male)	β_{Female}	β_{Male}	P_{Female}	P_{Male}	P_{Diff}	$P_{1,metaL}$	$P_{2,metaQ}$	Relevant SNP Association from NHGRI-EBI GWAS catalog [PMID]
rs7545893	1	7856267	A / C	(0.196 / 0.195 / 0.197)	-0.015	0.044	9.80E-08	8.40E-03	4.86E-04	1.46E-06	2.09E-08	rs571084788: Testosterone levels [32042192, 35192695]<br>rs1989147: Bioavailable testosterone levels [32042192]
rs6462989	7	40869754	C / A	(0.340 / 0.340 / 0.341)	-0.006	-0.073	6.35E-03	1.95E-07	2.90E-06	3.75E-04	3.17E-08	rs6462989: Total testosterone levels [32042192]
rs56853305	17	27648542	A / G	(0.142 / 0.142 / 0.141)	-0.009	-0.101	5.40E-03	1.03E-07	1.72E-06	2.83E-04	1.47E-08	rs56853305: Total testosterone levels [32042192]
rs34931250	17	66879927	T / C	(0.061 / 0.06 / 0.061)	-0.025	0.075	6.00E-08	6.37E-03	3.18E-04	1.04E-06	1.02E-08	rs34931250: Total testosterone levels [32042192]<br>rs34931250: Testosterone levels in premenopausal women [35192695]<br>rs34931250: Sex hormone-binding globulin levels [32042192]<br>rs34931250: Bioavailable testosterone levels [32042192]

B. Previously reported association with non-testosterone phenotypes

Lead SNP	CHR	BP (hg19)	Major / Minor Allele	MAF (All/Female/Male)	β_{Female}	β_{Male}	T_{Female}	T_{Male}	T_{Diff}	$T_{1,metaL}$	$T_{2,metaQ}$	Relevant SNP Association from NHGRI-EBI GWAS catalog [PMID]
rs141428740	2	27735033	A / G	(0.057 / 0.057 / 0.058)	-0.020	-0.149	5.53E-05	2.15E-07	9.11E-06	1.27E-06	4.26E-10	rs141428740: Triglyceride levels x short total sleep time [31719535]
rs11934762	4	69946149	C / T	(0.476 / 0.475 / 0.477)	-0.010	-0.072	1.31E-05	5.15E-08	3.42E-06	1.93E-07	2.71E-11	rs7668556: Plasma clozapine-norclozapine ratio [30922102]<br>rs6422323: Serum 25-Hydroxyvitamin D levels [32242144]<br>rs11931182: Liver enzyme levels (alkaline phosphatase) [33972514]
rs3936510	5	55860866	T / G	(0.201 / 0.200 / 0.202)	-0.013	-0.079	3.91E-06	1.91E-06	8.68E-05	8.92E-08	2.79E-10	rs3936510: Waist-hip ratio [25673412],<br>rs3936510: Coronary artery disease [29212778],<br>rs3936510: Free cholesterol levels in HDL [35213538]
rs668871	6	160769811	T / C	(0.469 / 0.469 / 0.469)	0.005	0.071	3.16E-02	7.15E-08	7.23E-07	2.57E-03	4.95E-08	rs668871: Sex hormone-binding globulin levels [32042192]
rs1165200	6	25875212	A / G	(0.243 / 0.243 / 0.243)	0.013	-0.071	9.15E-07	4.18E-06	8.52E-08	4.62E-05	1.47E-10	rs17270561: Iron status biomarkers [19084217]
rs77728961	11	118303182	C / T	(0.043 / 0.043 / 0.044)	-0.026	-0.117	2.37E-06	3.52E-04	6.31E-03	1.51E-07	2.46E-08	rs77487830: Gamma glutamyl transferase levels [33462484]<br>rs77535829: Gamma glutamyl transpeptidase [34594039]
rs2601014	15	43756013	C / G	(0.094 / 0.094 / 0.094)	-0.012	-0.123	1.25E-03	5.22E-08	1.36E-06	4.35E-05	2.01E-09	rs2256764: Liver volume [34128465]
rs8033015	15	51061883	G / A	(0.174 / 0.175 / 0.174)	0.011	0.088	2.28E-04	4.81E-07	1.35E-05	7.80E-06	3.53E-09	rs12910301: Chloride levels [29403010]
rs4889333	16	81565383	T / C	(0.493 / 0.492 / 0.494)	-0.011	-0.046	1.37E-06	5.75E-04	9.63E-03	9.49E-08	2.30E-08	rs12933858: Calcium levels [34594039]
rs4782861	16	83978402	G / A	(0.333 / 0.333 / 0.333)	0.008	0.077	9.94E-04	7.15E-08	1.89E-06	3.44E-05	2.21E-09	rs4782861: Platelet count [34594039]
rs6125961	20	48884124	A / C	(0.199 / 0.198 / 0.200)	-0.015	0.049	9.00E-08	3.30E-03	1.51E-04	1.75E-06	8.31E-09	rs6125961: Granulocyte percentage of myeloid white cells [27863252]<br>rs6125961: Monocyte count [27863252]
rs5752776	22	29108229	G / A	(0.329 / 0.329 / 0.328)	-0.006	-0.076	6.89E-03	5.94E-08	9.93E-07	3.57E-04	1.08E-08	rs2078555: Optic cup area [31798171]<br>rs134551: Triglyceride levels [32203549]<br>rs5752776: Alanine aminotransferase levels [33547301]

The $T_{2,metaQ}$ method uniquely identified 1,287 genome-wide significant SNPs, representing 36 independent loci after Linkage Disequilibrium (LD) clumping (Materials and methods). This table presents 16 of these 36 loci with leading SNPs or their dependent SNPs that have been previously reported to be associated either with testosterone phenotypes (A) or with any other phenotypes (B), as reported in the NHGRI-EBI GWAS catalog (Version: e0r2022-11-29, [1]). The β_{Female} and β_{Male} columns show the sex-specific effect size estimates from the stratified analysis, indicating the estimated effect of each copy of the minor allele. We compared the p-values for the following methods: T_{Female}: Female-only analysis, T_{Male}: Male-only analysis, T_{Diff}: SNP-sex interaction-only test, $T_{1,metaL}$: Traditional sex-combined meta-analysis, and $T_{2,metaQ}$: Omnibus sex-combined interaction meta-analysis.

References

1. Buniello A, MacArthur JAL, Cerezo M, Harris LW, Hayhurst J, Malangone C, et al. The NHGRI-EBI GWAS Catalog of published genome-wide association studies, targeted arrays and summary statistics 2019. Nucleic Acids Research. 2019;47(D1):D1005–D1012.
