# Supplementary material for: Better together against genetic heterogeneity: A sex-combined joint main and interaction analysis of 290 quantitative traits in the UK Biobank
Source: PLoS Genet. 2024 Apr 24;20(4):e1011221. doi: 10.1371/journal.pgen.1011221 (PMC11073786; doi:10.1371/journal.pgen.1011221)
Supplement: S2 Table — The T2,metaQ method uniquely identified 1,287 genome-wide significant SNPs, corresponding to 36 independent loci after LD clumping. This table presents 8 of these 36 loci that have never been reported to be associated with any phenotypes in the NHGRI-EBI GWAScatalog [38]. Each locus is represented by its Lead SNP, selected based on the minimum p-value within the locus. The table only included loci with leading insertions/deletions (INDELs). Loci with bi-allelic leading SNPs are reported in Table 2. The βFemale and βMale columns show the sex-specific effect size estimates from the stratified analysis, indicating the estimated effect of each copy of the minor allele. We calculated the p-values for the following methods: Female-only TFemale, male-only TMale, traditional sex-combined meta-analysis T1,metaL, and SNP-sex interaction-only analysis TDiff, omnibus meta-analysis T2,metaQ. The sex-stratified summary statistics used were from Neale’s group (Online Resources). (PDF) [file pgen.1011221.s026.pdf]

S2 Table. 8 novel testosterone-associated loci with leading insertions/deletions (INDELs) uniquely identified by the recommended $T_{2,metaQ}$ but missed by any other methods in the UK Biobank data.

Lead SNP	CHR	BP (hg19)	Minor / Major allele	MAF (All / Female / Male)	β_{Female}	β_{Male}	P_{Female}	P_{Male}	P_{Diff}	$P_{1,metaL}$	$P_{2,metaQ}$
rs61193729	7	98896709	T / TA	(0.060 / 0.060 / 0.061)	-0.025	0.079	3.06E-07	4.76E-03	2.71E-04	4.79E-06	3.78E-08
rs544417459	7	150481093	A / ATCTGTTTGAACTAAT	(0.404 / 0.404 / 0.405)	0.008	0.069	1.19E-03	1.31E-06	2.34E-05	6.24E-05	4.31E-08
rs3062783	10	104979458	GTA / G	(0.247 / 0.247 / 0.247)	-0.013	-0.059	2.23E-06	3.91E-04	6.70E-03	1.48E-07	2.56E-08
rs35822609	11	68102542	C / CT	(0.348 / 0.348 / 0.347)	0.012	-0.049	1.03E-06	5.28E-04	2.32E-05	2.22E-05	1.60E-08
rs375937848	15	64078475	T / TA	(0.343 / 0.342 / 0.343)	-0.008	-0.077	2.32E-03	1.59E-07	3.16E-06	1.05E-04	1.05E-08
rs145130752	16	20061256	T / TA	(0.114 / 0.115 / 0.113)	0.008	0.116	2.31E-02	7.06E-08	7.77E-07	1.75E-03	3.73E-08
rs76634028	17	8189569	G / GT	(0.441 / 0.442 / 0.441)	0.008	0.068	7.59E-04	3.68E-07	8.42E-06	3.10E-05	8.37E-09
rs11383286	19	48365328	AT / A	(0.472 / 0.471 / 0.473)	0.006	0.071	7.64E-03	2.30E-07	3.20E-06	4.81E-04	4.39E-08

The $T_{2,metaQ}$ method uniquely identified 1,287 genome-wide significant SNPs, corresponding to 36 independent loci after LD clumping. This table presents 8 of these 36 loci that have never been reported to be associated with any phenotypes in the NHGRI-EBI GWAScatalog [1]. Each locus is represented by its Lead SNP, selected based on the minimum p-value within the locus. The table only included loci with leading insertions/deletions (INDELs). Loci with bi-allelic leading SNPs are reported in Table 2. The β_{Female} and β_{Male} columns show the sex-specific effect size estimates from the stratified analysis, indicating the estimated effect of each copy of the minor allele. We calculated the p-values for the following methods: Female-only T_{Female}, male-only T_{Male}, traditional sex-combined meta-analysis $T_{1,metaL}$, and SNP-sex interaction-only analysis T_{Diff}, omnibus meta-analysis $T_{2,metaQ}$. The sex-stratified summary statistics used were from Neale's group (Online Resources).

References

1. Buniello A, MacArthur JAL, Cerezo M, Harris LW, Hayhurst J, Malangone C, et al. The NHGRI-EBI GWAS Catalog of published genome-wide association studies, targeted arrays and summary statistics 2019. Nucleic Acids Research. 2019;47(D1):D1005–D1012.
