# Supplementary material for: Better together against genetic heterogeneity: A sex-combined joint main and interaction analysis of 290 quantitative traits in the UK Biobank
Source: PLoS Genet. 2024 Apr 24;20(4):e1011221. doi: 10.1371/journal.pgen.1011221 (PMC11073786; doi:10.1371/journal.pgen.1011221)
Supplement: S3 Table — We compared the p-values for the following methods: TFemale: Female-only analysis, TMale: Male-only analysis, TDiff: SNP-sex interaction-only test, T1,metaL: Traditional sex-combined meta-analysis, and T2,metaQ: Omnibus meta-analysis. The βFemale and βMale columns show the sex-specific effect size estimates from the stratified analysis, indicating the estimated effect of each copy of the minor allele. The sex-stratified GWAS summary statistics come from the Neale lab’s UK Biobank GWAS round 2, which included a cohort of 361,194 participants (343,836 in urate GWAS, 184,755 females and 159,081 males). Columns notated with IRNT show result based on inverse normal transformed urate phenotype. (PDF) [file pgen.1011221.s027.pdf]

S3 Table. 19 bi-allelic SNPs exhibiting genome-wide significant effects on urate in opposite directions in females and males

SNP	CHR	BP (hg19)	INFO	Major / Minor Allele	MAF (All/Female/Male)	β_{Female}	β_{Male}	P_{Female}	P_{Male}	P_{Diff}	$P_{1,metaL}$	$P_{2,metaQ}$	β_{Female} (IRNT)	β_{Male} (IRNT)	P_{Female} (IRNT)	P_{Male} (IRNT)	P_{Diff} (IRNT)	$P_{1,metaL}$ (IRNT)	$P_{2,metaQ}$ (IRNT)
rs6814556	4	10004389	1.000	A / G	(0.268 / 0.268 / 0.268)	-3.184	2.285	4.91E-40	1.44E-15	1.77E-48	5.37E-07	1.14E-52	-0.052	0.031	8.78E-46	1.10E-14	1.17E-52	1.03E-07	1.59E-57
rs6833878	4	10005555	1.000	A / T	(0.268 / 0.268 / 0.268)	-3.169	2.277	1.09E-39	1.80E-15	4.25E-48	6.25E-07	3.14E-52	-0.051	0.031	2.04E-45	1.39E-14	3.02E-52	1.17E-07	4.63E-57
rs3796834	4	10012846	1.000	C / T	(0.279 / 0.279 / 0.279)	-3.704	2.056	8.14E-55	3.47E-13	6.77E-55	3.86E-13	4.73E-65	-0.060	0.028	8.58E-62	2.03E-12	3.35E-60	4.41E-14	2.92E-71
rs3796833	4	10012878	1.000	C / T	(0.279 / 0.279 / 0.279)	-3.714	2.052	4.33E-55	3.86E-13	5.31E-55	2.88E-13	2.81E-65	-0.060	0.028	4.21E-62	2.21E-12	2.41E-60	3.27E-14	1.58E-71
rs1122966	4	10014476	1.000	G / A	(0.28 / 0.279 / 0.28)	-3.813	2.002	4.36E-58	1.32E-12	4.90E-56	1.00E-14	9.63E-68	-0.061	0.027	1.23E-65	7.94E-12	1.03E-61	7.32E-16	1.62E-74
rs6857001	4	10018080	1.000	G / A	(0.28 / 0.28 / 0.28)	-3.837	1.980	7.88E-59	2.24E-12	4.06E-56	3.63E-15	2.93E-68	-0.062	0.027	1.69E-66	1.33E-11	7.05E-62	2.42E-16	3.72E-75
rs3796830	4	10019135	1.000	G / C	(0.279 / 0.279 / 0.279)	-3.742	2.033	6.43E-56	6.18E-13	3.38E-55	1.05E-13	6.58E-66	-0.060	0.027	4.37E-63	3.43E-12	1.15E-60	1.07E-14	2.51E-72
rs28449404	4	10019678	1.000	C / G	(0.279 / 0.279 / 0.279)	-3.738	2.033	8.34E-56	6.21E-13	4.03E-55	1.15E-13	8.64E-66	-0.060	0.027	6.04E-63	3.43E-12	1.42E-60	1.20E-14	3.45E-72
rs10030570	4	10027160	1.000	G / T	(0.28 / 0.28 / 0.28)	-3.687	2.100	1.79E-54	9.98E-14	1.51E-55	1.12E-12	3.03E-65	-0.059	0.028	1.74E-61	6.19E-13	7.29E-61	1.30E-13	1.86E-71
rs6819833	4	10027354	1.000	C / G	(0.28 / 0.28 / 0.28)	-3.687	2.100	1.85E-54	1.01E-13	1.55E-55	1.13E-12	3.14E-65	-0.059	0.028	1.81E-61	6.24E-13	7.61E-61	1.31E-13	1.93E-71
rs6820230	4	10027542	1.000	C / T	(0.28 / 0.28 / 0.28)	-3.691	2.095	1.41E-54	1.15E-13	1.62E-55	9.45E-13	2.76E-65	-0.059	0.028	1.18E-61	7.04E-13	6.95E-61	1.04E-13	1.41E-71
rs6449237	4	10027643	1.000	A / G	(0.28 / 0.28 / 0.281)	-3.689	2.095	1.60E-54	1.15E-13	1.77E-55	9.86E-13	3.13E-65	-0.059	0.028	1.56E-61	7.09E-13	8.54E-61	1.13E-13	1.89E-71
rs6449238	4	10027744	1.000	G / A	(0.28 / 0.28 / 0.28)	-3.689	2.100	1.60E-54	1.01E-13	1.43E-55	1.07E-12	2.75E-65	-0.059	0.028	1.54E-61	6.28E-13	6.85E-61	1.23E-13	1.65E-71
rs7697004	4	10028077	1.000	G / A	(0.28 / 0.28 / 0.281)	-3.686	2.101	1.95E-54	9.77E-14	1.54E-55	1.18E-12	3.25E-65	-0.059	0.028	2.11E-61	6.10E-13	8.07E-61	1.41E-13	2.23E-71
rs7697416	4	10028287	1.000	G / A	(0.281 / 0.28 / 0.281)	-3.659	2.105	1.13E-53	8.62E-14	3.95E-55	2.38E-12	1.66E-64	-0.059	0.028	1.33E-60	5.37E-13	2.24E-60	2.85E-13	1.23E-70
rs7669699	4	10028438	1.000	C / T	(0.28 / 0.28 / 0.28)	-3.693	2.097	1.24E-54	1.10E-13	1.38E-55	9.32E-13	2.32E-65	-0.059	0.028	1.20E-61	6.77E-13	6.60E-61	1.07E-13	1.39E-71
rs9291645	4	10038254	0.997	G / A	(0.281 / 0.281 / 0.282)	-3.654	2.145	1.43E-53	2.97E-14	9.00E-56	4.65E-12	7.31E-65	-0.059	0.029	1.54E-60	1.99E-13	5.07E-61	5.53E-13	5.36E-71
rs6850166	4	10043688	0.997	C / T	(0.281 / 0.281 / 0.281)	-3.709	2.115	4.09E-55	6.94E-14	3.38E-56	7.99E-13	4.89E-66	-0.060	0.029	2.55E-62	4.65E-13	1.31E-61	7.91E-14	2.04E-72
rs4391034	4	10049700	0.996	T / C	(0.282 / 0.281 / 0.282)	-3.688	2.105	1.59E-54	9.03E-14	1.25E-55	1.10E-12	2.46E-65	-0.059	0.028	1.31E-61	5.82E-13	5.71E-61	1.18E-13	1.32E-71

We compared the p-values for the following methods: T_{Female}: Female-only analysis, T_{Male}: Male-only analysis, T_{Diff}: SNP-sex interaction-only test, $T_{1,metaL}$: Traditional sex-combined meta-analysis, and $T_{2,metaQ}$: Omnibus meta-analysis. The β_{Female} and β_{Male} columns show the sex-specific effect size estimates from the stratified analysis, indicating the estimated effect of each copy of the minor allele. The sex-stratified GWAS summary statistics come from the Neale lab's UK Biobank GWAS round 2, which included a cohort of 361,194 participants (343,836 in urate GWAS, 184,755 females and 159,081 males). Columns notated with IRNT show result based on inverse normal transformed urate phenotype.
