# Supplementary material for: Better together against genetic heterogeneity: A sex-combined joint main and interaction analysis of 290 quantitative traits in the UK Biobank
Source: PLoS Genet. 2024 Apr 24;20(4):e1011221. doi: 10.1371/journal.pgen.1011221 (PMC11073786; doi:10.1371/journal.pgen.1011221)
Supplement: Attachment — Submitted filename: Response_to_reviewers.pdf [file pgen.1011221.s028.pdf]

Review Response and Revision Summary

PLOS Genetics (PGENETICS-D-23-01346; EMID:226900acab95270a)

Better together against genetic heterogeneity: a sex-combined joint main and interaction analysis of 290 quantitative traits in the UK Biobank

Dear Drs. Cordell and Zhu, *PLOS Genetics*,

Thank you very much for allowing us to submit a revision, and we sincerely thank the three reviewers for providing us with helpful comments and suggestions. We have addressed and incorporated all of them in the revised manuscript, as detailed in the point-by-point responses on the following pages.

The main features of our revisions include the following:

- Reviewer 1 had only minor suggestions, and we have addressed them all. In particular, we have added three supplementary figures to investigate our results from the perspective of traits, instead of SNPs, to address question number 1.
- Reviewer 2 had three major comments in addition to some minor comments, and we have addressed them all.
  - To address major comment 1, we added a zoomed-in plot to show that the question is a result of the scale and pixel definition of the original Figure 1.
  - To address major comment 2, we moved some of the loci results from the supplementary to the main text, so that the revised main Figure 3 has the SNP and loci results paired next to each other.
  - To address major comment 3, we first clarified that the reason behind the "weird" result in Table 2 is because testosterone exhibits a marked difference in both phenotype mean and *variance* between females and males. We then revised the text to highlight Fig iii panel A in S4 Appendix which shows sex-stratified phenotype distributions.
- Reviewer 3 had only minor comments, and we have addressed them all. In particular, we now provide a Supplementary Data (S1 Data) for the loci that were uniquely identified by $T_{2,metaQ}$ but missed by all the other four tests, across all the 290 phenotypes analyzed, to address question number 3.

We confirm that this research has not been previously published, and it is currently not under consideration for publication in any other journal. All authors have reviewed and approved the revision.

We believe the revision is now suitable for publication in *PLOS Genetics*. Thank you again for the consideration of our work.

Sincerely,

Lei Sun, on behalf of all authors
Department of Statistical Sciences
University of Toronto

Response to Comments from Reviewer #1 on

Better together against genetic heterogeneity: a sex-combined joint main and interaction analysis of 290 quantitative traits in the UK Biobank

The manuscript titled "Better together against genetic heterogeneity: a sex-combined joint main and interaction analysis of 290 quantitative traits in the UK Biobank" compared sex-stratified and combined tests across 290 quantitative traits from UK Biobank data which have not been done. The study revealed that the omnibus meta-analysis outperformed other methods, particularly in scenarios involving heterogeneity in data. It also demonstrated comparable performance to sex-combined main effect with sex interaction tests in simulated data. The authors further illustrated the potency of the omnibus meta-analysis for traits like testosterone.

The work has been well-executed, and the introduction is particularly well-written. However, some minor changes are required.

1. Out of 13 million SNPs, 6 million show significance across 290 traits. How about examining the significant SNPs that are common across multiple traits identified by these methods? Was there any difference observed in this regard?

   Response: Thank you for this insightful suggestion to investigate our results from the perspective of traits, instead of SNPs.

   First, we would to clarify that out of the 6,207,519 associated SNPs, only 1,113,865 are unique, as each could be associated with one or more traits. We apologize for the confusion, and to improve clarity we have revised the text accordingly.

   Second, to answer the question of any differences in the number of associated traits between the different methods, let $p_{j,t,m}$ be the association p-value between SNP j and phenotype t by method m, where j is the index for the 13 million tested SNPs, t is the index for the 290 quantitative traits, and m is the index for the five testing methods and define

$$N_{j,m} = \sum_{t=1}^{290} I(p_{j,t,m} < 5 \times 10^{-8}),$$

   the number of traits associated with SNP j detected by method m. Based on $N_{j,m}$, we then compared the five testing methods through the following three figures (attached on the following three pages respectively).

   - S2 Fig shows the histograms of the number of associated traits for each of the 1,113,865 SNPs, stratified by the five testing methods.
   - S3 Fig is the Manhattan-type of plot for $N_{j,m}$, stratified by the methods.
   - S4 Fig contrasts the $N_{j,m}$ between the methods.

   Results in these three newly added figures suggest that the conclusion of methods comparison remains the same. For example, the sex-stratified methods, T_{Female} and T_{Male} are sub-optimal while the sex-combined approaches $T_{1,metaL}$ and $T_{2,metaQ}$ behave similarly. In fact, S4 Fig and Figure 1 share similar patterns, even though S4 Fig contrasts the number of associated traits while Figure 1 contrasts SNP's association p-values between the different testing methods.

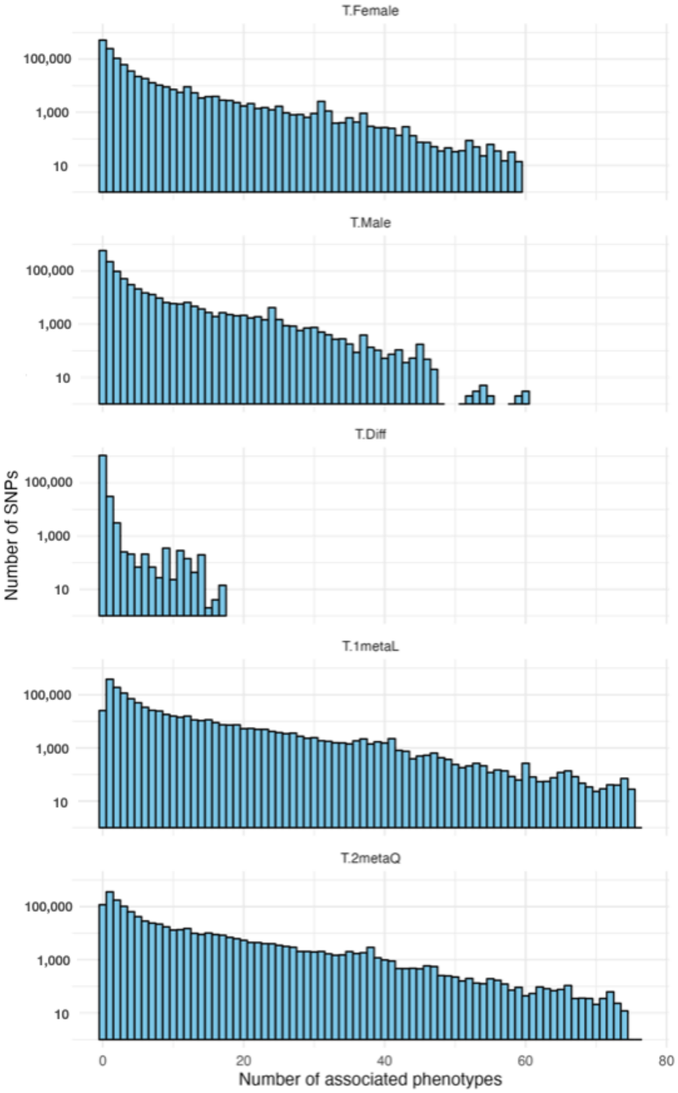

S2 Fig. Histograms of the number of associated traits across 1,113,865 SNPs associated with one or more traits, stratified by the five testing methods. 1,113,865 SNPs are associated with one or more traits identified by any of the five association testing methods: T_{Female} (Female-only analysis), T_{Male} (Male-only analysis), T_{Diff} (SNP-sex interaction-only test), $T_{1,metaL}$ (the traditional sex-combined meta-analysis), and $T_{2,metaQ}$ (the omnibus meta-analysis). The histograms are based on $N_{j,m} = \sum_{t=1}^{290} I(p_{j,t,m} < 5 \times 10^{-8})$, where $I(\cdot)$ is an indicator function, $p_{j,t,m}$ is the association p-value between SNP j and trait t by method m. The y-axis ticks are on the $\log_{10}$ scale for ease of visualization.

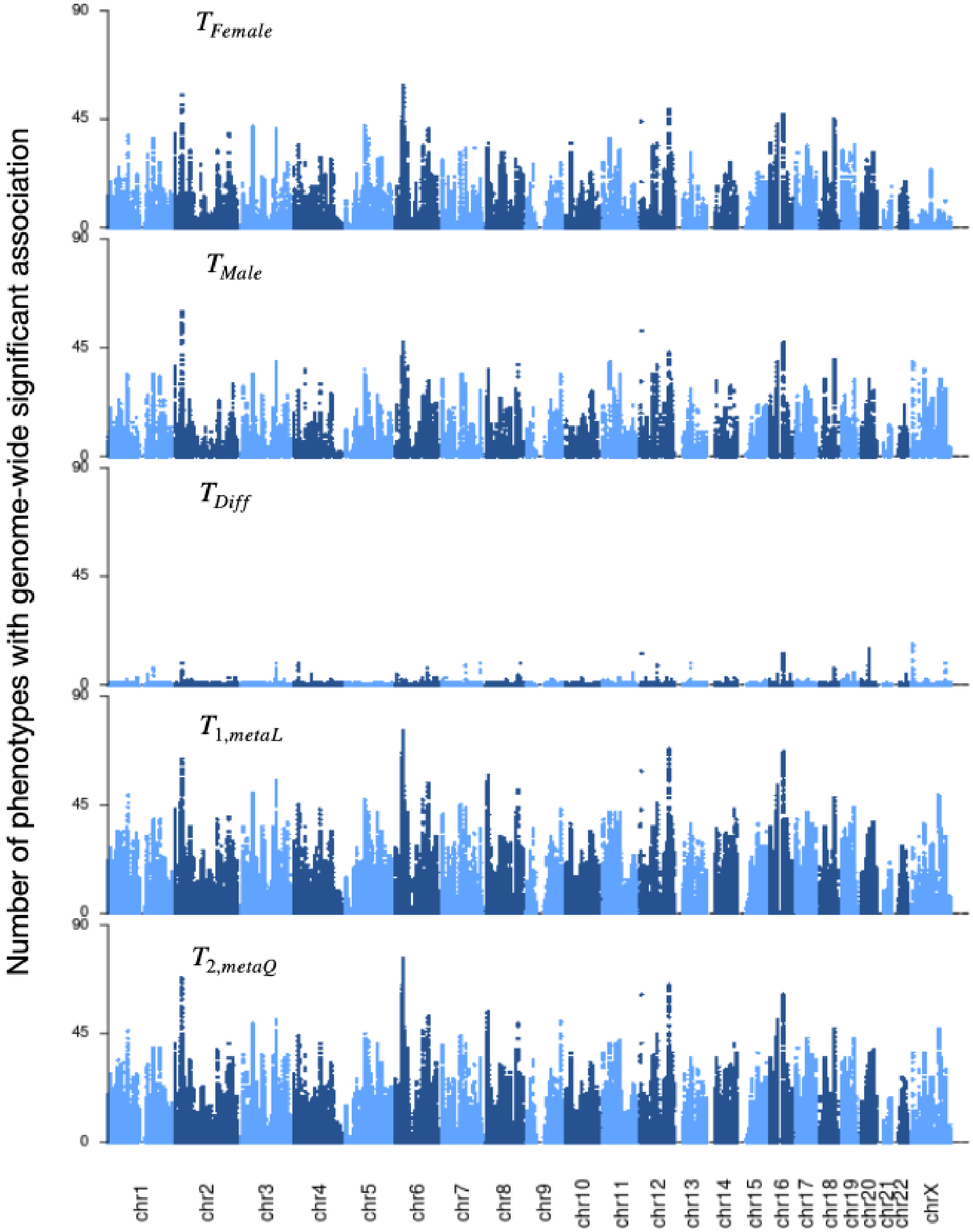

S3 Fig. Manhattan plots of the number of associated traits for each of the 1,113,865 SNPs associated with one or more traits, stratified by the five testing methods. The 1,113,865 SNPs are associated with one or more traits identified by any of the five association testing methods: T_{Female} (Female-only analysis), T_{Male} (Male-only analysis), T_{Diff} (SNP-sex interaction-only test), $T_{1,metaL}$ (the traditional sex-combined meta-analysis), and $T_{2,metaQ}$ (the omnibus meta-analysis). The plots are based on $N_{j,m} = \sum_{t=1}^{290} I(p_{j,t,m} < 5 \times 10^{-8})$, where $I(\cdot)$ is an indicator function, $p_{j,t,m}$ is the association p-value between SNP j and trait t by method m.

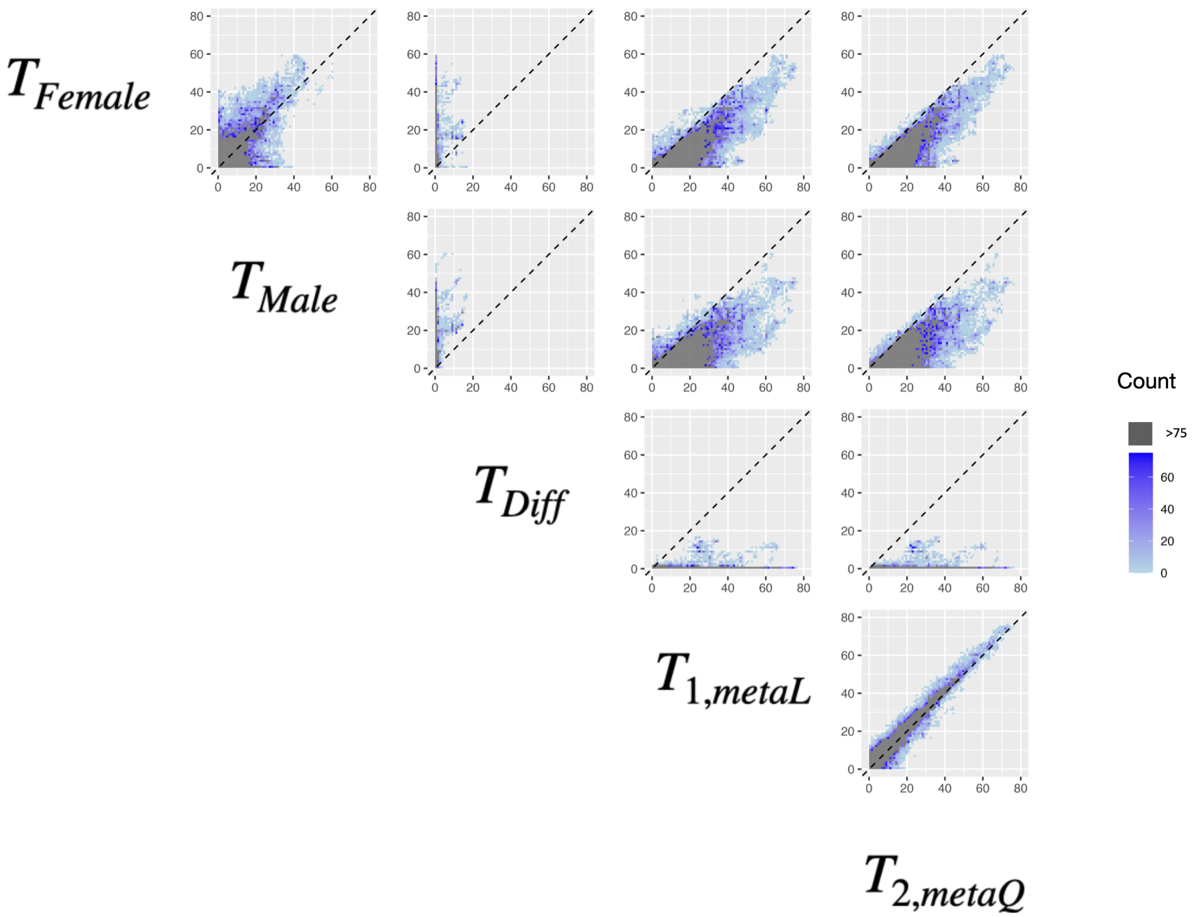

S4 Fig. Pairwise scatter plots of the number of associated traits across the 1,113,865 SNPs among the five testing methods. The 1,113,865 SNPs are the SNPs associated with one or more traits identified by any of the five association testing methods: T_{Female} (Female-only analysis), T_{Male} (Male-only analysis), T_{Diff} (SNP-sex interaction-only test), $T_{1,metaL}$ (the traditional sex-combined meta-analysis), and $T_{2,metaQ}$ (the omnibus meta-analysis). The plots are based on $N_{j,m} = \sum_{t=1}^{290} I(p_{j,t,m} < 5 \times 10^{-8})$, where $I(\cdot)$ is an indicator function, $p_{j,t,m}$ is the association p-value between SNP j and trait t by method m.

Changes: The revised manuscript now includes the above S2 Fig, S3 Fig and S4 Fig as part of the supplementary materials. In the revised text (Page 6), we also conclude that:

Alternatively, focusing on traits, instead of SNPs, we compared the the number of associated traits across 1,113,865 SNPs among the five methods (S2 Fig-S4 Fig), and concluded that method comparison remain consistent.

We also revised the text to emphasize that the 6,207,519 associated SNPs are not unique, referring to SNP-phenotype associations (Page 6).

2. Page 5. The disparity in SNP numbers is significantly larger, with 179,718 SNPs missed by T1,metaL but identified by T2,metaQ and 756,316 SNPs missed by T2,metaQ but identified by T1,metaL across all 346 traits. Please include this information in the results section, not only in the figure legends.

Response: Thank you for this suggestion, and we have revised the manuscript accordingly; we note that the total number of traits analyzed is 290, not 346.

Changes: The revised text in the Results section (Pages 6-7) reads as follows:

Fig 2A shows the stacked-Manhattan plots of the set of 179,718 genome-wide significant SNPs identified by the recommended omnibus meta-analysis $T_{2,metaQ}$ *but missed by the traditional meta-analysis* $T_{1,metaL}$ *across the 290 traits analyzed. It is evident that SNPs identified by* $T_{2,metaQ}$ *have* $-\log_{10} p$*-value range from 7.3 to over 200, but they may have no association evidence based on* $T_{1,metaL}$ *with* $-\log_{10} p$*-value close to zero. On the other hand, although the number of SNPs missed by* $T_{2,metaQ}$ *but identified by* $T_{1,metaL}$ *is 756,316, sustainably larger than 179,718, the two sets of p-values among the 756,316 SNPs are comparable (Fig 2B; also see S5 Fig for distribution of these SNPs with a zoomed-in view within Figure 1).*

3. In Fig 2A and 2B, it is noteworthy that the p-values for T1MetaL never appear to exceed 8-9, particularly when identified solely by T1MetaL. Is there any specific reason for this observed pattern or I misinterpreted from the figure?

Response: Yes, your observation is aligned with the numerical results:

(a) For SNPs identified by $T_{2,metaQ}$ but missed by $T_{1,metaL}$ (Figure 2A), $-\log_{10} p_{1,metaL} \in (0, 7.30]$, $-\log_{10} p_{2,metaQ} \in (7.30, 200)$;

(b) For SNPs identified by $T_{1,metaL}$ but missed by $T_{2,metaQ}$ (Figure 2B), $-\log_{10} p_{1,metaL} \in (7.30, 8.17]$, $-\log_{10} p_{2,metaQ} \in (6.45, 7.30)$.

To provide additional insights supporting the numerical observations, we now also provide analytical arguments in S7 Appendix. We also refer to the analytical insights offered by Reviewer 2 in their comment 8.

In summary, the pattern observed echoes our key message regarding the robustness of $T_{2,metaQ}$ under effect heterogeneity (Page 7): *for SNPs missed by* $T_{2,metaQ}$*,* $T_{2,metaQ}$ *provides comparable association evidence (the last two rows in Fig 2B and D). On the other hand, for SNPs missed by* $T_{1,metaL}$*,* $T_{1,metaL}$ *may provide no association evidence at all (the last two rows in Fig 2A and C), completely failing to capture those SNPs identified by* $T_{2,metaQ}$*.*

Changes: The revised text (Page 7) now reads:

Notably, the minimum $-\log_{10}(p)$ *for such SNPs was 6.45 by* $T_{2,metaQ}$ *while capped at 8.17 by* $T_{1,metaL}$*, matching the expected small cost of* $T_{2,metaQ}$ *having an additional 1 degree of freedom in the absence of sex difference in genetic effect (S7 Appendix).*

4. If possible, could you consider adding additional axis ticks 8 to the y-axis for clarity purpose?

Response: Thank you for the suggestion. Since the $y-$axis in Figure 2 is already crowded, instead of adding more ticks to the y-axis, we now highlight the red reference line at $y = -\log_{10} 5 \times 10^{-8} = 7.3$ by using a thicker dashed line and further clarifying this reference line in the figure legend.

Changes: The revised Figure 2 now has thicker red horizontal lines, and the revised figure legend states that *The red horizontal lines (at* $y = -\log_{10}(5 \times 10^{-8}) = 7.3$*) indicate the genome-wide significant threshold of* 5×10^{-8} *on the* $-log_{10}$ *scale.*

5. In the Discussion section on Pages 9 (278-285), the additional results presented in the appendix should be integrated into the main paper. Given that there isn't any restriction on word limit, their inclusion would enhance the main paper by providing additional evidence of the method's effectiveness.

   Response: Thank you for your suggestion of moving simulation results from the appendix to the main paper. After careful consideration and taking into account Reviewer 2's evaluation, we have decided to retain these simulation results in the appendix.

   Although our simulation studies confirm the effectiveness of $T_{2,metaQ}$ as compared to the other methods, our simulation results largely corroborate findings from prior studies (references [25, 26, 44]). Given that these earlier studies already provide extensive method comparisons under various scenarios, our simulation results act primarily as a validation and sanity check of our method implementation. By keeping the simulation results in the appendix, we ensure that the main work remains focused on large-scale data analysis instead of giving the wrong impression of developing a new method.

   Changes: On Page 11, we add additional references [27-31] in the Discussion section to provide additional supporting evidence in the existing literature, alongside the original references [25, 26, 44].

6. There are two figures with S35. Please check.

   Response: Thank you very much for the careful reading!

   Changes: We have updated all figures labelled S36 (now S16 Fig in the revised manuscript) and higher in the revised manuscript.

7. Page 10 line 313, Check the format "Fig S28" not "S28 Fig"

   Response: We agree that "Fig S28" is a more natural label than "S28 Fig", but the "S+number Fig" is the formatting requirement of PLOS Genetics.

   Changes: NA

8. Check citation number 12

   Response: Again, thank you very much for the careful reading!

   Changes: We have fixed the url information in citation 15 (citation 12 in the original manuscript).

Response to Comments from Reviewer #2 on

Better together against genetic heterogeneity: a sex-combined joint main and interaction analysis of 290 quantitative traits in the UK Biobank

The manuscript reads well and the message is clear. All observations from simulation and real data analyses appear to be in agreement with the expected from the theory, and I do not have any concern with the methodology. Overall, the study does not bring new information per se, as the advantages of the joint test described in this study have already been described in multiple previous publications. Joint test meta-analysis is actually commonly applied in many real data applications investigating gene-environment interaction. However, it provides an interesting large-scale real data validation of these results. Still, some points need to be clarified. My comments are as follows:

Major comments:

1. Results, line 159-160: "it is clear that T1,metaL generally resulted in more associated SNPs than T2,metaQ". Results from Figure 3 seem to be in contradiction with Figure 1 (line 130-131 "the -log10p-values of T2,metaQ appear to be consistently larger than those of the other four methods"). I cannot reconcile the two results: i) Fig1A-B lower right panels show that -log10p of T2,metaQ across all variants and all phenotypes are larger than those from T1,metaL. In that plot, I cannot see a single point above the diagonal. ii) Figure 3 shows that the number of significant signals from T1,metaL is almost always larger than the number of significant signals from T2,metaQ. I might be missing something but I cannot see how this is possible.

   Response: We thank the reviewer for asking these questions, which have helped improve the clarity of the revised manuscript. In summary, the apparent contradiction is due to i) the scale and pixel definition of Figure 1, and ii) a robust method may not be the most powerful method under some alternatives.

   Regarding i), suppose the association evidence for an SNP is $-\log10p = 7$ by $T_{2,metaQ}$, and it is smaller than $-\log10p = 7.5$ by $T_{1,metaL}$. These two values 7 and 7.5, however, will appear to be on the main diagonal line in Figure 1 given the scale and pixel definition of Figure 1.

   To avoid this confusion, we have added a zoomed-in plot as shown below in S5 Fig as a supplementary figure, and we have revised the text accordingly.

   Regarding ii), we emphasize that a robust method may not be the most powerful method under some alternatives. Indeed, as described in the legend of Figure 2 (now also in the main text as suggested by Reviewer 1), across the 290 traits analyzed, the number of associations identified by $T_{1,metaL}$ but missed by $T_{2,metaQ}$ is $N_b = 756,316$, considerable larger than $N_a = 179,718$, the number of associations identified by $T_{2,metaQ}$ but missed by $T_{1,metaL}$ (a total of 5,227,618 SNP-trait associations were identified by both methods).

   However, although $N_b = 756,316$ is four times $N_a = 179,718$, all N_b associations are densely located in the bottom-right corner of area (b) of S5 Fig, where $T_{2,metaQ}$ has smaller but comparable $-\log_{10} p$-values with $T_{1,metaL}$ (also depicted in the last two rows of Figures 2B and 2D), due to the additional 1 df of $T_{2,metaQ}$, as the reviewer pointed out in comment 8. In comparison, the N_a associations in area (a) of S5 Fig could have multi-fold larger $T_{2,metaQ}$ $-\log_{10} p$-values than $T_{1,metaL}$ (also depicted in the last two rows in Fig 2A and 2C).

Changes: We have added the zoomed-in plot S5 Fig as a supplementary figure, and we have revised the text (on Pages 6-7) accordingly to improve clarity: *It is evident that SNPs identified by* $T_{2,metaQ}$ *have* $-\log_{10}$ *p-value range from 7.3 to over 200, but they may have no association evidence based on* $T_{1,metaL}$ *with* $-\log_{10}$ *p-value close to zero. On the other hand, although the number of SNPs missed by* $T_{2,metaQ}$ *but identified by* $T_{1,metaL}$ *is 756,316, sustainably larger than 179,718, the two sets of p-values among the 756,316 SNPs are comparable (Fig 2B; also see S5 Fig for distribution of these SNPs with a zoomed-in view within Figure 1).*

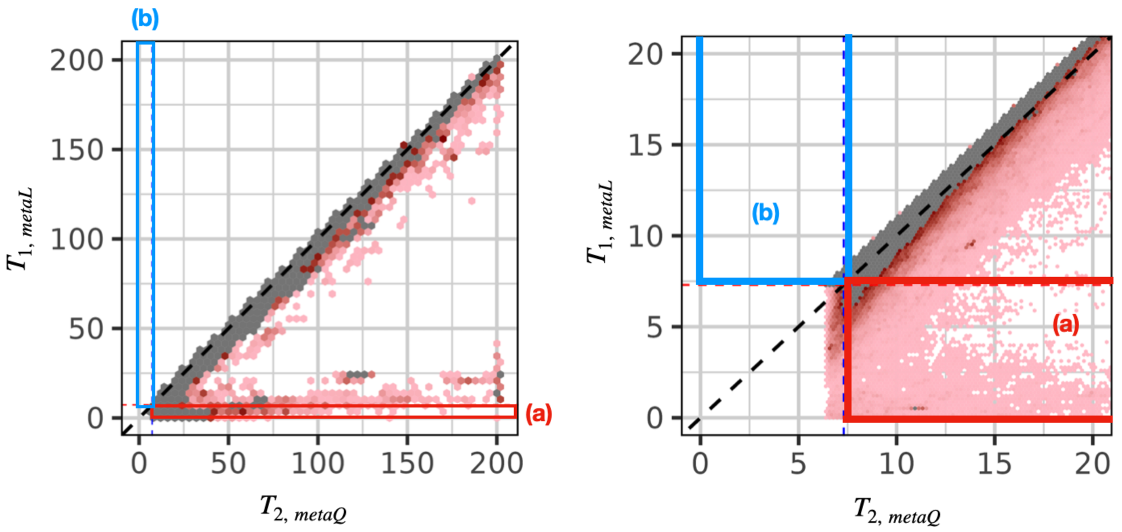

S5 Fig. Left: the bottom-right plot in Figure 1A, comparing $T_{2,metaQ}$ and $T_{1,metaL}$; Right: a zoomed-in view within the range of (0,20) to show that the $-\log10 p$-values of $T_{2,metaQ}$ can be slightly smaller than those of $T_{1,metaL}$. In the red-highlighted area (a), there are 179, 718 SNP-trait associations which were identified by $T_{2,metaQ}$ but missed by $T_{1,metaL}$, and in the blue-highlighted area (b), there are 756, 316 SNP-trait associations which were identified by $T_{1,metaL}$ but missed by $T_{2,metaQ}$.

2. Results, line 174 "29,327 and 5,804". Primary results are expressed in count of total associated variants. This is not much informative because of LD, and results per independent loci should be provided at the same time. Results per loci are currently provided only later on for specific cases (e.g. for testosterone, line 186-195).

Response: Thank you for the comment. The loci results were shown in S12 Fig and discussed in Appendix S4, but we agree with the reviewer that the loci results should be paired with the SNP count results and included in the main text.

Changes: We have revised Figure 3 to include the loci results as shown below, and we have also updated Figure 3 legend to refer to the additional loci results shown in the appendix (by varying LD threshold criterion).

We have also revised the text on Page 7 to summarize the loci results, now reads: *These features are consistent when comparing independent SNPs after LD clumping (Materials and methods; Fig 3B), albeit with four phenotypes, in addition to testosterone, showing more*

significant loci in $T_{2,metaQ}$, for which we provided a comprehensive analysis check in S4 Appendix.

For testosterone, we now also note that the numbers of independent loci corresponding to "29,327 and 5,804" SNPs are 206 and 61, respectively for $T_{2,metaQ}$ and $T_{1,metaL}$.

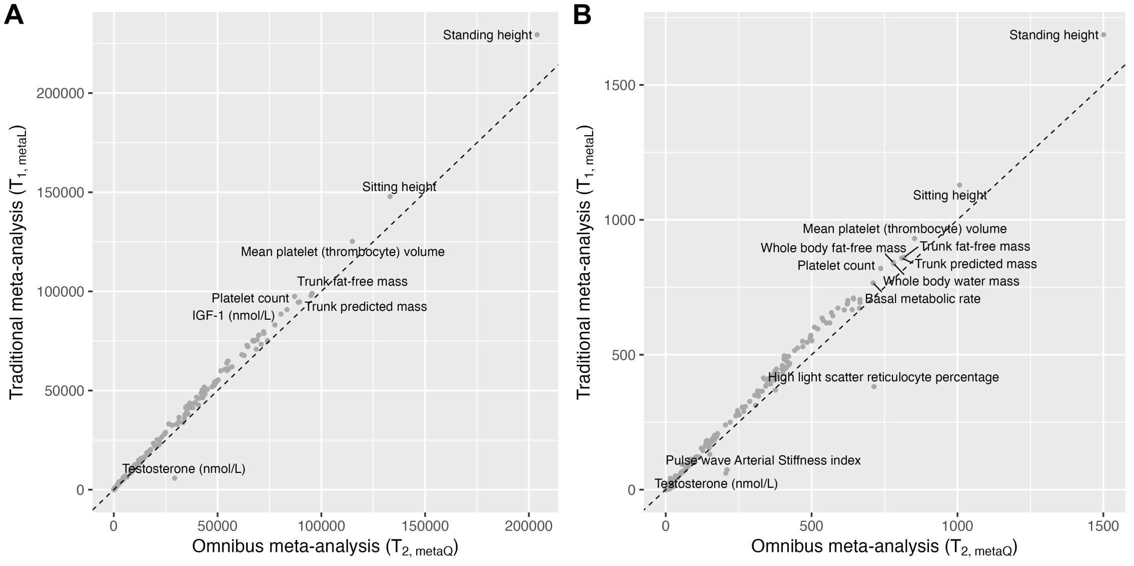

Figure 3 (revised). Comparison of the numbers of genome-wide significant (A) SNPs and (B) independent loci, identified by the traditional meta-analysis $T_{1,metaL}$ (y-axis) and the omnibus meta-analysis $T_{2,metaQ}$ (x-axis) for each of the 290 traits analyzed. The dashed line indicates the reference main diagonal line. See S11 Fig for a log10 transformation of the axes to better visualization traits with smaller numbers of associated SNPs. See S12 Fig for a zoomed-in plot focusing on traits with smaller numbers of associated loci, including testosterone, high light scatter reticulocyte percentage and pulse wave arterial stiffness index; see S4 Appendix for additional analyses of these phenotypes. In (B), the linkage disequilibrium is defined with a physical distance of 10MB and $r^2 > 0.01$ (Materials and methods); see Fig i in S4 Appendix for the loci results after using a different linkage disequilibrium threshold.

3. There is something weird in Table 2: effect sizes for women are substantially smaller than for male. For example, for rs117599084, p-value are similar (8.15e-05 and 1.91e-05), but beta for women is an order of magnitude smaller (0.018 and 0.114). Given the MAF are similar across sex, the only explanation I can think of is that the beta standard errors are smaller for women. How could that be assuming that the sample size for male and female is the same?

   Response: The reason behind this "weird" result is because testosterone exhibits a marked difference in both phenotype mean and *variance* between females (1.1 and 0.4 (unit: nmol/L) respectively) and males (12.0 and 13.8 (unit: nmol/L)). The sex-stratified trait distributions were shown in part A of Fig iii in S4 Appendix and included below for ease of review.

   Thus, despite the similar MAFs and sample sizes between sexes, for sex-stratified association analysis of testosterone, $\widehat{se}(\hat{\beta}_{Male})$ is on average about six times as large as $\widehat{se}(\hat{\beta}_{Female})$ across the SNPs, due to different residual variances in stratified regression models. This case also highlights the importance of using sex-specific variances in the joint regression model, i.e. *not* assuming homoscedasticity.

   Changes: We have revised the text, including legend to Table 2, to highlight part A of Fig iii in S4 Appendix which shows that testosterone exhibits a marked difference in both phenotype mean and *variance* between sexes.

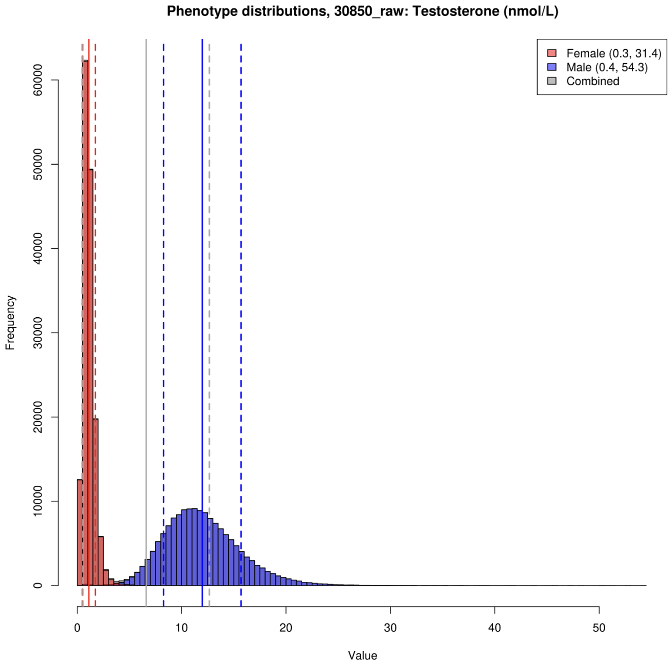

Fig iii (Part A) in S4 Appendix. Sex-stratified testosterone level distributions. The sex-stratified phenotype distributions are based on 154,364 females and 157,738 males included in the Neale lab's UK Biobank GWAS for testosterone level. The vertical solid and dashed lines indicate, respectively, the sample mean and sample mean±1SE. The numbers shown in the brackets are the sample minimum and maximum of the phenotype values.

Minor comments

4. Introduction, line 37-38: "its implementation requires the between-study variance estimate". That would be the case in the presence of sample overlap only, correct?

   Response: We would like to clarify that the between-study variance is a parameter in the random-effects model that is not directly related to sample overlap.

   Specifically, under a random-effects model, the true genetic effects in sub-studies are assumed to be sampled from a normal distribution: $\beta_k \sim N(\beta, \tau^2), k = 1, 2, \cdots, K$, where τ^2 quantifies the extent of the heterogeneity across sub-studies due to differences in study characteristics (DerSimonian and Laird 1986, PMID: 3802833). The between-study variance refers to τ^2, and its estimation may not be reliable when the number of studies is small (Friede et al. 2017, PMID: 27754556).

   Changes: We have revised the text to improve clarity. The revised text (Page 2) reads as follows: *Applying the random-effects meta-approach is also challenging, as its implementation requires estimating τ^2, the parameter that quantifies the extent of between-study heterogeneity. When the number of studies (or groups) is small, the estimate can be unreliable.*

5. Introduction, line 56-58 and further on in the manuscript (e.g. line 94, 165, 183...). "...including interaction effects makes the association analysis more robust to model assumptions in that the test would be considerably more powerful". The term "robust" seems inappropriate and might be confusing. Robustness typically refers to the ability of a statistics to maintain its properties when underlying assumptions are violated. I do not think heterogeneity of genetic signal matches this definition. Instead, the interaction model simply improves the fit to the data, resulting in a gain of power.

   Response: We would like to clarify that in the *absence* of sex "heterogeneity of genetic signal", the regression model with the SNP×sex interaction term is a *misspecified* model. The joint test ($T_{2,metaQ}$ or $T_{2,mega}$) derived from this misspecified model loses power as compared with the test ($T_{1,metaL}$ or $T_{1,mega}$) derived from the correctly specified model, but the power loss of $T_{2,metaQ}$ is capped and p-values are comparable with those of $T_{1,metaL}$. Thus, the joint test is robust to model misspecification.

   Changes: We have revised the text in various places to improve clarity. In particular, we revised the text (Page 3)"*...including interaction effects makes the association analysis more robust to model assumptions in that the test would be considerably more powerful...*" to "*...including interaction effects makes the association analysis more robust to model assumptions in that the joint test remains powerful even in the absence of heterogeneity of SNP effect size between sexes...*".

6. Introduction, line 85-88. "Through a large-scale application, our study aims to provide empirical evidence to show that T2, metaQ is generally comparable to all other GWAS methods and can be more powerful in the presence of genetic effect heterogeneity." This is correct, but I think it would be worth mentioning that stratified analyses remain of high

interest if heterogeneity is suspected-simply to determine where the signal is coming from, but also for secondary analyses such as genetic correlation, or functional annotation enrichment analyses.

Response: Thank you for this suggestion. We emphasized the importance of sex-stratified analyses in the Abstract and Discussion (lines 330-332 of the initial submission), but we agree that the (expanded) message should be further highlighted in the Introduction as well.

Changes: We now add this suggestion to the Introduction section (Page 3:): *"Although $T_{2,metaQ}$ method serves as a powerful first-stage association screening method, subsequent stratified analyses, as well as genetic correlation and functional annotation enrichment analyses, should be applied to fully reveal the landscape of genetic effect heterogeneity and its interpretation.*

7. Results, line 128: "Fig 1A shows the pairwise P-P plots", and further on in the manuscript. "P-P plot" refers to probability-probability plots, while Figure 1 presents -log10(P) plots, more commonly described in the literature using the broader definition of quantile-quantile plot (i.e. QQ-plot).

   Response: Thank you for the suggestion. To avoid confusion, we have decided to use "scatter plots" for figures where each point in the figure refers to the same SNP (or trait), instead of "P-P plots". We use "QQ-plots" for figures where each point in the figure refers to the same quantile.

   Changes: We have replaced all instances of "P-P plots" with "scatter plots".

8. Results, line 147-153: it looks like that the gap between T1metaL and T2metaQ observed in real data perfectly matches the expected cost of having an additional degree of freedom in the absence of an interaction effect (i.e. when the chi-square from the interaction term is null). In short, 8.17 corresponds to a p-value of 6.76e-09 and a 1df chi-square of 33.6. The p-value from a 2df chi-square of 33.6 equals 5e-8, with -log10(5e-8) = 7.3. On the other hand, the -log10(P) = 6.45 from a 2df chi-square corresponds to the expected 5e-8 from a 1df chi-square. This can be easily derived in R using the formula below:
   $pchisq(qchisq(10^-8.17, 1, lower.tail = F), 2, lower.tail = F)$
   $pchisq(qchisq(10^-6.45, 2, lower.tail = F), 1, lower.tail = F)$
   This might be worth mentioning along these observed results.

   Response: Thank you for the analytical insight, which we now include.

   Changes: In the revised manuscript (Page 7), we stated that the numerical results in Figure 2 match the theoretical expectation. Additionally, we added a pointer to a newly added Appendix 7 that shows the "Analytical ranges for p-values of $T_{1,metaL}$ and $T_{2,metaQ}$ in Figure 2". The revised main text now reads (Page 7):

   Notably, the minimum $-\log_{10}(p)$ for such SNPs was 6.45 by $T_{2,metaQ}$ while capped at 8.17 by $T_{1,metaL}$, matching the expected small cost of $T_{2,metaQ}$ having an additional 1 degree of freedom in the absence of heterogeneity of SNP effect size between sexes (S7 Appendix).

Response to Comments from Reviewer #3 on

Better together against genetic heterogeneity: a sex-combined joint main and interaction analysis of 290 quantitative traits in the UK Biobank

Lin and colleagues compared various approaches to identify genetic associations based on sex-stratified genome-wide association studies (GWAS). The approaches include female-specific analysis (testing for associations among women-only at $\alpha_f = 5e-8$), male-specific analysis ($\alpha_m = 5e-8$), difference analysis (testing for the difference between male and female genetic effects, $\alpha_{diff} = 5e-8$), fixed-effect meta-analysis to obtain sex-combined association ("metaL approach", $\alpha = 5e-8$) and an omnibus test (testing for genetic main or GxSex interaction, "metaQ approach", $\alpha_{2df} = 5e-8$). The omnibus test statistic is the sum of female- and male-specific chi-squared statistics and follows a 2df chi-square distribution under the null. This test is equivalent to the "2DF joint test" that is well-known in GxE literature and that that can be derived from a regression model that includes G, SEX and GxSEX interaction. The introduction cites the relevant literature on the topic. They compare the approaches using sex-stratified GWAS summary statistics from the UK Biobank (UKB) for 290 quantitative traits (190K females, 165K males) that had previously been provided by the Neale lab (the authors did not conduct any GWAS). Confirmatory with the literature on the 2DF joint test, the authors find better power, i.e., more associations, by the metaQ approach when there is some underlying dependency of the genetic effects on sex. Without sex dependency, the metaL approach finds more associations due to the decreased degrees-of-freedom. The key point is that metaQ finds any type of association (sex dependent or not) with adequate power whereas the metaL approach only finds those without sex dependency but misses the loci that are dependent on sex. The authors thus advocate usage of the joint test, which is reasonable. This observation is not novel but confirmatory to what is known from the literature. However, this is the first report to apply the joint test to the Neale data set on a large scale. The authors revealed some novel associations for specific traits that have been missed previously. These are of interest for researchers studying the respective traits. While I think that the paper is well done and robust, I still have some minor comments:

1. How is relatedness between males and females accounted for in their tests, particularly in the omnibus test? Is this possible at all or can it just be applied to unrelated males/females? I missed information on whether males/females in this analysis include relatives or not.

   Response:

   Thanks for this important question regarding sample relatedness. Our analysis leveraged the Neale Lab's sex-stratified analysis GWAS summary statistics, and according to their quality controls documentation (`https://github.com/Nealelab/UK_Biobank_GWAS#imputed-v3-sample-qc`) their analyses were restricted to unrelated samples included in the centralized PCA conducted by UK Biobank (indicated by "used.in.pca.calculation").

   In addition to mentioning the above information in the Methods section (Page 15 in the marked-up manuscript), we now also mention it in the revised Results section (Page 6).

   From the method perspective, the omnibus test $T_{2,metaQ}$, as a meta-analysis approach, should be implemented with caution in the presence of sample relatedness:

   (a). When there are related samples **between** females and males, the sex-stratified T_{Female} and T_{Male} test statistics are no longer independent of each other, so $T_{2,metaQ}$ is no longer

χ_2^2 distributed under the null of no association. Similar to the question of meta-analysing overlapping samples in the context of $T_{1,metaL}$ (Lin and Sullivan 2009, PMID: 20004761, Equation [5]), failure to incorporate between-group sample relatedness could inflate type I error. However, how to account for the between-group sample relatedness for $T_{2,metaQ}$ is an open question.

(b). When there are related samples **within** females or males, assuming the sample relatedness was properly adjusted for in the sex-stratified analyses, T_{Female} and T_{Male} are independent of each other. Thus $T_{2,metaQ}$ is χ_2^2 distributed under the null of no association. However, it remains unclear if $T_{2,metaQ}$ and $T_{2,mega}$ are still equivalent to each other, and under what conditions (e.g. how the relatedness was adjusted for within each group).

Both two aspects are important future research directions and we have now added them in the Discussion (Page 12).

Changes: Page 6: In the revised Results section, We now explicitly specify that the analyses were restricted to unrelated samples.

Page 12: We have added the following text: *Moreover, although our analyses were restricted to unrelated individuals [35], it is crucial to approach the implementation of $T_{2,metaQ}$ cautiously if sample relatedness is present. Firstly, the presence of related samples between sexes compromises the independence of T_{Female} and T_{Male}, disrupting the expected χ_2^2 distribution of $T_{2,metaQ}$ under the null. This situation is akin to the challenges in meta-analyzing overlapping samples in the context of $T_{1,metaL}$, where neglecting sample relatedness may increase type I errors [48]. Secondly, within-sex sample relatedness, while it could be readily accounted for in sex-stratified analyses, raises questions about the analytical equivalence between $T_{2,metaQ}$ and $T_{2,mega}$. Thus, addressing these between- and within-sex relatedness in the context of $T_{2,metaQ}$ is of future research interest.*

2. I am missing references to sex-stratified GWAS of body fat distribution that have identified multiple sex-specific loci (including women-only, male-only and even opposite effects).

Response: Thank you for bringing our attention to the body fat distribution phenotype. We have now added multiple references for GWAS of body fat distribution from the GIANT consortium (Shungin D et al., 2015; Heid IM et al., 2010 and Pulit SL et al., 2010), where multiple sex-dimorphic SNPs were identified from sex-stratified GWAS. However, in these papers, although there are SNPs with opposite effect directions between the sexes, we did not find SNPs with genome-wide significant associations in *both* sexes and with *opposite* effect directions.

Changes: In the revised Introduction (Page 2), we have added *"Another example is body fat distribution [11-13], for which approximately one-third of all signals identified from sex-stratified GWAS were found to exhibit sex-specific effects [13]."*

3. The authors highlight associated loci for testosterone (36 loci) and serum urate (1 locus) in the main text and main tables. Have there not been other loci for other traits that could only be identified by the joint test? Any other results are only shown in figures in terms of P values and it is difficult to find "novel" loci across all analyzed traits. Can the authors

provide an overview of identified loci by trait and test and show the index variants of the sex-specific associations for any trait?

Response: Thank you for your question regarding the loci that could only be identified by the $T_{2,metaQ}$ but were missed by all four other 1 d.f. tests across all 290 traits analyzed. We now include this information as a Supplementary S1 Data. The distribution for these numbers is illustrated in newly added S13 Fig, which is also copied below for ease of review. Out of the 290 phenotypes analyzed, 164 phenotypes have one or more associated loci that were identified exclusively by the $T_{2,\mathrm{metaQ}}$ and not by any of the other four 1 d.f. tests. Interestingly, phenotypes with the most signals exclusively from $T_{2,metaQ}$ are primarily anthropometric measurements, including trunk predicted mass (N=319), trunk fat-free mass (N=309), arm fat-free mass (N=299), arm predicted mass (N=298), and whole body water mass (N=289). As these phenotypes are closely related to body fat distribution, this observation aligns with the findings of sex-specific signals discussed in the referenced papers in question 2.

Changes: We now include this information as a Supplementary S1 Data. Additionally, we note that out of the 290 phenotypes analyzed, 164 phenotypes have one or more associated loci that were identified exclusively by the $T_{2,\mathrm{metaQ}}$ and not by any of the other four 1 d.f. tests (Page 8). The distribution for these numbers is illustrated in newly added S13 Fig as below. In the revised text, we add a reference to the newly added Supplementary Data (Page 8).

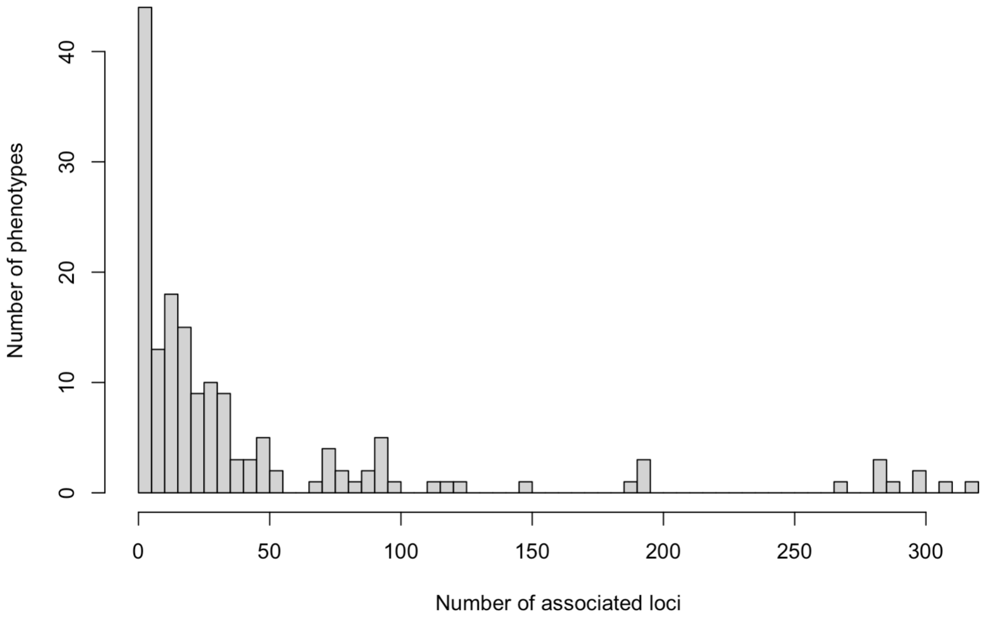

S13 Fig. Histogram of the number of associated loci uniquely identified by $T_{2,metaQ}$ but missed by any of the other four association testing methods across phenotypes. Out of the 290 phenotypes analyzed, 164 have one or more associated loci uniquely identified by $T_{2,metaQ}$ (the omnibus meta-analysis), but missed by all other four testing methods, including T_{Female} (Female-only analysis), T_{Male} (Male-only analysis), T_{Diff} (SNP-sex interaction-only test), and $T_{1,metaL}$ (the traditional sex-combined meta-analysis).

4. 36 independent loci have been identified for testosterone. These include 20 that have not been identified for any other trait before (when compared to GWAScatalog). Table 2 shows 12 of the 20 that were bi-allelic. Table S2 shows the other 8. These are termed "multiallelic SNPs". In fact these are insertions/deletions (INDELs) and no SNPs. A multiallelic SNP has multiple base exchanges at a single position (eg tri-allelic SNPs).

   Response: Thank you very much for this comment.

   Changes: We have changed all occurrences of "multi-allelic leading SNPs" to "insertions/deletions (INDELs)".

5. Table S2 is incorrectly referenced as Table S5 from the main text.

   Response: Thank you for your careful reading and identifying this mistake.

   Changes: We have corrected multiple referencing errors where references for Table S5 should be replaced with Table S2.

6. The tables showing SNP results use T instead of P in the column header. Yet, these are all P values and I would thus replace T by P to avoid confusion.

   Response: Thank you again for your careful reading.

   Changes: On the revised Tables 2 and 3, as well as S1-S3 Tables, we have changed the column headers from T to P. The details of P columns were also added to the Table caption.

7. Table 2: Does the beta refer to the major/minor allele?

   Response: At the bottom of Tables 2 and 3, we had a short note that "The beta-coefficient estimate refers to the estimated effect of each copy of the minor allele". We have now moved this footnote to the caption to avoid potential confusion.

   Changes: Tables 2 and 3 (as well as S1 Table - S3 Table) captions now read *"...The* β_{Female} *and* β_{Male} *columns show the sex-specific effect size estimates from the stratified analysis, indicating the estimated effect of each copy of the minor allele."*
